# Exploring patient safety outcomes for people with learning disabilities in acute hospital settings: a scoping review

**DOI:** 10.1136/bmjopen-2020-047102

**Published:** 2021-05-19

**Authors:** Gemma Louch, Abigail Albutt, Joanna Harlow-Trigg, Sally Moore, Kate Smyth, Lauren Ramsey, Jane K O'Hara

**Affiliations:** 1 Bradford Institute for Health Research, Bradford Teaching Hospitals NHS Foundation Trust, Bradford, UK; 2 NIHR Yorkshire and Humber Patient Safety Translational Research Centre, Bradford, UK; 3 School of Psychology, University of Leeds, Leeds, UK; 4 Lancashire Teaching Hospitals NHS Foundation Trust, Preston, UK; 5 School of Healthcare, University of Leeds, Leeds, UK

**Keywords:** quality in health care, organisation of health services, health services administration & management

## Abstract

**Objectives:**

To produce a narrative synthesis of published academic and grey literature focusing on patient safety outcomes for people with learning disabilities in an acute hospital setting.

**Design:**

Scoping review with narrative synthesis.

**Methods:**

The review followed the six stages of the Arksey and O’Malley framework. We searched four research databases from January 2000 to March 2021, in addition to handsearching and backwards searching using terms relating to our eligibility criteria—patient safety and adverse events, learning disability and hospital setting. Following stakeholder input, we searched grey literature databases and specific websites of known organisations until March 2020. Potentially relevant articles and grey literature materials were screened against the eligibility criteria. Findings were extracted and collated in data charting forms.

**Results:**

45 academic articles and 33 grey literature materials were included, and we organised the findings around six concepts: (1) adverse events, patient safety and quality of care; (2) maternal and infant outcomes; (3) postoperative outcomes; (4) role of family and carers; (5) understanding needs in hospital and (6) supporting initiatives, recommendations and good practice examples. The findings suggest inequalities and inequities for a range of specific patient safety outcomes including adverse events, quality of care, maternal and infant outcomes and postoperative outcomes, in addition to potential protective factors, such as the roles of family and carers and the extent to which health professionals are able to understand the needs of people with learning disabilities.

**Conclusion:**

People with learning disabilities appear to experience poorer patient safety outcomes in hospital. The involvement of family and carers, and understanding and effectively meeting the needs of people with learning disabilities may play a protective role. Promising interventions and examples of good practice exist, however many of these have not been implemented consistently and warrant further robust evaluation.

**Strengths and limitations**

- A key strength is the synthesis of both academic and grey literature materials.
- A further strength is our approach to patient and public involvement and engagement throughout the review process.
- We did not conduct formal quality assessments and are therefore unable to make reflections and comparisons of article quality.

## Introduction

Inequalities in health and inequities in access to healthcare and technologies are a persistent and significant problem.^1–3^ It is clear from previous research that certain demographic factors are associated with increased likelihood of poorer health, and variation in the use of and access to healthcare services.^4 5^

One population that may experience greater vulnerabilities in terms of health and healthcare inequalities are people with learning disabilities. These vulnerabilities might arise as a result of barriers to accessing services and challenges associated with service organisation and delivery.^6^ Learning disabilities are defined as ‘the presence of a significantly reduced ability to understand new or complex information, to learn new skills (impaired intelligence), with a reduced ability to cope independently (impaired social functioning) which started before adulthood, with a lasting effect on development’(https://www.datadictionary.nhs.uk/data_dictionary/nhs_business_definitions/l/learning_disability_de.asp?shownav=1). In this review, we have also drawn from the definition presented in the White Paper *Valuing People*,^7^ which states that learning disability includes the presence of:

- A significantly reduced ability to understand new or complex information, to learn new skills (impaired intelligence), with.
- A reduced ability to cope independently (impaired social functioning).
- Which started before adulthood, with a lasting effect on development.

This broad definition includes adults with autism who also have learning disabilities, but not those with a higher-level autistic spectrum disorder, such as some people with Asperger’s syndrome. Learning disability is the term most commonly used in the UK, although it is recognised as being synonymous with intellectual disability.^8^

In 2013, the final report of a Confidential Inquiry into Premature Deaths of People with Learning Disabilities (CIPOLD) in England was published.^9^ The report found that people with learning disabilities have higher rates of avoidable death compared with the general population, and that avoidable deaths arising from causes relating to poorer quality healthcare were more common in this population. On average, the life expectancy of people with learning disabilities is shorter than the general population.^10^ The 2019 Learning Disabilities Mortality Review (LeDeR) report highlighted that people with learning disabilities died from an avoidable medical cause of death twice as frequently as people in the general population, and that the greatest difference between people with learning disabilities and the general population was in relation to medical causes of death which are treatable with access to timely and effective healthcare.^11^

In the UK, the need for accessible healthcare environments for people with autism is recognised,^12^ and in 2019, the government announced plans to pilot and then roll out learning disability and autism mandatory training for health and care staff in England (https://www.gov.uk/government/consultations/learning-disability-and-autism-training-for-health-and-care-staff). Furthermore, national projects such as Stopping Over-Medication of People with a Learning Disability, Autism or Both (https://www.england.nhs.uk/learning-disabilities/improving-health/stomp/) have addressed issues around medicines practices.

Although there is increasing interest in this important issue from academics, healthcare staff, managers and policy-makers, much of this has focused on health inequalities and healthcare access more generally. What has been lacking to date is a critical examination of this issue as a patient safety phenomenon. This is important, as it opens up new avenues for conceptualising this problem, along with different framings for potential improvement and service development.

There is clear evidence that people with learning disabilities may be more at risk in terms of patient safety in hospital as well as known challenges around recognising and reporting patient safety incidents in this population.^13–15^ Therefore, the need to bring together what is known about the safety of people with learning disabilities receiving healthcare, is clear.

In this review, we aimed to produce a narrative synthesis of published academic and grey literature focusing on people with learning disabilities in an acute hospital setting. We limited this review to the hospital setting because we were particularly interested in the care people with learning disabilities receive in a setting that may be predominantly related to physical health. We aimed to generate evidence that may facilitate the development of more tailored patient safety interventions for people with learning disabilities in an acute hospital setting. Our specific objectives were to:

1. Understand patient safety and adverse events in this population.
2. Explore protective factors and potential explanatory mechanisms.
3. Identify patient safety interventions, improvement initiatives, recommendations and examples of good practice.

## Methods

A scoping review was considered the most suitable approach to produce a comprehensive, yet broad overview of the topic area.^16 17^ We used Arksey and O’Malley’s^18^ six stage framework and subsequent amendments to guide the review.^16 19^ The stages include: (1) identifying the research question(s); (2) identifying relevant research studies; (3) selecting relevant research studies; (4) charting the data; (5) collating, summarising and reporting the study findings and (6) consulting with key stakeholders throughout the process. The review has been drafted in line with the Preferred Reporting Items for Systematic Reviews and Meta-Analyses (PRISMA) Extension for Scoping Reviews.^20^ We developed a broad search strategy, informed by the PRISMA Extension for Systematic Reviews with a Focus on Health Equity (2012).^21^

### Patient and public involvement and engagement

Our review team includes a lay representative (coauthor) who provided input into the protocol, reviewed the search strategy and helped develop materials for the wider patient and public involvement and engagement approach. We invited stakeholders to contribute search terms and assist in identifying grey literature. Stakeholders included representatives from the Yorkshire Quality and Safety Research Group patient panel, representatives from the NIHR Yorkshire and Humber Patient Safety Translational Research Centre Citizen Participation Group, and healthcare staff.

### Eligibility criteria

The ‘Population-Concept-Context’ approach was used to specify study characteristics.^16^ Inclusion and exclusion criteria were developed and iteratively refined as the review progressed. Studies reporting on patient safety, adverse events, protective factors, potential explanatory mechanisms, intervention and improvement initiatives, recommendations and good practice examples related to these topic areas were eligible. There was no restriction of study design, quantitative and qualitative methodologies were eligible for inclusion, and we limited the search to English language only.

#### Inclusion criteria

- Articles that report on people with learning disabilities as the core focus (population). Articles may use terms synonymous with learning disability such as intellectual disability or refer to a condition related to learning disability, for example, autism (autism and learning disabilities are often coassociated^22 23^), attention deficit hyperactivity disorder (high comorbidity for learning disabilities and attention deficit hyperactivity disorder^24^), or Down’s syndrome.
- Articles that investigate adverse events, patient safety, protective factors, potential explanatory mechanisms, patient safety interventions and improvement initiatives, recommendations and good practice examples (concept).
- Articles relating to patients receiving care in an acute hospital setting (context). No restriction on age.
- Articles relating to any country (context).
- Study type: No restriction—qualitative, quantitative, mixed methods, case studies, primary research, retrospective review, systematic or scoping reviews/integrative reviews/meta-synthesis.
- Language: Only articles published in the English Language due to lack of resources for an interpreter.

#### Exclusion criteria

- Articles relating to primary care settings and inpatient mental health settings.
- Articles focusing on patient experience/satisfaction.
- Articles focusing on a specific drug treatment or procedure without a non-learning disability comparison group.

### Information sources and search strategy

#### Academic literature search

The search terms built on terms used in prior reviews framed around the eligibility criteria.^15 25–28^ An initial limited search of MEDLINE was conducted (online supplemental appendix 1). The search strategy was peer reviewed by a Knowledge and Information Librarian reviewer using the Peer Review of Electronic Search Strategies (PRESS),^29^ and reviewed by academic researchers (patient safety), lay representatives and learning disability healthcare professionals. Following the initial search, all four included databases were searched: MEDLINE, CINAHL, PsycINFO and Web of Science from 2000 to 12 March 2021. The time period searched from was 2000 in line with the seminal publication of ‘To Err is Human: Building a Safer Health System’ as this publication arguably launched the modern patient safety movement.^30^ The search was organised in three blocks: block 1—terms relating to learning disability (combined with OR); block 2—terms relating to adverse events and patient safety (combined with OR); block 3—terms relating to acute hospital setting (combined with OR). Blocks 1–3 were combined with the AND function. The reference lists of included articles were assessed, and we handsearched targeted journals including: the British Journal of Learning Disabilities, Journal of Learning Disabilities, Journal of Intellectual Disability Research, BMJ Quality and Safety, Journal of Patient Safety, Health Expectations, BMC Health Services Research, BMJ Open.

10.1136/bmjopen-2020-047102.supp1 Supplementary data

#### Grey literature search

The grey literature search included suggestions made via stakeholder input, such as terms to search, known publicly available materials and specific organisations to search online (online supplemental appendix 2). We searched using the same combinations of terms relating to our eligibility criteria (eg, ‘patient safety and learning disability’, ‘learning disability and hospital’, ‘learning disability and adverse events’). All the online materials returned were initially screened according to title/summary information. In addition, the first 100 pages of Google, Google Scholar and all materials returned from OpenGrey and Royal College of Nursing Database were screened. The latest date for grey literature searches was 10 March 2020.

10.1136/bmjopen-2020-047102.supp2 Supplementary data

### Study selection

Identified articles were collated in reference software (EndNote) and duplicates removed. Study selection involved two levels of screening: (1) title and abstract (2) full text. Three reviewers (GL, AA and JH-T) screened at title and abstract level according to the eligibility criteria, and 10% were independently checked to assess agreement. Articles that appeared to be eligible were screened at full-text level. When a full text was unavailable, authors were contacted directly. We were unable to obtain two full texts. Two independent reviewers assessed the full-text articles (GL and AA) and at this stage the reasons for exclusion were recorded. There were no discrepancies between reviewers regarding the eligibility of articles. Two authors carried out the grey literature search (GL and AA), and one author independently screened the potential grey literature for inclusion (SM), and 10% were independently checked to assess agreement.

### Charting the data

Standardised data collection forms were developed and information from academic articles and grey literature material were collated into separate data collection forms, which were piloted prior to full data extraction.^19^ For academic articles, key data were extracted including: publication year, publication type, country, study design, population and summary information relating to adverse events, patient safety, protective factors, potential explanatory mechanisms, intervention or improvement initiatives, recommendations and good practice examples. Following piloting, two reviewers (AA and JH-T) independently extracted the data from all included articles, and one reviewer checked 10% of the data extracted for consistency (JOH).

Study quality was not assessed as the aim of the review was to synthesise the emerging evidence rather than assess quality of individual articles. The grey literature data collection form was amended from the research article data collection form. Three reviewers (SM, AA and LR) independently extracted the data from all included publications using the adapted data collection form, and one reviewer checked 10% of the data extracted for consistency (JOH).

### Data synthesis

Data were collated in two spreadsheets, one for academic articles and one for grey literature. A narrative synthesis followed to develop a narrative description of the findings and to highlight concepts that key findings could be organised around.^31 32^ Authors (GL, AA, SM, LR and JOH) held meetings to discuss the key findings and generate concepts.

## Results

**Figure 1:**
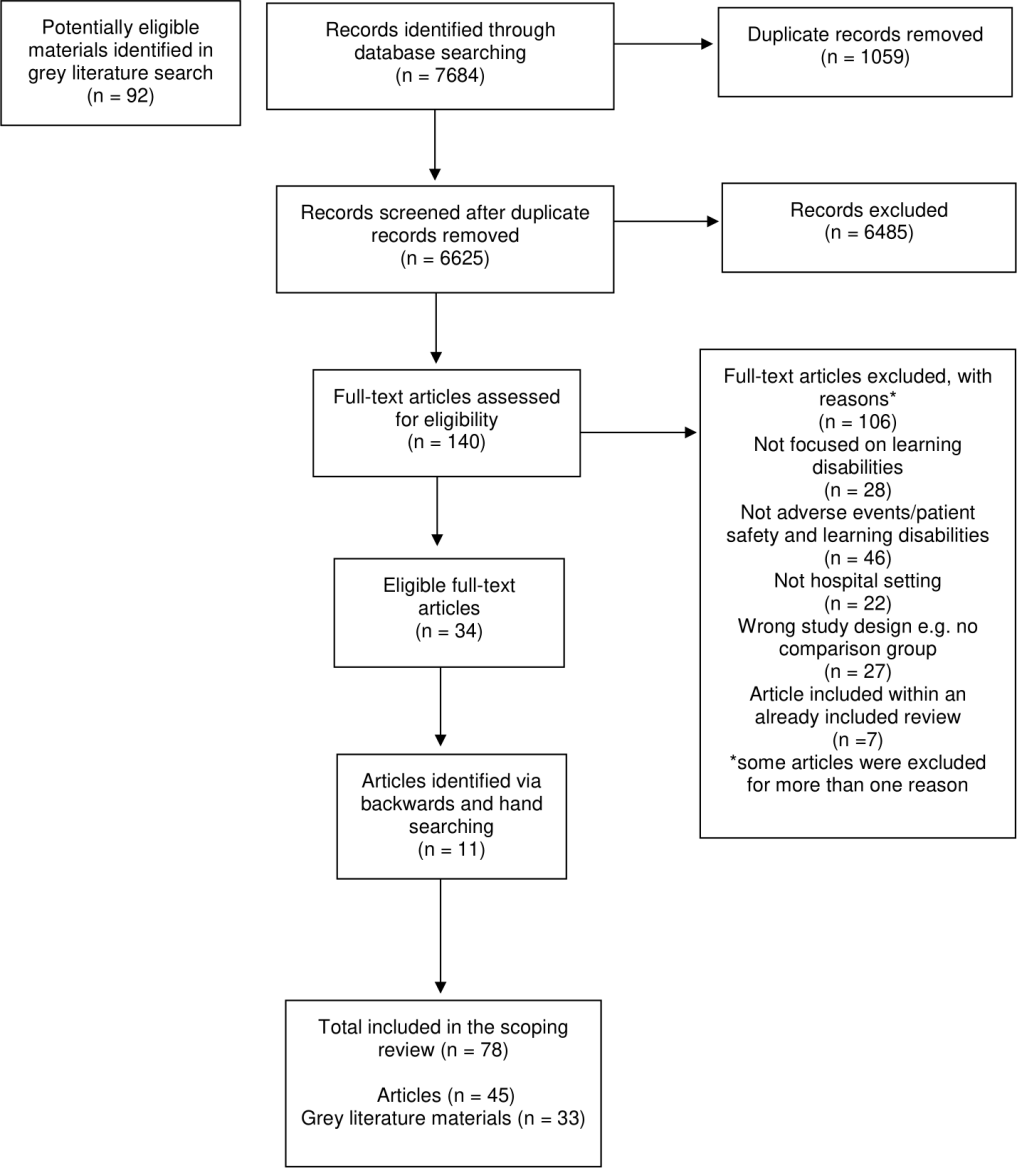
PRISMA flow diagram. PRISMA, Preferred Reporting Items for Systematic Reviews and Meta-Analyses.

Title and abstract screening identified 140 articles eligible for full-text screening. Where studies appeared in review articles that met the eligibility criteria, these were not analysed separately and excluded (n=7). Thirty-four articles were eligible for inclusion in the review. A further 11 articles were included via backward and handsearching. In total, 45 articles were included (see figure 1). The grey literature search identified 92 potentially eligible materials, and 33 were included.

### Summary characteristics

Characteristics of included articles and grey literature materials are displayed in online supplemental appendices 3 and 4.

10.1136/bmjopen-2020-047102.supp3 Supplementary data

10.1136/bmjopen-2020-047102.supp4 Supplementary data

Of the academic articles, 19 related to paediatric patients, 5 to pregnant women/infant outcomes, 4 to adult patients, 2 to healthcare staff, 1 to healthcare staff and carers, 1 to parents or guardians and 13 articles related to hospital patients/setting more generally or did not specify the participants in more detail. All studies and reviews were conducted in high-income countries. Eighteen articles were from the USA, 13 were from the UK, 8 were from Australia, 2 were from Canada, 2 were from Taiwan and 1 each was from Hungary and The Netherlands. Twenty-one articles were retrospective and/or cohort studies, 6 were a type of literature review, 4 were discussion/opinion pieces, 3 articles used mixed methods, 3 improvement projects, 2 were qualitative, 2 were featured/special interest articles, 1 commentary, 1 case study, 1 short report and 1 secondary analysis. Fourteen articles referred specifically to intellectual disability, 10 to Down syndrome, 8 to learning disability, 5 to autism, 3 to intellectual and developmental disability, 2 to communication disability, 1 to developmental delay, 1 to cognitive impairment and 1 to attention deficit hyperactivity disorder. Throughout the results section we use the same terms as those used in the original articles and grey literature materials.

### Key concepts

Our data synthesis generated six concepts: (1) adverse events, patient safety and quality of care; (2) maternal and infant outcomes; (3) postoperative outcomes; (4) role of family and carers; (5) understanding needs in hospital and (6) supporting initiatives, recommendations and good practice. We present these concepts below and specify how they map onto the three review objectives.

#### Objective 1: understand patient safety and adverse events in this population

##### Adverse events, patient safety and quality of care

Six articles concentrated on either specific types of adverse events, quality of care or had a patient safety focus (see table 1). A systematic review of the experience of iatrogenic harm during hospitalisation for children with intellectual disability found that there are specific aspects of hospitalisation that expose children with intellectual disability to harms that are preventable, avoidable and not experienced to the same extent by children without intellectual disability.^15^ Also focused on children, a further study indicated that children with pre-existing cognitive impairment received lower doses of analgesia and sedation medication, although the authors acknowledged it was not clear whether this was due to lower requirements or inadequate assessment.^33^

**Table 1:** An overview of articles relating to adverse events, patient safety and quality of care

Author and year	Aim and method/article type	Key findings
Mimmo (2018)^15^	Narratively synthesise evidence concerning the experience of iatrogenic harm during hospitalisation for children with ID. *Systematic review and narrative synthesis.*	16 papers provided evidence around: the assumptions of HCWs; reliance on parental presence and the need for HCWs to understand the IDs experienced by children in their care. There are specific aspects of hospitalisation that expose children with ID to harms that are preventable, avoidable and not experienced to the same extent by children without ID.
Best (2019)^33^	To compare current analgesia and sedation management practices between critically ill children with pre-existing CI and critically ill neurotypical children, including possible indicators of therapeutic efficacy. *Secondary analysis of prospective data.*	CI patients received significantly lower doses of analgesia and sedation medication than those without CI. However, it was unclear if this was due to lower requirements or vulnerabilities to inadequate assessment.
Hemsley (2016)^34^	Identify research reports regarding investigating the care or safety of adults with communication disabilities in hospital, and to analyse findings according to the generic model of patient safety. *Literature review.*	Patient safety incident and adverse event reporting lacked detail for example, little demographic, descriptive, temporal and categorical information about the patient and staff and how events were detected. Successful advocacy affected outcomes, although where advocacy was ignored outcomes were worse. Stories of adverse events themes included; suffering, isolation due to not having a method to communicate with nurses, a perilous care situation culminating in an adverse event and protective carers discovering or forestalling an adverse event.
Kelly (2016)^36^	Compare 30-day hospital readmission rates of people with and without LDs. *Retrospective audit.*	No significant difference in 30-day readmission rates for patients with and without LDs. However, 69% of readmissions of those with LDs were potentially preventable. Those with more profound LDs were at greater risk of experiencing poor quality care and experiencing readmission within 30 days, and this group comprised over half of the PPRs.
Shah (2009)^37^	Review outcomes and toxicity of chemotherapy for acute lymphoblastic leukaemia in children with DS. *Cohort.*	Patients with DS spent more days in hospital, particularly during the induction phase of treatment.
Oulton (2018)^38^	Compare and identify factors that facilitate and prevent children and young people with and without LDs and long term conditions from receiving equal access to high-quality hospital care and services. *Mixed methods.*	Two key themes; national variation and staff uncertainty. Lack of knowledge about policies, systems and practices at an organisational level to support care of children and young people with LDs. Considerable variation between hospitals ranging from those appearing to have few or no systems, policies or practices in place specifically for this group, with partial systems, policies or practices in place and those with a cohesive and comprehensive level of provision. There was a lack of standardised systems in place for communicating that an individual has an LD. Also a distinct lack of systems in place for recording that an individual involved in a complaint or the subject of clinical incident has an LD.

CI, cognitive impairment; DS, Down syndrome; HCWs, healthcare workers; ID, intellectual disability; LD, learning disability; PPRs, potentially preventable readmissions.

An integrative review investigated the care and safety of adults with communication disabilities in hospital and included a significant amount of studies specifically focused on intellectual disability.^34^ The review concluded that patient safety incident and adverse event reporting lacked detail, and that successful advocacy affected outcomes, suggesting that when advocacy was ignored outcomes were worse. The review reported adverse event themes, including isolation due to limited methods to communicate with nurses, and that carers had a protective role in uncovering or preventing adverse events. Two primary studies reported within the aforementioned integrative review warrant further attention.^14 35^ First, a mixed-methods study concluded that hospitals often lack effective systems for identifying patients which makes monitoring safety incidents difficult. This study also highlighted that staff do not always readily identify patient safety issues or report them, with incident reports commonly focused on events causing immediate or potential physical harm, and that safety issues were mostly related to delays and omissions of care.^14^ Second, a study underpinned by a conceptual framework on patient safety aimed to identify factors that promote and compromise the implementation of reasonably adjusted healthcare services for patients with intellectual disabilities. This study emphasised the importance of ward culture, staff attitudes and staff knowledge in ensuring that hospital services are accessible to vulnerable patients.^35^

A study assessing readmission found no significant difference in 30-day readmission rates for people with and without learning disabilities, but that 69% of readmissions of people with learning disabilities were potentially preventable,^36^ and a study examining outcomes and toxicity of chemotherapy for acute lymphoblastic leukaemia in children with Down syndrome found that these patients spent more days in hospital particularly during the induction phase of treatment.^37^

In a mixed-methods study, staff survey respondents reported feeling less confident about managing challenging behaviour and always delivering safe care to children and young people with learning disabilities, compared with children and young people without learning disabilities, as well as reporting that the environment was less safe for meeting the needs of children and young people with learning disabilities compared with those without.^38^

A wealth of grey literature further evidenced vulnerabilities in terms of adverse events, quality of care and patient safety for people with learning disabilities.^9 11 39–52^ This included influential reports such as the 2013 CIPOLD^9^ and the subsequent LeDer programme annual reports, which evaluate the LeDer programme.^11 45–47^

#### Maternal and infant outcomes

Five articles examined maternal and infant outcomes utilising a retrospective and/or cohort design, either focusing on women with intellectual and developmental disabilities,^53–55^ intellectual disability and/or self reported learning difficulties,^56^ and attention deficit hyperactivity disorder.^57^ Higher rates of complications such as pre-eclampsia,^53 55–57^ preterm birth,^55 57^ low birth weight,^55 56^ and labour interventions including induction and caesarean^53 55 57^ were reported. One study reported higher prevalence rates for hospital admission and emergency department visits during all critical postpartum periods for those with intellectual and developmental disabilities, and higher risk of repeated hospitalisations.^54^

A survey led by Patient Experience Network (not-for-profit organisation) and CHANGE (national human rights organisation) supported by NHS England, aimed to capture the experience of parents with learning disabilities.^58^ Training for health professionals to better support parents with learning disability and improving accessibility to services were highlighted as essential.

#### Postoperative outcomes

The postoperative experience featured significantly in the systematic review of the experience of iatrogenic harm during hospitalisation for children with intellectual disability included within this review (referred to in adverse events, patient safety and quality of care findings).^15^ Thirteen further articles reported on postoperative outcomes.^59–71^ The majority of articles included data relating to Down syndrome,^59–61 63–65 68 69^ followed by intellectual disability,^62 67 70^ developmental delay^66^ and autism spectrum disorder.^71^ Increased rates of complications^60 62 63 66 69 70^ were reported in a number of studies. However, in one study comorbidities rather than Down syndrome were a greater risk factor for complications when adjusting for other covariates,^59^ and after propensity matching, another study also focusing on patients with and without Down syndrome, found no significant variation regarding rates of postoperative complications.^64^ Furthermore, one study focusing on risk factors for major complications related to percutaneous endoscopic gastrostomy placement in children concluded that when adjusting for other variables, intellectual disability was not a significant risk factor.^67^

A longer length of stay was reported in four studies^60 62 63 70^ with one study reporting a similar length of stay for those with Down syndrome compared to those without,^65^ and one study reporting that patients with autism spectrum disorder had a shorter length of stay and were less likely to experience complications.^71^ In one study mortality and major complication rates were lower for patients with Down syndrome.^65^ Similarly, further studies also focusing on Down syndrome found mortality and medical complications to be significantly lower for patients with Down syndrome with no significant differences in terms of surgical complications,^68^ and lower odds of in-hospital death for patients with Down syndrome when controlling for other factors such as risk category and premature birth.^61^ In four studies no differences in mortality were reported,^62–64 66^ and in one study children with intellectual disability had a higher risk of 30-day mortality compared with children with no intellectual disability.^70^

#### Objective 2: explore protective factors and potential explanatory mechanisms

##### Role of family and carers

Reliance on parental presence as a protective factor from poor care quality was emphasised in the systematic review of the experience of iatrogenic harm during hospitalisation for children with intellectual disability included within this review (referred to in objective 1 findings).^15^ Furthermore, a primary study included within an already included literature review^34^ (referred to in objective 1 findings) warranted further attention within this concept. The qualitative interview study explored paid carers’ roles in supporting adults with developmental disability and complex communication needs and described how paid carers are often motivated by perceived responsibility for safety, well-being and communication, but that their role can sometimes be blurred with nursing and family carer roles.^72^

Five further articles highlighted the significant role of families and carers. A meta-narrative approach to understand the experience for the parent of a child with intellectual disability in hospital resulted in a synthesis of 11 studies. A working model for professional parent partnership was developed which reinforced the importance of hospital/multidisciplinary approaches to care centring on the child, understanding previous negative experiences and negotiating care and shared learning to lessen reliance on parental presence.^73^ A further review evaluated how hospital systems respond to adults with intellectual disability, their families and carers. Key themes included: individual fear of hospital encounters, reliance on paid family carers for basic needs and advocacy, responsibilities and staff knowledge, skills and attitudes.^25^

A key finding from a qualitative study with medical practitioners concluded that practitioners make limited use of ‘reasonable adjustments’ and turned to caregivers to facilitate communication and manage behaviours likely to upset hospital routines.^74^ A mixed-methods study aiming to identify factors that affect carer involvement for people with intellectual disabilities in acute hospitals presented a model for clarifying carer involvement that sought to highlight the degree to which carers are ‘workers’ contributing to basic nursing care, and the degree to which carers are experts or non-experts.^75^ The authors suggested that making these two aspects explicit might facilitate staff to understand carer contributions more comprehensively. Finally, a quantitative case note audit demonstrated poor performance across a range of elements of hospital care for people with learning disability.^6^ One notable positive finding of the audit was that in most cases family or carers were involved in discharge planning.^6^ However, the thoroughness of this was questioned as many carers were not signposted to an assessment of their needs prior to discharge.

In terms of grey literature, a doctoral thesis which investigated emergency healthcare from the perspective of the carers of people with learning disabilities, highlighted the relationship staff had with both service users and carers as fundamental to a high-quality service.^76^

##### Understanding needs in hospital

Six articles had content relating to the needs of people with learning disabilities in hospital.^77–82^ One article concluded that to ensure nurses do as much as possible to identify risk they must recognise prejudices and overcome them, develop further understanding of learning disabilities and acknowledge the rights of people with learning disabilities and collaborate with carers and professionals.^78^ Similarly, a literature review around communication, recognised the importance of collaborating effectively with carers, as well as access to personally held written health information, inter-agency communication, devoting time to communication and access to communication tools and aids.^79^ A literature review assessing evidence around the promotion of health, safety and welfare of adults with learning disabilities in acute care emphasised the importance of care provision, communication, staff attitudes, staff knowledge, supporters and carers and the physical environment.^77^ Crucially, communication was highlighted as a fundamental issue, such that people with learning disabilities often have difficulty communicating their needs. The literature review presented strategies and resources that may support this such as videos, accessible booklets, augmentative and alternative communication and pictures/symbols.

To help improve the inpatient experience of hospital patients with autism, a survey of parents and guardians with qualitative and quantitative items highlighted the need for an individualised approach to assess and accommodate needs.^81^ This approach was taken in a case study that described the plan of care for a patient with moderate level of learning disability scheduled for a tonsillectomy. The report gave a specific example of how investing time to understand a patient’s need can improve experience.^82^ When the patient’s details were being checked, the door knocked into the patient’s chair as staff entered the room for equipment, and this exacerbated the patient’s anxiety. This was acknowledged quickly and a do not disturb sign was placed on the door.

An article aiming to familiarise the paediatric nurse with autism and create a resource for successful inpatient treatment put forward key themes such as change is a challenge, consistent caregivers, safe environment, encouraging family involvement, ways of communicating, emotional triggers and reward systems and multidisciplinary team from admission.^80^ Indeed, the NHS long-term plan published in 2019,^83^ emphasised that the whole NHS will improve its understanding of the needs of people with learning disabilities and autism, with plans in place for staff to receive training on supporting people with a learning disability and/or autism alongside the implementation of national learning disability improvement standards. Furthermore, the government response to the consultation on learning disability and autism training for health and care staff also published in 2019, underlined the importance of gaining a better understanding of how to ensure that patients and service users receive safe, effective and dignified care, and the need to equip those providing care with the necessary skills, knowledge and behaviours.^84^

The importance of staff being knowledgeable about the children they care for and their intellectual disability also featured in the systematic review of the experience of iatrogenic harm during hospitalisation for children with intellectual disability included within this review (referred to in objective 1 and role of family and carers findings).^15^

#### Objective 3: identify patient safety interventions, improvement initiatives, recommendations and examples of good practice

##### Supporting initiatives, recommendations and good practice

Ten articles using diverse designs (including commentary/opinion pieces, qualitative methods, service improvement, discussion/special interest/featured articles and short reports), reported either examples of initiatives to support safe care for people with learning disabilities in hospital, or recommendations to support good practice (see table 2).^85–94^ A qualitative content analysis of 60 documents mapped the content of existing hospital passports for people with intellectual disability and concluded that this approach can enhance safety and person-centred care, but acknowledged there is much variation between current hospital passports which may limit effectiveness.^89^ Six articles provided specific examples of how to enhance good practice.^85 88 91–94^ These included a commentary highlighting how hospital pharmacists can contribute to safety when supporting people with intellectual disability in hospital,^85^ a special interest/review article focusing on the presurgical needs of those with Down syndrome and how patient safety can be optimised,^88^ and an opinion piece/review presenting recommendations for the perioperative management of children with autism.^91^ Additionally, a featured article presented how simulations can educate nurses to maintain safety when caring for patients with autism spectrum disorder,^92^ and a short report highlighted the importance of: reliable identification of children with intellectual disability; exploring indirect indicators of poor quality care and consumer engagement and the voice of the child with intellectual disability.^93^ Finally, a research/discussion article explored key issues in working with people with intellectual disabilities and provided methods to improve the care provided.^94^

**Table 2:** An overview of articles relating to supporting initiatives, recommendations and good practice

Author and year	Aim and method/article type	Key findings
Blair (2013)^94^	To explore key issues in working with people with IDs and how to minimise clinical risk and ensure care is provided in an appropriate, timely and lawful manner. *Research/discussion article.*	Discussion and practice examples around the following areas: core reasonable adjustments; hospital passport; assessing a person’s capacity to consent to treatment; involving people with IDs in improving services and safety; how to improve care for people with an ID and reduce clinical risks; and reducing clinical risk improving care.
Flood (2017)^85^	Raise awareness of how hospital pharmacists can contribute to safety when supporting people with ID in hospital. *Commentary.*	To help pharmacists ensure people with IDs receive reasonably adjusted quality care it is important that; pharmacists know that a patient has IDs, pharmacy staff are aware of general healthcare and specific medication-related issues, transitions of care are considered as they are particularly vulnerable for people with IDs and people with IDs require equitable care that is appropriate for their needs.
Friese (2015)^86^	Develop care plans and an educational module for nurses caring for patients with LDs. *Improvement project.*	Key components of care plans were communication, a safe environment, enhancing patients’ behaviour and cooperation with care, and carer involvement. Nurse educational module aimed to increase understanding of needs of LD patients, improve communication and prevent adverse events. After completing the education module analysis showed significant improvement in nurses’ confidence when caring for patients with LDs.
Glasby (2002)^87^	Explore how a specialist LD team aimed to improve patient care for those with LDs. *Improvement project.*	Core tasks of LD team included: accompanying individuals to appointments, ensuring individuals understand what is going to happen in hospital, considering consent issues, liaising with wards to help them understand the person’s needs, providing practical support and advocating for the person’s needs in hospital, enabling carers to have a break, facilitating community support before discharge, following up after discharge to ensure that all needs are being met, educating acute staff and developing training materials for staff and trainees.
Lewanda (2016)^88^	Optimise patient safety for children with DS by choosing the most appropriate setting and perioperative personnel, and to mitigate those risk factors amenable to intervention. *Special interest article/review.*	Presurgical evaluations for children with DS should identify appropriate personal and equipment and focus on; combining 2+ ompatible surgical procedures under one anaesthesia event, assessing for undiagnosed or residual heart disease and the presence of pulmonary hypertension, considering potential cervical spine instability, assess if patient is taking dietary supplements and having various options available for anaesthesia during surgery.
McIntosh (2020)^92^	Address unintentional injuries (eg, medication, sharps, physical injury, diet, and overstimulation) that an individual with ASD may experience while in a healthcare environment. *Featured article.*	Simulations can educate nurses to maintain safety when caring for a patient with ASD in the professional environment. This article presents simulation ideas/activities around: medication, diet, environment, sharps, hypersensitivity, ASD routines, treatment, stimming behaviours and crisis management.
Mimmo (2020)^93^	Highlight areas that must be addressed to provide the foundation for measuring, understanding and enhancing equity in the quality of care for children with ID. *Short report.*	The report highlights the importance of: (1) reliable identification of children with ID; (2) exploring indirect indicators of poor quality care and (3) consumer engagement and the voice of the child with ID.
Northway (2017)^89^	Map the content of existing hospital passports for people with ID to inform nursing practice and future research. *Qualitative-content analysis.*	60 documents developed by provider organisations in the UK and Northern Ireland were reviewed and varied considerably in terms of length, title and content. Most frequent content included; name, level of communication (expression and understanding), level of support required with nutrition, mobility, sleeping, communication of pain and distress, behaviour, personal care, allergies, contact person. Patient and primary care information absent in some documents. Concerns it may give relatives or carers a false sense of security.
Read (2012)^90^	Identify areas of risk for patients with ID while in hospital to develop a rapid risk assessment tool for use in an acute hospital to assess immediate and potential risk, identify risk reduction actions and develop appropriate care bundles. *Improvement project.*	Implementation of the care bundles gave structure and clear evidence‐based guidance to deliver the best care for those with IDs. There was a reduction in bed days, lowering the risk of adverse events occurring, saving money in bed days and readmission penalties.
Vlassakova (2016)^91^	Summarise experiences and recommendations for the perioperative management of children with autism. *Opinion piece/review.*	Children with autism each display a unique behavioural profile. Collecting information about the patient in advance, establishing good rapport with the family, clear communication with all members of the perioperative team are key to success. Minimising perioperative stress, providing quiet environment, avoiding use of potential harmful medications assure smooth perioperative care and minimise adverse events.

ASD, autism spectrum disorder; DS, Down syndrome; ID, intellectual disability; LD, learning disability.

Three articles described improvement work.^86 87 90^ One project identified areas of risk for people with intellectual disability while in hospital, and developed and successfully implemented a rapid risk assessment tool to assess immediate and potential risk, identify risk reduction actions and develop appropriate care bundles.^90^ The second project identified core tasks of a specialist learning disability team to improve patient care for those with learning disabilities, examples included: educating acute staff, developing training materials for staff and trainees, considering consent issues and facilitating community support before discharge.^87^ A mixed-methods study comprising literature review and improvement work, developed care plans and an educational module. After completing the module, there was an increase in nurses’ confidence when caring for people with learning disabilities.^86^

Further initiatives, recommendations and good practice examples were identified in the grey literature.^95–107^ For brevity, we provide further information and signpost to these resources in online supplemental appendix 4.

## Discussion

To the authors’ knowledge, this is the first scoping review to synthesise both the academic and grey literature focusing on hospital patient safety outcomes for people with learning disabilities. While, as a narrative synthesis we are unable to state unequivocally the relationship between having learning disabilities and safety outcomes, our findings do suggest that there are multiple ways in which people with learning disabilities might experience poorer outcomes compared with people without. Our review demonstrates that there are inequalities and inequities for a range of specific patient safety outcomes including adverse events, quality of care, maternal and infant outcomes and postoperative outcomes. This disparity needs urgent attention. Nonetheless, we did identify a range of potential protective factors, such as the roles of family and carers and the extent to which health professionals are able to understand the needs of people with learning disabilities. Research has focused on developing interventions and good practice guidance, yet this is predominantly accounted for within the grey literature, meaning that robust evidence is still needed.

Some poorer outcomes are likely through the ‘direct effects’ of having a learning disability, for example, the increased incidence of comorbidities in children with learning disabilities accounted for the increased likelihood of postoperative complications in one study.^59^ However, it is also abundantly clear that there are multiple ‘indirect effects’ of having learning disabilities that may amplify problems. The review highlighted the prevailing potential risk of inadequate systems to identify and flag people with a learning disability when they enter an acute hospital setting, and the knock on effect this can have on the ability to effectively monitor patient safety incidents for these patients.^14 93^ Crucially, if patient identification and flagging and therefore patient safety incident monitoring is not fit for purpose, this creates a significant knowledge gap which greatly limits the development of much needed solutions to address patient safety issues.

Further principal issues likely to manifest in differential outcomes included problems with communication (eg, patients to staff, staff to patients, intra-agency and interagency), staff attitudes, the role of family and carers, staff awareness and knowledge/training and variation in the quality and level of healthcare received. These indirect effects fall squarely in the realm of quality and safety efforts, modifiable potentially through service redesign, increased resources, training, professional specialisation and appropriate adaptation of practice. Promising interventions and good practice examples were identified such as risk assessment tools,^90^ preoperative and perioperative management recommendations,^88 91^ hospital passports^89 94 95^ and education modules.^86^

We explore these issues through a patient safety ‘lens’, and what is perhaps most striking about our findings, is their lack of novelty. One of the earliest national reports within the UK—‘Healthcare for all’^41^—found similar issues, and made a series of recommendations. It is clear from our review that since this report, very little has changed in terms of the experience of people with learning disabilities and their families within acute care settings, either nationally or internationally. The exploration of this issue as a ‘patient safety problem*’* allows us to understand how, through the design of our healthcare system we create—and seek to solve—safety problems from the perspective of those moving through and navigating the system.

In an unrelated study, Fylan *et al* examined the medicines management system for heart failure patients discharged from hospital into the community, and developed a framework called ‘Gaps, Traps, Bridges and Props’ which may be useful when thinking about our review findings.^108^ ‘Gaps*’* occur in our systems at points of discontinuity or transition, and evidence from across patient safety literature indicates that gaps in the structure and design of services create ‘safety gaps’ that present opportunities for problems for patients, especially when care is suboptimal or fails.^109 110^ It is arguable that those patients with complex needs or specific vulnerabilities that require greater continuity of care, are more at risk when crossing these ‘safety gaps’—in effect, their vulnerability amplifies the risk of experiencing a patient safety problem. In our review, it is evident that people with learning disabilities may disproportionately suffer due to these gaps in healthcare systems. Examples of this would include poor inter-agency communication,^79^ and hospitals lacking effective systems for identifying and flagging patients.^14^ Sometimes, the design of services/organisations goes beyond creating a ‘gap*’*—which may or may not result in a safety problem for patients. ‘Traps*’* are here defined as features of system design that actively make problems more likely. An example of a ‘trap*’* from our review is the need for training on learning disabilities for healthcare staff.^49 83 84^ Without specific knowledge of, and training in caring for those with a range of learning disabilities, it is perhaps understandable that staff regularly fail to make reasonable adjustments to accommodate specific needs.^74^

This framing provides the possibility to ameliorate the issues that result, either through formalised ‘bridges*’*, or further supporting the range of informal ‘props*’* that serve to reduce problems when care is suboptimal, or fails. ‘Bridges*’* are viewed as formalised features of a system, designed to span service gaps, and support continuity of care.^108^ We found a number examples—from patient-held passports,^89 94 95^ to specialist learning disability teams.^87^ However, our review also found that these ‘bridges*’* are often inconsistently available or applied, a position that could further amplify problems if staff have come to rely on them for support when needed. The most prevalent mechanism for supporting patients with learning disabilities came through the role of families and carers. Although the need to reduce ambiguity about the role of the parent^73^ and the importance of clarifying what carer involvement includes^75^ were emphasised, we found a range of evidence that suggested families and carers regularly ‘prop*’* up services—from helping with feeding and personal care,^25^ to facilitating communication^74^ and being involved in discharge planning^6^—and that without this ‘prop*’*, the outcomes for patients with learning disabilities may well be poorer.

### Implications

Our review demonstrates the piecemeal and wide-ranging nature of the extant evidence, in terms of specific learning disabilities and outcomes of interest, and with a range of methodologies used. Therefore, we propose that research is needed to establish the burden of harm for people with learning disabilities as a result of patient safety incidents and poor quality of care, in hospital settings. This goes beyond learning from deaths—we need to understand what happens with care for people with learning disabilities more generally. Second, research needs to understand the mechanisms through which these effects might be seen. It is this approach that holds significant promise from the point of view of service improvement and redesign, as well as training and curriculum development. Put simply, we cannot change what we do not yet fully understand. Finally, attention must be given to the existing recommendations from the range of reports already published. For example, common recommendations across many previous reports include: the need for better systems to identify people with learning disabilities in healthcare settings^9 39 41 46^; the need for improved communication and information sharing between agencies and providers^9 46 47^; and the need for education and training in caring for people with learning disabilities.^11 39 41 46 47 49 83 84^ There is already a wealth of learning about the problems that exist for people with learning disabilities and their families, what is needed now is policy level action.

### Limitations

Despite an inclusive search strategy, relevant articles may not have been identified if they were not available in the sources searched. Additionally, due to the nature of the review, we did not conduct formal quality assessments and were therefore unable to make reflections and comparisons of article quality.

## Conclusion

The academic and grey literature indicates that while in hospital, people with learning disabilities might experience poorer patient safety outcomes. The involvement of family and carers, and understanding the needs of people with learning disabilities in hospital were highlighted as potential protective factors. Many promising interventions and examples of good practice exist, however, these may not be widely available or have been applied inconsistently.

## Supplementary Material

Reviewer comments

Author's manuscript

## Data Availability

No additional data available.
