# Supplementary material for: Exploring patient safety outcomes for people with learning disabilities in acute hospital settings: a scoping review
Source: BMJ Open. 2021 May 19;11(5):e047102. doi: 10.1136/bmjopen-2020-047102 (PMC8137174; doi:10.1136/bmjopen-2020-047102)
Supplement: Supplementary data [file bmjopen-2020-047102supp001.pdf]

Appendix 1 MEDLINE search terms

**Learning disability (Population)**
Learning disabilit* .mp.<br>Intellectual disabilit* .mp.<br>Statemented .mp.<br>Communication disorder*1 .mp.<br>Communication impairment*1 .mp.<br>Complex communication needs .mp.<br>Intellectual* handicap* .mp.<br>Intellectual impairment*1 .mp.<br>Learning difficult* .mp.<br>Non?speaking .mp.<br>Mental handicap .mp.<br>Asperger*1 .mp.<br>Down syndrome .mp.<br>Complex need*1 .mp.<br>Autis* .mp.<br>Dyslexi* .mp.<br>Different need*1 .mp.<br>Altered need*1 .mp.<br>Sensory impair* .mp.<br>ADHD .mp.<br>Attention deficit .mp.<br>Hyperact* .mp.<br>Global development delay .mp.<br>Mental retardation .mp.<br>Cognition disorder* .mp.<br>Learning disorder* .mp.<br>Developmental disabilit* .mp.<br>Cognitive disabilit* .mp.<br>Cognitive impairment*1 .mp.<br>Communication disabilit* .mp.
**Adverse events and patient safety (Concept)**
Adverse event*1 .mp.<br>Sentinel event*1 .mp.<br>Near miss*2 .mp.<br>Close call*1 .mp.<br>Critical outcome*1 .mp.<br>Adverse outcome*1.mp.<br>Safety event*1 .mp.<br>Never event*1 .mp.<br>Serious incident*1 .mp.<br>Untoward incident*1 .mp.<br>Clinical incident*1 .mp.<br>Incident report*1 .mp.<br>Patient safety incident*1 .mp.<br>Safety incident*1 .mp.<br>Iatrogenic disease* .mp.<br>Medical error .mp.<br>Patient safety .mp.<br>Human error* .mp.<br>((adverse or avoidable or preventable or unsafe or safet*) ADJ2 (event* or outcome* or complication* or death* or effect* or reaction* or accident* or injur*)) .mp.<br>((medica* or diagnostic or therapeautic or administration or dispensing or prescri*) ADJ2 (error* or mistake* or fault*)) .mp.<br>(patient* ADJ2 (risk* or incident* or accident* or harm*)) .mp.<br>Near miss* .mp.<br>Never event* .mp.<br>Untoward incident* .mp.<br>Serious incident* .mp.

Serious report* event* .mp.<br>Medical error*1 .mp.<br>Iatrogenic .mp.
**Acute hospital setting (Context)**
Hospital*1 .mp.<br>Acute care .mp.<br>Secondary care .mp.<br>Tertiary care unit*1 .mp.<br>Ward*1 .mp.<br>Department*1 .mp.<br>In?patient*1 .mp.<br>Out?patient*1 .mp.<br>Triage*1 .mp.<br>Intensive care .mp.<br>Critical care .mp.<br>Urgent care .mp.<br>Internal medicine .mp.<br>A&E .mp.<br>Accident and emergency .mp.<br>Emergency care .mp.<br>Emergency medicine .mp.<br>Emergency treatment .mp.<br>Emergency admission .mp.<br>Hospitali#ation .mp.<br>Ambulatory care .mp.<br>Perioperative care .mp.<br>Preoperative care .mp.<br>Hospitali#ed .mp.<br>Perioperative .mp.<br>Preoperative .mp.<br>Postoperative .mp.<br>Re?operative .mp.<br>Post?operative .mp.<br>Admission .mp.<br>Casualty .mp.<br>Discharge .mp.<br>Emergency department .mp.
