# Supplementary material for: Exploring patient safety outcomes for people with learning disabilities in acute hospital settings: a scoping review
Source: BMJ Open. 2021 May 19;11(5):e047102. doi: 10.1136/bmjopen-2020-047102 (PMC8137174; doi:10.1136/bmjopen-2020-047102)
Supplement: Supplementary data [file bmjopen-2020-047102supp002.pdf]

Appendix 2 Grey literature publicly available material searched

Mencap, NHS England, NHS Improvement, Healthcare Quality Improvement Partnership (HQIP), Healthcare Safety Investigation Branch (HSIB), The Kings Fund, Agency for Healthcare Research and Quality (AHRQ), Care Quality Commission (CQC), The Health Foundation, World Health Organisation, Institute of Healthcare improvement, Collaborations for Leadership in Applied Health Research and Care (CLAHRC), Nuffield Trust, Public Health England, The Office for National Statistics, The National Institute for Clinical Excellence, Mind, Learning Disability England, the British Institute of Learning Disabilities (BILD), Learning Disability Practice – RCNi, The National Research Center on Learning Disabilities (NRCLD), Royal College of Nursing, Royal College of Midwives, Nursing & Midwifery Council, Faculty of Dental Surgery, Faculty of Intensive Care Medicine,
Royal College of Anaesthetists, Royal College of Emergency Medicine, Royal College of Obstetricians and Gynaecologists, Royal College of Paediatrics and Child Health, Royal College of Physicians of Edinburgh, Royal College of Physicians of Ireland, Royal College of Physicians of London, Royal College of Physicians and Surgeons of Glasgow, Royal College of Surgeons in Ireland, Royal College of Surgeons of Edinburgh, Royal College of Surgeons of England.
