# Supplementary material for: Exploring patient safety outcomes for people with learning disabilities in acute hospital settings: a scoping review
Source: BMJ Open. 2021 May 19;11(5):e047102. doi: 10.1136/bmjopen-2020-047102 (PMC8137174; doi:10.1136/bmjopen-2020-047102)
Supplement: Supplementary data [file bmjopen-2020-047102supp003.pdf]

Appendix 3 Included articles with author, year, country, aims, participants, methodology and key findings (presented in alphabetical order)

Author	Year	Country	Aims	Participants	Methodology	Key findings
Bartz-Kurycki et al[59]	2018	USA	Investigate whether DS is a risk factor for postoperative complications in paediatric patients undergoing gastrointestinal and non-cardiac thoracic surgery, and determine factors associated with complications	Total: 91,478 patients <18y old who underwent gastrointestinal or non-cardiac thoracic surgery. With DS: 1,476 (1.6%)	Quantitative: Retrospective cohort study using univariate analysis and multivariate logistic regression	DS patients had significantly higher postoperative complication rates than controls. However, comorbidities rather than DS were a greater risk factor for complications
Best, Asaro and Curley[33]	2019	USA	To compare current analgesia and sedation management practices between critically ill children with pre-existing cognitive impairment and critically ill neurotypical children, including possible indicators of therapeutic efficacy	Total: 2,449 patients 2 weeks – 17y old were included who underwent invasive mechanical ventilation for acute airway and/or parenchymal lung disease. With CI: 412	Quantitative: Secondary analysis of prospective data using linear, cumulative logit, logistic, multinomial logistic, proportional hazards, and Poisson regression	CI patients received significantly lower doses of analgesia and sedation medication than those without CI. However, it was unclear if this was due to lower requirements or vulnerabilities to inadequate assessment
Blair[94]	2013	UK	To explore key issues in working with people with IDs and how to minimise clinical risk and ensure care is provided in an appropriate, timely and lawful manner	A guide about how to get things right for people with IDs with examples from practice	Research /discussion article	Discussion and practice examples around the following areas: core reasonable adjustments; hospital passport; assessing a person’s capacity to consent to treatment; involving people with IDs in improving services and safety; how to improve care for people with an ID and reduce clinical risks; and reducing clinical risk improving care

Author	Year	Country	Aims	Participants	Methodology	Key findings
Boylan et al[60]	2016	USA	Assess and compare short-term outcomes of total hip arthroplasty in patients with and without DS	Total: 543,085 patients who underwent total hip arthroplasty in 1998 – 2010. With DS: 241 (0.04%)	Quantitative: Retrospective cohort study using logistic regression modelling, independent sample t-tests and linear regression	DS patients had significantly increased rates of medical, surgical and any complication compared to matched controls. This included pneumonia, urinary tract infection and wound haemorrhage. Patients with DS also had a longer mean length of stay
Bradbury-Jones et al[77]	2013	UK	Review evidence regarding the promotion of health, safety and welfare of adults with LD in acute care	Studies focussed on adults with LDs	Literature review	Six areas of influence on the health, safety and welfare of adults with LDs in acute hospitals were identified: care provision; communication; staff attitudes; staff knowledge; supporters and carers; and physical environment
Brittle[78]	2004	UK	Consider how nurses may deal with increasing numbers of LD patients accessing generic health services including hospitals	Studies including people with LDs accessing generic health services	Discussion article	To ensure that nurses do as much as possible to recognise risk when caring for people with LDs, they must; recognise any prejudices and overcome them, acknowledge that people with LDs have the same rights to healthcare as others, develop further understanding of LDs and collaborate with carers and professionals
Brown et al[53]	2016	Canada	Compare the occurrence of labour induction, c-section and operative vaginal delivery in women with and without IDD and determine whether pre-pregnancy health conditions or pregnancy complications explain any differences	Total: 386,706 deliveries to 263,284 women. With IDD: 3,932 deliveries to 2,584 women Without IDD: 382,774 deliveries to 260,700 women	Quantitative: Retrospective cohort study using Poisson regression and mediation analysis	Women with IDD were younger, lived in lower income areas and had higher rates of pre-pregnancy health conditions including: pre-existing diabetes mellitus, herpes or HIV, epilepsy, and psychiatric disorders. They had higher rates of complications such as pre-eclampsia and fetal death, and were more likely to have labour interventions such as induction and caesarean. Psychiatric disorders were the most important variable for labour induction and caesarean

Author	Year	Country	Aims	Participants	Methodology	Key findings
Cairo et al[68]	2019	USA	To measure medical and surgical complications, as well as in-patient mortality, in pediatric patients with DS undergoing intestinal operations and to compare these to patients without DS	Total: 17,026 pediatric patients undergoing intestinal operations.<br>With DS: 444 (2.6%)	Quantitative: Cohort study using multivariable regression model	Mortality and medical complications were significantly lower for patients with DS. Surgical complications were not significantly different between patients with and without DS
Evans et al[61]	2014	USA	Identify differences in in-hospital mortality after cardiac surgery in paediatric patients with and without DS	Total: 51,309 patients <18y old who underwent surgery to correct congenital heart disease<br>With DS: 4231 (8.2%)	Quantitative: Retrospective cohort study using multivariable logistic regression analysis	When controlling for risk category, premature birth, presence of ≥1 major non-cardiac structural anomalies, and age, a diagnosis of DS was associated with a lower odds of in-hospital death
Flood[85]	2017	UK	Raise awareness of how hospital pharmacists can contribute to safety when supporting people with ID in hospital	Discussing studies and policies focussing on people with ID in hospital	Commentary	To help pharmacists ensure people with IDs receive reasonably adjusted quality care it is important that; pharmacists know that a patient has IDs, pharmacy staff are aware of general healthcare and specific medication-related issues, transitions of care are considered as they are particularly vulnerable for people with IDs and people with IDs require equitable care that is appropriate for their needs

Author	Year	Country	Aims	Participants	Methodology	Key findings
Friese and Ailey[86]	2015	USA	Develop care plans and an educational module for nurses caring for patients with LDs	Nurses completed a pre-evaluation (n = 75) and post-evaluation (n = 99) questionnaire. Over 300 nurses completed the educational module	Mixed methods comprising: Literature review and PDSA cycles to develop care plans and educational module. Quantitative assessment of nurses' confidence in caring for patients with LD after completing the module using chi squared	Key components of care plans were communication, a safe environment, enhancing patients' behaviour and cooperation with care, and carer involvement. Nurse educational module aimed to increase understanding of needs of LD patients, improve communication and prevent adverse events. After completing the education module analysis showed significant improvement in nurses' confidence when caring for patients with LDs
Glasby[87]	2002	UK	Explore how a specialist LD team aimed to improve patient care for those with LDs	A specialist learning disability team of nursing and support staff set up to improve care for people with LDs	Improvement project: Observational	Core tasks of LD team included: accompanying individuals to appointments, ensuring individuals understand what is going to happen in hospital, considering consent issues, liaising with wards to help them understand the person's needs, providing practical support and advocating for the person's needs in hospital, enabling carers to have a break, facilitating community support before discharge, following up after discharge to ensure that all needs are being met, educating acute staff and developing training materials for staff and trainees

Author	Year	Country	Aims	Participants	Methodology	Key findings
Graber et al[69]	2021	USA	To determine the incidence of difficult intubation and perioperative respiratory adverse events in pediatric patients with trisomy 21 receiving general anesthesia compared to a matched control population	Total: 2649 patients. Trisomy 21 group: 1213 (47.8%)	Quantitative: Retrospective cohort study, chart review using logistic regression analysis	Patients with trisomy 21 experienced more perioperative respiratory adverse events compared to well-matched controls, largely attributable to the increased frequency of obstructed ventilation in trisomy 21 patients
Hemsley and Balandin[79]	2014	Australia	To synthesise the findings of research into communication in hospital for people with severe communication disabilities arising from lifelong and acquired stable conditions including cerebral palsy, autism, intellectual disability, aphasia following stroke, but excluding progressive conditions and those solely related to sensory impairments of hearing or vision	Studies including people with severe communication disabilities	Literature review – metasynthesis	The reviewed highlighted strategies to improve communication. 1) Develop services, systems and policies that support improved communication; 2) Devote enough time to communication; 3) Ensure adequate access to communication; 4) Access personally held written information; 5) Collaborate effectively with carers, spouses, and parents; and 6) Increase communicative competence of staff

Author	Year	Country	Aims	Participants	Methodology	Key findings
Hemsley et al[34]	2016	Australia	Identify research reports regarding investigating the care or safety of adults with communication disabilities in hospital, and to analyse findings according to the generic model of patient safety	Studies including hospital patients with communication disabilities	Literature review	Patient safety incident and adverse event reporting lacked detail e.g. little demographic, descriptive, temporal and categorical information about the patient and staff and how events were detected. Successful advocacy affected outcomes, although where advocacy was ignored outcomes were worse. Stories of adverse events themes included; suffering, isolation due to not having a method to communicate with nurses, a perilous care situation culminating in an adverse event and protective carers discovering or forestalling an adverse event
Huang et al[70]	2020	Taiwan	Evaluate outcomes after major surgery in children and adolescents with ID	Total: 21,730 patients.<br>With ID: 2173 (10%)	Quantitative: Nested cohort study using multiple logistic regression models	Children with ID had a higher risk of postoperative pneumonia, sepsis, and 30-day mortality compared with children without ID. Children with ID had longer lengths of hospital stay when compared with children with no ID
Iacono et al[25]	2014	Australia	Evaluate the evidence regarding how hospital systems respond to adults with ID, their families and carers	Studies focussed on people with ID or carers or staff perceptions and experiences of quality of care of people with ID	Systematic review and meta-synthesis	Themes included: individual fear of hospital encounters; failure of staff to provide care; staff knowledge and skills; staff attitudes; staff or system failures to adjust to needs; over reliance on paid and family carers for basic needs and advocacy; and enhancers to appropriate hospital care
Jolly[80]	2015	USA	Familiarise the paediatric nurse with autism and create a resource for successful inpatient treatment of a child with the disorder	Discussing knowledge of working with children with autism	Discussion article	Themes included; understanding autism, encouraging family involvement, best way of communicating, change is a challenge for children with autism, consistent caregivers, safe environment, emotional triggers and reward systems, and MDT from admission

Author	Year	Country	Aims	Participants	Methodology	Key findings
Kelly et al[36]	2015	UK	Compare 30-day hospital readmission rates of people with and without LDs	Total: 66,870 patients. With LDs: 256 (0.38%)	Quantitative: Retrospective audit using chi-squared	No significant difference in 30-day readmission rates for patients with and without LDs. However, 69% of readmissions of those with LDs were potentially preventable. Those with more profound LDs were at greater risk of experiencing poor quality care and experiencing readmission within 30 days, and this group comprised over half of the PPRs
Kopecky et al[81]	2013	USA	Characterise the needs of hospital patients with autism in various categories via a survey to aid facilitating the inpatient experience and improve quality of care	80 parents and guardians of patients with autism	Mixed methods: Survey comprising qualitative and quantitative items	Those with autism have a wide range of challenges and needs including communication, social/pragmatic concerns, and sensory processing whilst in hospital. An individualised approach must be used to assess and accommodate needs. 50% of parents had safety concerns for their child's hospital stay including; wandering or elopement, self-injury or aggression, pica, and pulling out IV tubing
Lewanda et al[88]	2016	USA	Optimise patient safety for children with DS by choosing the most appropriate setting and perioperative personnel, and to mitigate those risk factors amenable to intervention	Specialist team presenting their knowledge on working with children with DS	Special interest article/review	Pre-surgical evaluations for children with DS should identify appropriate personal and equipment and focus on; combining 2+ compatible surgical procedures under one anaesthesia event, assessing for undiagnosed or residual heart disease and the presence of pulmonary hypertension, considering potential cervical spine instability, assess if patient is taking dietary supplements and having various options available for anaesthesia during surgery

Author	Year	Country	Aims	Participants	Methodology	Key findings
Lin et al[62]	2011	Taiwan	Clarify whether ID is an independent risk factor for in-hospital major surgeries, and to validate the postoperative adverse outcomes in patients with ID	Total: 3,983 patients with preoperative diagnosis of ID who underwent inpatient major surgeries in 2004 - 2007	Quantitative: Retrospective, (matched controls) study using descriptive statistics and multivariate logistic regression	Surgical patients with ID had significantly higher incidence of preoperative comorbidities and postoperative complications while consuming more medical resources than controls. They also had higher average length of hospital stay and use of intensive care, as well as higher rates of acute renal failure, pneumonia, postoperative bleeding septicaemia, stroke and any complications. Postoperative 30-day mortality showed no significant difference between patients with or without ID after adjusting for teaching hospital, low income, urbanization and coexisting disease
McConnell, Mayes and Llewellyn[56]	2008	Australia	Explore the prevalence of poor pregnancy and birth outcomes in women with ID and/or self-reported learning difficulties in an antenatal population	Total: 834 women who gave birth to 839 children. With ID and/or self-reported learning difficulties: 54 Without ID and/or self-reported learning difficulties: 780	Quantitative: Cohort study using descriptive statistics, odds ratios with confidence intervals	Women with ID and/or self-reported learning difficulties had significantly higher prevalence of pre-eclampsia and a higher rate of low birthweight. They also had higher rates of pre-term delivery and high birth weight, but these differences were not statistically significant
McIntosh et al[92]	2020	USA	Address unintentional injuries (e.g. medication, sharps, physical injury, diet, and overstimulation) that an individual with ASD may experience while in a health care environment	Presenting simulation ideas/activities	Featured article	Simulations can educate nurses to maintain safety when caring for a patient with ASD in the professional environment. This article presents simulation ideas/activities around: medication, diet, environment, sharps, hypersensitivity, ASD routines, treatment, stimming behaviours, and crisis management

Author	Year	Country	Aims	Participants	Methodology	Key findings
Mimmo et al[93]	2020	Australia	Highlight areas that must be addressed to provide the foundation for measuring, understanding and enhancing equity in the quality of care for children with ID	Presents areas to be addressed based on a larger programme of work	Short report	The report highlights the importance of: 1) reliable identification of children with ID; 2) exploring indirect indicators of poor quality care; and 3) consumer engagement and the voice of the child with ID
Mimmo et al[73]	2019	Australia	Identify evidence regarding the parental experience of hospitalisation with a child with ID and care quality and safety	Studies focussed on parents or carers of children who are inpatients with ID	Literature review: scoping review and meta-synthesis	Findings from 11 studies were consolidated into five themes; being more than a parent, importance of role negotiation to reduce ambiguity about the role of the parent, building trust and relationships through effective communication, the cumulative effect of previous experiences of hospitalisation and healthcare staff taking time to know the child as an individual. Partnerships in care are vital to deliver safe care for children with ID
Mimmo, Harrison and Hinchcliff[15]	2018	Australia	Narratively synthesise evidence concerning the experience of iatrogenic harm during hospitalisation for children with ID	Studies focussed on child inpatients with ID	Systematic review and narrative synthesis	16 papers provided evidence around: the assumptions of HCWs; reliance on parental presence; and the need for HCWs to understand the IDs experienced by children in their care. There are specific aspects of hospitalisation that expose children with ID to harms that are preventable, avoidable and not experienced to the same extent by children without ID

Author	Year	Country	Aims	Participants	Methodology	Key findings
Mitra et al[54]	2018	USA	Compare the risk of postpartum hospital admission and emergency department visits during the first postpartum year among women with and without IDD	Total: 779,513 deliveries by women who gave birth in 2002 - 2012.<br>Mothers with IDD: 1,104<br>Mothers without IDD: 778,409	Quantitative: Retrospective cohort study using chi-square, t-tests and Wilcoxon rank-sum tests	Women with IDD had higher prevalence rates for hospital admission and emergency department visits during all critical postpartum periods than those without IDD, with at least 2x higher rates for any hospitalisations within 1–42, 43–90, and 91–365 days after childbirth. Women with IDD also had a higher risk for repeated hospitalisations
Northway et al[89]	2017	UK	Map the content of existing hospital passports for people with ID to inform nursing practice and future research	Review of 60 hospital passports	Qualitative: content analysis.	60 documents developed by provider organisations in the UK and Northern Ireland were reviewed and varied considerably in terms of length, title and content. Most frequent content included; Name, Level of communication (expression and understanding), Level of support required with nutrition, Mobility, Sleeping, Communication of pain and distress, Behaviour, Personal care, Allergies, Contact person. Patient and primary care information absent in some documents. Concerns it may give relatives or carers a false sense of security
Parish et al[55]	2015	USA	Explore and compare both the pregnancy outcomes of women with and without IDD, and health outcomes of children born to mothers with and without IDD	Total: 3,859,539 pregnant women.<br>Women with IDD: 1,706<br>Women without IDD: 3,857,833	Quantitative: Retrospective study using chi-square and logistic regressions	Women with IDD were more likely to have a c-section and a longer hospital stay. They were also more likely to experience adverse pregnancy outcomes such as preeclampsia, preterm birth, early labour and their infants were more likely to have a low birth weight

Author	Year	Country	Aims	Participants	Methodology	Key findings
Poulton, Armstrong and Nanan[57]	2018	Australia	Investigate the impact of ADHD and the effect of stimulant medication on women's perinatal outcomes	5,056 women treated with stimulants for ADHD<br>25,249 untreated women	Quantitative: Cohort study, multiple logistic regression	Women treated for ADHD with stimulants at any time (before, before and during, or only after the index pregnancy) had lower rates of spontaneous labour, and higher rates of caesarean delivery, active new-born resuscitation, and neonatal admission. 4 h. stimulant treatment for ADHD before or before and during pregnancy was also associated with higher rates of preeclampsia, preterm birth, and low 1-min Apgar score
Printz et al[71]	2019	USA	Examine whether outcomes differ between pediatric patients with and without ASD in a national cohort of children undergoing tonsillectomy	Total: 27,040 patients.<br>With ASD: 322 (1.2%)	Quantitative Retrospective cohort study using chi-square, t-tests, logistic regression, and generalized linear regression	After controlling for potential confounders, multivariable modeling suggested patients with ASD had a shorter length of stay and were less likely to experience complications
Purifoy et al[63]	2019	USA	Determine whether DS is associated with higher mortality, longer length of stay and greater incidence of gastrostomy and/or tracheostomy after complete repair of tetralogy of Fallot	Total: 4790 patients aged 1 day - 19y old<br>With DS 430 (8.9%)	Quantitative: Retrospective cohort study using chi-square	Patients with DS had longer mean postoperative length of stay after complete repair and greater incidence of postoperative gastrostomy than those without. However, no significant difference in mortality prior to discharge or rates of postoperative tracheostomy

Author	Year	Country	Aims	Participants	Methodology	Key findings
Read, Johnson and Tristan[90]	2012	UK	Identify areas of risk for patients with ID whilst in hospital to develop a rapid risk assessment tool for use in an acute hospital to assess immediate and potential risk, identify risk reduction actions and develop appropriate care bundles	Total: 54 rapid risk assessments with people with IDs admitted to the pilot wards	Improvement project: PDSA cycles	Implementation of the care bundles gave structure and clear evidence-based guidance to deliver the best care for those with IDs. There was a reduction in bed days, lowering the risk of adverse events occurring, saving money in bed days and readmission penalties
Redley et al[74]	2019	UK	Understand the views of qualified medical practitioners regarding reasonable adjustments and the quality of the care and treatment provided to adult inpatients with ID	Total: 14 medical practitioners	Qualitative: Interview study using thematic analysis	Medical practitioners focused on two accounts: the patients' communication difficulties and vulnerability to behaviours that did not conform to a hospital's expectations, and their biomedical complexities. They reported making limited use of “reasonable adjustments" and turned to caregivers to facilitate communication and manage behaviours likely to upset hospital routines
Shah et al[37]	2009	Canada	Review outcomes and toxicity of chemotherapy for acute lymphoblastic leukaemia in children with DS	30 patients with DS<br><br>60 patients without DS	Quantitative: Cohort study using Cox proportional hazards and a matched generalized linear model	Patients with DS spent more days in hospital, particularly during the induction phase of treatment

Author	Year	Country	Aims	Participants	Methodology	Key findings
Sheehan et al[6]	2016	UK	Examine steps that hospitals are taking to deliver high-quality care to people with a LD, and examine any impact these have on care quality	Total: 176 patients case note audit from adults with LDs who received inpatient hospital care in acute general and mental health services in May 2013 - April 2014	Quantitative: Clinical case note audit study using multivariable logistic regression	The strongest performance was in ensuring that family or carers were involved in discharge planning (84% evidencing this was enacted). Weight measurement or BMI was recorded in 58% notes. Compliance with all other audit criteria was <50%. Records of swallowing assessments, epilepsy risk assessment (for those with epilepsy) and that a health passport was used fared particularly badly, with evidence of these interventions in only 19%, 21% and 24% cases, respectively. For most quality indicators, there was a non-statistically significant trend for improved performance in services with a LD liaison nurse. The presence of an electronic flagging system showed less evidence of benefit
St Louis et al[65]	2014	USA	Descriptively analyse surgical outcomes from repair of complete atrioventricular septal defect in infants	Total: 2,399 patients who underwent surgery in 2008 - 2011.<br>With DS: 78.4%	Quantitative: Cohort study using Wilcoxon rank sum tests	Mortality and major complication rates were lower for patients with DS than for those without. Length of stay was similar
Toth et al[64]	2013	Hungary	Compare postoperative morbidity and mortality of paediatric patients with and without DS who underwent heart surgery	Patients <18y old who underwent heart surgery and were admitted to the cardiac ICU in 2003 - Dec 2008.<br>With DS: 129<br>Without DS: 1667<br><br>After propensity matching<br>With DS: 111<br>Without DS: 111	Quantitative: Retrospective cohort study using χ2-test, Fisher's exact test and t-tests using a non-parsimonious multivariable logistic regression model	After propensity matching, there was no significant variation between the groups regarding rates of postoperative complications or mortality

Author	Year	Country	Aims	Participants	Methodology	Key findings
Tuffrey-Wijne et al[75]	2016	UK	Identify factors that affect carer involvement for people with ID in acute hospitals and develop guidance to promote effective carer involvement	Survey response from 990 staff and 88 carers. Interviews with 68 hospital staff and 37 carers	Mixed methods	High staff awareness of good practice. Carers generally satisfied with how they were treated. A significant minority were dissatisfied on factors including expectations to provide basic nursing care, their expertise not being acted upon and discrepancies in perspective on the role of carers. A new model for clarifying carer involvement includes; the degree carers are 'workers' contributing to basic nursing care the degree carers are experts or non-experts
Vlassakova and Emmanouil[91]	2016	USA	Summarise experiences and recommendations for the perioperative management of children with autism	Studies focussed on children with autism	Opinion piece/review	Children with autism each display a unique behavioural profile. Collecting information about the patient in advance, establishing good rapport with the family, clear communication with all members of the perioperative team are key to success. Minimising perioperative stress, providing quiet environment, avoiding use of potential harmful medications assure smooth perioperative care and minimise adverse events
Wilkinson[82]	2018	UK	Produce a case study detailing the plan of care for an 18 year old male patient with a moderate level of LD who was scheduled for a tonsillectomy in hospital	Focussing on an 18 year old male patient with LD	Case study	Through collaboration and effective communication between practitioners, anaesthetists, surgeon and recovery care staff, the patient was admitted for his procedure with full knowledge of his individual needs and concerns, despite his limited communication skills. The NHS passport was a valuable document in the practitioners' toolbox, offering great merit in its holistic approach to patient care

Author	Year	Country	Aims	Participants	Methodology	Key findings
Oulton et al[38]	2018	UK	Compare and identify factors that facilitate and prevent children and young people with and without LDs and long term conditions from receiving equal access to high quality hospital care and services	Interviews: 65 staff in senior clinical or managerial roles or those employed specifically to work with children and young people with LDs. Survey: 2,261 clinical and non-clinical staff with contact with children and young people and their families	Mixed methods, framework analysis	Two key themes; national variation and staff uncertainty. Lack of knowledge about policies, systems and practices at an organisational level to support care of children and young people with LDs. Considerable variation between hospitals ranging from those appearing to have few or no systems, policies or practices in place specifically for this group, with partial systems, policies or practices in place and those with a cohesive and comprehensive level of provision. There was a lack of standardised systems in place for communicating that an individual has a LD. Also a distinct lack of systems in place for recording that an individual involved in a complaint or the subject of clinical incident has a LD
Pugely et al[66]	2014	USA	Analyse the incidence of, and risk factors for, short-term complications after paediatric deformity spinal surgery	Total: 2,005 elective cases for deformity spinal surgery in patients <19y old	Quantitative: Retrospective review of a prospective cohort using univariate and multivariate analyses	In univariate analyses developmental delay was identified as a risk factor for complications after surgery, but was not associated with mortality

Author	Year	Country	Aims	Participants	Methodology	Key findings
Vervloessem et al[67]	2009	Netherlands	Compare complication rates with other centres and identify risk factors for major complications related to percutaneous endoscopic gastrostomy placement in children	Total: 467 paediatric patients undergoing percutaneous endoscopic gastrostomy placement at the hospital	Quantitative: Retrospective study using univariate and multivariate analyses	When adjusted for year and ventriculoperitoneal shunt ID was not a significant risk factor for complications

Notes. ADHD = Attention Deficit Hyperactivity Disorder, ASD = Autism Spectrum Disorder, BMI = Body Mass Index, CI = Cognitive Impairment, c-section = caesarean section, DS = Downs Syndrome, HCWs = Healthcare Workers, ICU = Intensive Care Unit, ID = Intellectual Disability, IDs = Intellectual Disabilities, IDD = Intellectual and Developmental Disabilities, IV = Intravenous Tubing, LD = Learning Disability, LDs = Learning Disabilities, MDT = multidisciplinary team, PDSA = Plan Do Study Act, PPRs = Potentially Preventable Readmissions.
