# Supplementary material for: Exploring patient safety outcomes for people with learning disabilities in acute hospital settings: a scoping review
Source: BMJ Open. 2021 May 19;11(5):e047102. doi: 10.1136/bmjopen-2020-047102 (PMC8137174; doi:10.1136/bmjopen-2020-047102)
Supplement: Supplementary data [file bmjopen-2020-047102supp004.pdf]

BMJ Publishing Group Limited (BMJ) disclaims all liability and responsibility arising from any reliance placed on this supplemental material which has been supplied by the author(s)

Appendix 4 Included grey literature material with author, webpage, year, title, aims and key information (presented in ascending date order)

Author	Webpage	Year	Title	Aims	Key information
Mencap[39]	https://www.mencap.org.uk/sites/default/files/2016-08/treat_me_right.pdf	2004	Treat me right! Better healthcare for people with a learning disability	To highlight that despite many policy reports (starting in 1992) there have been few changes in health care delivery for people with learning disabilities	Use of case studies to illustrate need for change and recommendations for change:<br>- Training for health professionals that should involve people with a learning disability.<br>- All NHS organisations must fully comply with the DDA to provide equal access to healthcare<br>- Healthcare services must address the problem of health inequalities<br>- Hospitals must fulfil their legal duty of care and provide appropriate levels of support to patients who have a learning disability<br>- There must be an inquiry into the premature deaths of people with a learning disability
Mencap[40]	https://www.mencap.org.uk/sites/default/files/2016-06/DBIreport.pdf	2007	Death by indifference	To understand the causes of deaths for people with learning disabilities in hospital	The report presents six case studies. The report highlights that the underlying cause of death for many people with a learning disability who die in hospital is the widespread ignorance and indifference throughout our healthcare services towards people with a learning disability, and their families and carers
Allyson Kent[95]	https://www.nursingtimes.net/roles/learning-disability-nurses/improving-acute-care-of-people-with-learning-disabilities-05-02-2008/	2008	Improving acute care of people with learning disabilities	To report on the development of a patient passport system for use by people with learning disabilities entering acute care settings	The patient passport is a simple tool that articulates people's individual needs and seeks to bridge the communication gap in acute care. The patient passport has evolved as a result of listening to the needs of people with learning disabilities, parents and carers and acute staff

Author	Webpage	Year	Title	Aims	Key information
Elizabeth Piper[76]	https://hydra.hull.ac.uk/assets/hull:5754a/content	2008	Investigating the experiences of people with learning disabilities in Accident & Emergency from a carer perspective	To investigate emergency healthcare - as delivered via Accident & Emergency - from the perspective of the carers of people with learning disabilities	Interview findings include the relationship staff had with both service users and carers was considered to be fundamental to a high quality service. Themes identified included Interactions that are valuing, emotional responsiveness, support, compliance and responsibilities
Sir Jonathan Michael and the Independent Inquiry into Access to Healthcare for People with Learning Disabilities[41]	https://webarchive.nationalarchives.gov.uk/20130105064250/http://www.dh.gov.uk/en/Publicationsandstatistics/Publications/PublicationsPolicyAndGuidance/DH_099255	2008	Healthcare for all: report of the independent inquiry into access to healthcare for people with learning disabilities	To review research and evidence, and the views of witnesses and stakeholders to understand the safety of care for people with learning disabilities	The report highlighted that there are risks within the care system for people with learning disabilities and these risks are exacerbated by that lack of ‘reasonable adjustments’ to services. A large number of avoidable deaths were also found. The report makes recommendations for changes within the healthcare system and gives examples of how to implement these
The Parliamentary and Health Service Ombudsman[42]	https://assets.publishing.service.gov.uk/government/uploads/system/uploads/attachment_data/file/250750/0203.pdf	2009	Six lives: the provision of public services to people with learning disabilities	To illustrate some significant failures in service across both health and social care for people with learning disabilities	Six investigation reports into deaths of people with learning disabilities following referral to the Ombudsman when complaints had not been satisfactorily answered by care providers;<br>The areas of concern included:<br>- Communication<br>- Partnership working and co-ordination<br>- Relationships with families and carers<br>- Failure to follow routine procedures<br>- Quality of management<br>- Advocacy<br>The case studies are powerful reminders of how things can go wrong, some with examples of unsafe care and adverse events in care delivered by NHS acute Trusts

**Author**	**Webpage**	**Year**	**Title**	**Aims**	**Key information**
Royal College of Nursing[96]	http://oxleas.nhs.uk/site-media/cms-downloads/RCN_Dignity_in_healthcare.pdf	2009	Dignity in health care for people with learning disabilities	To provide a resource to support nursing staff to improve dignity in health care for people with learning disabilities	Examples of good practice that makes care safer for people with learning disabilities including staff training and accessibility of care
Guidelines and Audit Implementation Network[97]	https://rqia.org.uk/RQIA/files/81/81662c46-b7bb-43a5-9496-a7f2d919c2a3.pdf	2010	Guidelines on caring for people with a learning disability in general hospital settings	To develop guidelines for care delivery to enhance safe and effective care throughout the journey within the general hospital setting for people with a learning disability	The guidelines, developed by a range of health professionals, support staff to provide safe and effective care for people with a learning disability. Guidance is given for each stage of hospital journey and include communication, attitudes, values and training for staff
The Hillingdon Hospitals NHS Foundation Trust[98]	https://www.thh.nhs.uk/about/safety/learning_dis.php<br><br>https://www.thh.nhs.uk/documents/_Patients/Guidelines_patients_learning_disabilities_Dec2011.pdf	Good Practice Guidelines - 2012	Responding to the needs of people with learning disabilities	To provide an equal service for people with learning disabilities	Examples of good practice from one Trust to improve safety and experience during hospital admission for patients with learning disabilities

Author	Webpage	Year	Title	Aims	Key information
The Confidential Inquiry into premature deaths of people with learning disabilities team[9]	https://www.hqip.org.uk/resource/confidential-enquiry-into-deaths-of-people-with-learning-disabilities-cipold-2013/#.XkP6jy7Fly4	2013	Confidential enquiry into deaths of people with Learning Disabilities	To investigate and compare the sequence of events leading to known deaths of 247 people, comprising people with learning disabilities and comparator cases from five Primary Care Trust areas of South West England, to assess avoidable or premature deaths	Most (96%) were of white UK ethnicity and 22% were under the age of 50 when they died. The median age of death for people with learning disabilities (65y for men; 63y for women) was significantly less than for the UK population (78y for men and 83y for women). Men with learning disabilities died, on average, 13 years sooner than men in the general population, and women with learning disabilities died 20 years sooner than women in the general population. The study revealed that the quality and effectiveness of health and social care given to people with learning disabilities was deficient in a number of ways
Agency for Healthcare Research and Quality[99]	https://www.innovations.ahrq.gov/profiles/comprehensive-program-support-patients-and-staff-improves-hospital-experience-adult	2013	Service Delivery Innovation Profile Comprehensive program to support patients and staff improves hospital experience for adult patients with intellectual and developmental disabilities	To develop a comprehensive set of strategies for patients and staff designed to improve the hospital experience for adult patients with intellectual and developmental disabilities	The program provides pre-hospitalisation tours, a telephone "helpline" to request special accommodations, procedure-specific informational booklets, participation in a buddy program, and a collection of multisensory materials for relaxation and distraction. It has been reported to improve patient experience

Author	Webpage	Year	Title	Aims	Key information
Department of Health[43]	https://assets.publishing.service.gov.uk/government/uploads/system/uploads/attachment_data/file/212292/Six_lives_2nd_Progress_Report_on_Healthcare_for_People_with_Learning_Disabilities_-_full_report.pdf	2013	Six Lives: Progress Report on Healthcare for People with Learning Disabilities	To report progress in responding to the Ombudsmen's recommendations in 2010 following the 'Six Lives' report which investigated the deaths of six people with learning disabilities, first highlighted by Mencap	Progress on issues which were of particular concern to people with learning disabilities and family carers in 2010 are reported. Issues included capacity and consent; staff understanding (including communications, information and reasonable adjustments); and complaints and advocacy
Mencap[44]	https://www.mencap.org.uk/get-involved/campaign-mencap/hear-my-voice/hear-my-voice-healthcare<br><br>https://www.youtube.com/watch?v=JZA9cClHqWA&feature=emb_logo	2014	Hear my voice: healthcare<br><br>Jayne and Jonathan's story about their brother Paul	NHS to take action to stop 1,200 preventable annual deaths of people with a learning disability in hospital by; everyone with a learning disability getting a quality annual health check, a health plan, and offered a hospital passport. GPs, doctors and nurses to also have training on reasonable adjustments they need to make to give quality care to people with a learning disability	Jayne and Jonathan shared the story of their brother Paul, who spent three weeks in intensive care before being prematurely transferred to a general ward where he experienced poor quality care, and later died. This involved; family members concerns of deterioration being disregarded, missing notes, prescribing drugs which worsened his condition and misinterpreting symptoms to be due to the learning disabilities. As a result the family initiated the campaign

Author	Webpage	Year	Title	Aims	Key information
Department of Health[100]	https://assets.publishing.service.gov.uk/government/uploads/system/uploads/attachment_data/file/309153/Strengthening_the_commitment_one_year_on_published.pdf	2014	Strengthening the Commitment: One year on: Progress report on the UK Modernising Learning Disabilities Nursing Review	To report the progress made in ensuring people with learning disabilities of all ages have access to expert learning disabilities nursing	Progress made in strengthening capacity, capability, quality and the profession are reported using recommendations and positive practice
1000 Lives Improvement, which is part of Public Health Wales[101]	http://www.1000livesplus.wales.nhs.uk/sitesplus/documents/1011/How%20to%20%2822%29%20Learning%20Disabilites%20Care%20Bundle%20web.pdf	2014	Improving general hospital care of patients who have a learning disability	To enable healthcare organisations and their teams to successfully implement a series of interventions to improve the safety and quality of care that patients with learning disabilities receive	An improvement guide that describes a care bundle of interventions and driver diagram. The guide also includes details of specific interventions in the appendices

Author	Webpage	Year	Title	Aims	Key information
Public Health England[102]	http://cdn.basw.co.uk/upload/basw_14709-8.pdf	2015	Working together 2: Easy steps to improve support for people with learning disabilities in hospital	An update of the Working together guide published in 2008 to help hospital staff, family members and paid support staff work jointly before, during and towards the end of any hospital admission (unplanned or planned) so an individual with learning disabilities could get good support and treatment	People with learning disabilities should get the help they need from health services, applying any reasonable adjustments required. Health professionals should listen more to the families and support staff of people with learning disabilities because they usually know most about the people they support and what help they need. Health staff should not assume that relatives or paid support staff of a person with learning disabilities will provide care while the person is in hospital; any such support must be discussed and agreed, taking account of their needs and supporting them appropriately. Case studies highlighted good positive practice in planning and delivering care
Patient Experience Network (not for profit organisation)<br><br>CHANGE (national human rights organisation led by disabled people)[58]	https://patientexperiencenetwork.org/wp-content/uploads/2019/10/Hidden-Voices-of-Maternity-Executive-Summary-FINAL-260815-2.pdf	2015	Hidden Voices of Maternity: Parents with Learning Disabilities Speak Out	To capture the experience of parents with learning disabilities and offer recommendations for service improvements to support care to become more person- and family-centred	Provide training for health professionals to better support parents with learning disability, improve accessibility to services. Establish a visible lead in a provider organisation whose role is to support learning disabilities as opposed to mental health or other area

Author	Webpage	Year	Title	Aims	Key information
Programme led by the University of Bristol and commissioned by the Healthcare Quality Improvement Partnership on behalf of NHS England[45]	https://www.bristol.ac.uk/media-library/sites/sps/leder/LeDeR annual report October 2016_FINAL v8.pdf	2016	The Learning Disabilities Mortality Review Annual Report	To evaluate the LeDeR programme	The 2015-2016 report found that there was a significantly higher mortality rate for people with learning disabilities compared to people without. Common underlying causes of mortality were circulatory and respiratory diseases and cancers. A high proportion of deaths were from causes classified as amenable to good medical care
The Westminster Commission on Autism[107]	https://westminsterautismcommission.files.wordpress.com/2016/03/ar1011_ncg-autism-report-july-2016.pdf	2016	A Spectrum of Obstacles<br><br>An Inquiry into Access to Healthcare for Autistic People	To highlight what good quality, person centred healthcare, tailored to the needs of those on the autistic spectrum, can achieve. The report is a call for ensuring equal access to quality healthcare for all on the autistic spectrum and to make this widespread and institutionalised	The report follows a seven-month inquiry chaired by Barry Sheerman MP. Consultation with over 900 autistic people, families and professionals to investigate issues highlighted in the inquiry. The report revealed obstacles that autistic people encounter when accessing healthcare, and presents six recommendations around: Training; Inspection; Data; Annual Health Checks; Leadership; and Resources.

Author	Webpage	Year	Title	Aims	Key information
National Quality Board[103]	https://www.england.nhs.uk/wp-content/uploads/2017/03/nqb-national-guidance-learning-from-deaths.pdf	2017	National Guidance on Learning from Deaths	To provide a framework for NHS Trusts and NHS Foundation Trusts on identifying, reporting, investigating and learning from deaths in care	Guidance to providers:<br>- All deaths of people with learning disabilities aged four years and older are subject to review using LeDeR methodology<br>- The LeDeR programme is currently being rolled out across England. Full coverage is anticipated in all Regions by the end of 2017. If there is a death of a person with learning disabilities in an acute setting in an area that is not yet covered by the LeDeR programme, Trusts are recommended to use the SJR process or a methodology of equivalent quality that meets the requirements for the data that must be collected as an interim measure
Programme led by the University of Bristol and commissioned by the Healthcare Quality Improvement Partnership on behalf of NHS England[46]	https://www.hqip.org.uk/wp-content/uploads/2018/05/LeDeR-annual-report-2016-2017-Final-6.pdf	2018	The Learning Disabilities Mortality Review Annual Report	To evaluate the LeDeR programme	The 2017 annual report suggested that 1,311 deaths were notified to the programme. Most people male (57%), single (96%) and of white ethnic background (93%). Just over a quarter had mild learning disabilities (27%), 33% had moderate learning disabilities, 29% severe learning disabilities and 11% profound or multiple learning disabilities. Approximately one in ten usually lived alone and had been in an out-of-area placement (9%)
NHS England[104]	https://www.england.nhs.uk/wp-content/uploads/2018/10/ask-listen-do-for-organisations-and-practitioners-v1.pdf	2018	Ask Listen Do Resource for Organisations and Practitioners	Tips for health, social care and education organisations and practitioners. Making feedback, concerns and complaints easier for people with a learning disability, autistic people, families and carers	This Ask, Listen, Do resource is for social care, health and education organisations, large or small, and their practitioners who support people with a learning disability, and autistic people. Four key themes within the resource; partnership, communication, processes and leadership

Author	Webpage	Year	Title	Aims	Key information
Mencap and The National Autistic Society: Treat me well campaign[105]	https://www.mencap.org.uk/get-involved/campaign-mencap/treat-me-well/announcement-learning-disability-training-health-and	2018	Oliver McGowan mandatory training in learning disability and autism	Train health and social care staff to provide better health and social care outcomes for people with a learning disability and autism, focused on raising awareness and understanding. The training is co-designed and co-delivered by people with a learning disability, autism, family carers and experts in the subject matter	A wider roll-out of the training is underway, with plans to evaluate. Mencap has already delivered the training to around 1,800 healthcare staff. 98% of participants said they wanted to change the way they deliver healthcare for people with a learning disability after taking part
Paula McGowan[48]	https://www.england.nhs.uk/blog/ask-listen-do-olivers-story/	2018	Blog<br><br>Ask Listen Do: Oliver's Story	Demonstrating the importance of the principles of Ask, Listen Do for healthcare providers of those with a learning disability to better understand and manage situations safely	Oliver had mild cerebral palsy, focal epilepsy and mild autism, and was admitted to hospital due to seizure activity. Oliver's health deteriorated and he passed away a few weeks later due to neuroleptic malignant syndrome, a rare but serious side effect of antipsychotic medications given to control his agitation in hospital, caused by his epilepsy and autism

Author	Webpage	Year	Title	Aims	Key information
NHS Improvement[106]	https://www.england.nhs.uk/wp-content/uploads/2020/08/v1.17_Improvement_Standards_added_note.pdf	2018	The learning disability improvement standards for NHS Trusts	Develop new standards to help NHS Trusts measure the quality of care they provide to people with learning disabilities, autism or both	The report comprises four key standards; respecting and protecting rights, inclusion and engagement, workforce and specialist learning disability services
Programme led by the University of Bristol and commissioned by the Healthcare Quality Improvement Partnership on behalf of NHS England[47]	https://www.hqip.org.uk/resource/the-learning-disabilities-mortality-review-annual-report-2018/ - .XkP6Wi7FIy4	2019	The Learning Disabilities Mortality Review Annual Report	To evaluate LeDeR programme	The 2018 annual report suggested that 4,302 deaths were notified to the programme, approximately 86% of the estimated number of deaths of people with learning disabilities in England each year. The proportion of people with learning disabilities dying in hospital is higher (62%) than in the general population (46%). Almost a half (48%) of deaths received care that the reviewer felt met or exceeded good practice. The proportion of deaths from people from Black, Asian and Minority Ethnic groups was lower (10%), than that from the population in England as a whole (14%). However, children and young people from BAME groups were overrepresented in deaths of people with learning disabilities
NHS England[83]	https://www.longtermplan.nhs.uk/publication/nhs-long-term-plan/	2019	The NHS long term plan	To set out a long term plan to make the NHS fit for the future	Providing the right care and support for children with a learning disability is part of long term plan. The plan specifies that the whole NHS will improve its understanding of the needs of people with learning disabilities and autism, and work together to improve their health and wellbeing. NHS staff will receive information and training on supporting people with a learning disability and/ or autism. National learning disability improvement standards will be implemented and will apply to all services funded by the NHS

Author	Webpage	Year	Title	Aims	Key information
NHS England and NHS improvement[49]	https://improvement.nhs.uk/documents/5472/190708_Patient_Safety_Strategy_for_website_v4.pdf	2019	The NHS patient safety strategy	To develop a patient safety culture and a patient safety system	Must ensure people with a learning disability are more visible; that they are listened to; and that reasonable adjustments are made to ensure they have better access to healthcare. Mandatory training on learning disability and autism to give health and care staff the knowledge and skills to accomplish this. Need understanding of safety issues: reduce harm from the effects of inappropriate psychotropic medicine use, care and treatment reviews. By 2023/24 all NHS-commissioned care will meet the learning disability improvement standards
Department of Health and Social Care[84]	https://www.gov.uk/government/consultations/learning-disability-and-autism-training-for-health-and-care-staff	2019	Consultation outcome. Learning disability and autism training for health and care staff	To gain a better understanding of how to ensure that patients and service users receive safe, effective and dignified care and that those who provide care have the knowledge, skills and behaviours to support people with learning disabilities and autistic people	Mandatory learning disability and autism training was one of the commitments made in in the Government's response to the second annual report of the LeDeR Programme. Training should focus on understanding learning disability and autism, the legislative context and making reasonable adjustments
Healthcare Safety Investigation Branch[50]	https://www.hsib.org.uk/documents/139/hsib_interim_bulletin_undiagnosed_cardiomyopathy_autism.pdf	2019	Undiagnosed cardoimyopathy of a young person with Autism	To investigate the death of an autistic patient with an undiagnosed heart problem	Identified safety issues included: The patient was not seen by a learning disability specialist and their altered physiological markers were attributed to emotional distress. Lack of national guidelines on aesthetic assessment and threshold for patients with learning disabilities and autism

Author	Webpage	Year	Title	Aims	Key information
Care Quality Commission[51]	https://www.cqc.org.uk/help-advice/your-stories/declare-your-care-people-learning-disabilities	2020	Declare Your Care: People with learning disabilities	A year-long, campaign focusing on four key population groups which have lower awareness of CQC	Survey findings revealed that people with a learning disability are more likely to regret not complaining about poor care than those without. The main reasons they or their carers want to raise a concern were: lack of information about a health condition and treatment options are not well explained. Stories presented
NIHR dissemination centre[52]	https://content.nihr.ac.uk/nihrdc/themedreview-04326-BCAHFA/Better-Health_Care-For-FINALWEB.pdf	2020	NIHR themed review: health and care services for people with learning disabilities	To review NIHR research on health and care services for people with learning disabilities	There is consistent evidence from confidential enquiries into unexpected deaths that general hospitals vary greatly in how well they make adjustments for patients who have learning disabilities. Research indicates problems in how hospitals implement these recommendations including confidence of general hospital staff in addressing the needs of patients. Learning disability nurses were found to make valued contributions to care. Little is known about what difference patient passports make to how well staff are able to adjust the care they provide

Author	Webpage	Year	Title	Aims	Key information
Programme led by the University of Bristol and commissioned by the Healthcare Quality Improvement Partnership on behalf of NHS England[11]	https://www.hqip.org.uk/wp-content/uploads/2020/07/LeDeR_2019_annual_report_FINAL.pdf	2020[a]	The Learning Disabilities Mortality Review Annual Report	To evaluate the LeDeR programme	The 2019 annual report suggested that 7,145 deaths were notified to the programme, 6,629 were adults and 516 were children (4-17y). The review process had been completed for 45% of these deaths. 58% were males; 90% were white British; 30% had mild learning disabilities, 33% had moderate learning disabilities, 27% severe learning disabilities and 10% profound and multiple learning disabilities. People with profound and multiple learning disabilities, and people from Black, Asian and Minority Ethnic groups died disproportionately at younger ages

Notes. CQC = Care Quality Commission, DDA = Disability Discrimination Act, GP = General Practitioner, LeDeR = Learning Disabilities Mortality Review, MCA = Mental Capacity Act, NHS = National Health Service, NICE = National Institute for Health and Care Excellence, NIHR = National Institute for Health Research, SJR = Structured Judgement Review.

[a]This report was published in July 2020 after our initial grey literature search.
