# Supplementary material for: Exploring patient safety outcomes for people with learning disabilities in acute hospital settings: a scoping review
Source: BMJ Open. 2021 May 19;11(5):e047102. doi: 10.1136/bmjopen-2020-047102 (PMC8137174; doi:10.1136/bmjopen-2020-047102)
Supplement: Author's manuscript [file bmjopen-2020-047102.draft_revisions.pdf]

BMJ Open is committed to open peer review. As part of this commitment we make the peer review history of every article we publish publicly available.

When an article is published we post the peer reviewers' comments and the authors' responses online. We also post the versions of the paper that were used during peer review. These are the versions that the peer review comments apply to.

The versions of the paper that follow are the versions that were submitted during the peer review process. They are not the versions of record or the final published versions. They should not be cited or distributed as the published version of this manuscript.

BMJ Open is an open access journal and the full, final, typeset and author-corrected version of record of the manuscript is available on our site with no access controls, subscription charges or pay-per-view fees (http://bmjopen.bmj.com).

If you have any questions on BMJ Open's open peer review process please email info.bmjopen@bmj.com

BMJ Open

Exploring patient safety outcomes for people with learning disabilities in acute hospital settings: A scoping review

Journal:	*BMJ Open*
Manuscript ID	bmjopen-2020-047102
Article Type:	Original research
Date Submitted by the Author:	19-Nov-2020

Complete List of Authors:	Louch, Gemma; Bradford Teaching Hospitals NHS Foundation Trust, Bradford Institute for Health Research; NIHR Yorkshire and Humber Patient Safety Translational Research Centre<br>Albutt, Abigail; Bradford Teaching Hospitals NHS Foundation Trust, Bradford Institute for Health Research; NIHR Yorkshire and Humber Patient Safety Translational Research Centre<br>Joanna Harlow-Trigg , Joanna; University of Leeds, School of Psychology<br>Moore, Sally; Bradford Teaching Hospitals NHS Foundation Trust, Bradford Institute for Health Research<br>Smyth, Kate ; NIHR Yorkshire and Humber Patient Safety Translational Research Centre; Lancashire Teaching Hospitals NHS Foundation Trust<br>Ramsey, Lauren; Bradford Teaching Hospitals NHS Foundation Trust, Bradford Institute for Health Research ; NIHR Yorkshire and Humber Patient Safety Translational Research Centre<br>O'Hara, Jane; University of Leeds, School of Healthcare; NIHR Yorkshire and Humber Patient Safety Translational Research Centre

Keywords:	Quality in health care < HEALTH SERVICES ADMINISTRATION & MANAGEMENT, Organisation of health services < HEALTH SERVICES ADMINISTRATION & MANAGEMENT, HEALTH SERVICES ADMINISTRATION & MANAGEMENT

SCHOLARONE™
Manuscripts

I, the Submitting Author has the right to grant and does grant on behalf of all authors of the Work (as defined in the below author licence), an exclusive licence and/or a non-exclusive licence for contributions from authors who are: i) UK Crown employees; ii) where BMJ has agreed a CC-BY licence shall apply, and/or iii) in accordance with the terms applicable for US Federal Government officers or employees acting as part of their official duties; on a worldwide, perpetual, irrevocable, royalty-free basis to BMJ Publishing Group Ltd ("BMJ") its licensees and where the relevant Journal is co-owned by BMJ to the co-owners of the Journal, to publish the Work in this journal and any other BMJ products and to exploit all rights, as set out in our licence.

The Submitting Author accepts and understands that any supply made under these terms is made by BMJ to the Submitting Author unless you are acting as an employee on behalf of your employer or a postgraduate student of an affiliated institution which is paying any applicable article publishing charge ("APC") for Open Access articles. Where the Submitting Author wishes to make the Work available on an Open Access basis (and intends to pay the relevant APC), the terms of reuse of such Open Access shall be governed by a Creative Commons licence – details of these licences and which Creative Commons licence will apply to this Work are set out in our licence referred to above.

Other than as permitted in any relevant BMJ Author's Self Archiving Policies, I confirm this Work has not been accepted for publication elsewhere, is not being considered for publication elsewhere and does not duplicate material already published. I confirm all authors consent to publication of this Work and authorise the granting of this licence.

For peer review only

Exploring patient safety outcomes for people with learning disabilities in acute hospital settings: A scoping review

Gemma Louch[1 2], Abigail Albutt[1 2], Joanna Harlow-Trigg[1 4], Sally Moore[1], Kate Smyth[2 3], Lauren Ramsey[1 2] and Jane K. O'Hara[1 2 5]

1 Yorkshire Quality and Safety Research Group, Bradford Institute for Health Research, Bradford, UK

2 NIHR Yorkshire and Humber Patient Safety Translational Research Centre, UK

3 Lancashire Teaching Hospitals NHS Foundation Trust

4 School of Psychology, University of Leeds, Leeds, UK

5 School of Healthcare, University of Leeds, Leeds, UK

Corresponding author:
Dr Gemma Louch
Gemma.Louch@bthft.nhs.uk

Twitter @Gemma_Louch

Contributors:
GL conceived the idea for the scoping review, led all stages of the scoping review and drafted the manuscript. AA contributed to all stages of the scoping review. KS reviewed the protocol, contributed to the search strategy and worked with the research team to develop materials for the wider Patient and Public Involvement and Engagement (PPIE) approach. JHT, SM and LR contributed to the literature searching, screening and data extraction. JOH contributed to the consistency checks and wrote the first draft of the discussion. All authors provided comments and approved the final version.

Funding:
The research was funded by the National Institute for Health Research Yorkshire and Humber Patient Safety Translational Research Centre (NIHR YH PSTRC). The views expressed in this article are those of the author(s) and not necessarily those of the NHS, the NIHR or the Department of Health and Social Care.

Competing interests: None declared.

Patient consent for publication: Not required.

For peer review only

For peer review only - http://bmjopen.bmj.com/site/about/guidelines.xhtml

Abstract

Objectives

To produce a narrative synthesis of published academic and grey literature focussing on patient safety outcomes for people with learning disabilities in an acute hospital setting.

Design

Scoping review with a narrative synthesis.

Methods

The review followed the six stages of the Arksey and O'Malley framework. We searched four research databases from January 2000 to November 2019, in addition to hand searching and backwards searching using terms relating to our eligibility criteria – patient safety and adverse events, learning disability, and hospital setting. Following stakeholder input, we searched grey literature databases and specific websites of known organisations until March 2020. Potentially relevant articles and grey literature materials were screened against the eligibility criteria. Findings were extracted and collated in data charting forms.

Results

41 academic articles and 34 grey literature materials were included, and we organised the findings around six concepts: 1) Adverse events, patient safety and quality of care; 2) Role of family and carers; 3) Understanding needs in hospital; 4) Maternal and infant outcomes; 5) Post-operative outcomes; and 6) Supporting initiatives, recommendations and good practice examples.

Conclusion

People with learning disabilities appear to experience poorer patient safety outcomes in hospital. The involvement of family and carers, and understanding and effectively meeting the needs of people with learning disabilities may play a protective role. Promising interventions and examples of good practice exist, however many of these have not been implemented consistently and warrant further robust evaluation.

Keywords

Learning disability, patient safety, hospital, adverse events, intellectual disability

Strengths and limitations of this study

- A key strength is the synthesis of both academic and grey literature materials
- A further strength is our approach to patient and public involvement and engagement through the review process
- We did not conduct formal quality assessments and are therefore unable to make reflections and comparisons of article quality

For peer review only - http://bmjopen.bmj.com/site/about/guidelines.xhtml

Introduction

Inequalities in health and inequities in access to healthcare and technologies are a persistent and significant problem.[1 2 3] It is clear from previous research that certain demographic factors are associated with increased likelihood of poorer health, and variation in the use of and access to healthcare services.[4 5]

One population that may experience greater vulnerabilities in terms of health and healthcare inequalities are people with learning disabilities. These vulnerabilities might arise as a result of barriers to accessing services and challenges associated with service organisation and delivery.[6] Learning disabilities are defined as 'the presence of a significantly reduced ability to understand new or complex information, to learn new skills (impaired intelligence), with a reduced ability to cope independently (impaired social functioning) which started before adulthood, with a lasting effect on development'[a]. In this review we have also drawn from the definition presented in the White Paper *Valuing People*,[7] which states that learning disability includes the presence of:

- a significantly reduced ability to understand new or complex information, to learn new skills (impaired intelligence), with;
- a reduced ability to cope independently (impaired social functioning);
- which started before adulthood, with a lasting effect on development.

This broad definition includes adults with autism who also have learning disabilities, but not those with a higher-level autistic spectrum disorder, such as some people with Asperger's Syndrome. Learning disability is the term most commonly used in the UK, although it is recognised as being synonymous with intellectual disability.[8]

In 2013, the final report of a Confidential Inquiry into Premature Deaths of People with Learning Disabilities (CIPOLD) in England was published.[9] The report found that people with learning disabilities have higher rates of avoidable death compared to the general population, and that avoidable deaths arising from causes relating to poorer quality healthcare were more common in this population. On average, the life expectancy of people with learning disabilities is shorter than the general population.[10] The 2019 Learning Disabilities Mortality Review (LeDeR) report highlighted that people with learning disabilities died from an avoidable medical cause of death twice as frequently as people in the general population, and that the greatest difference between people with learning disabilities and the general population was in relation to medical causes of death which are treatable with access to timely and effective healthcare.[11]

In the UK the need for accessible healthcare environments for people with autism is recognised,[12] and in 2019, the Government announced plans to pilot and then roll out learning disability and autism mandatory training for health and care staff in England[b].

[a]https://www.datadictionary.nhs.uk/data_dictionary/nhs_business_definitions/l/learning_disability_de.asp?shownav=1
[b]https://www.gov.uk/government/consultations/learning-disability-and-autism-training-for-health-and-care-staff

For peer review only - http://bmjopen.bmj.com/site/about/guidelines.xhtml

Furthermore, national projects such as Stopping Over-Medication of People with a Learning Disability, Autism or Both (STOMP)[c] have addressed issues around medicines practices. Although there is increasing interest in this important issue from academics, healthcare staff, managers and policy makers, much of this has focussed on health inequalities and healthcare access more generally. What has been lacking to date is a critical examination of this issue as a patient safety phenomenon. This is important, as it opens up new avenues for conceptualising this problem, along with different framings for potential improvement and service development.

There is clear evidence that people with learning disabilities may be more at risk in terms of patient safety in hospital as well as known challenges around recognising and reporting patient safety incidents in this population.[13 14 15] Therefore, the need to bring together what is known about the safety of people with learning disabilities receiving healthcare, is clear.

In this review, we aimed to produce a narrative synthesis of published academic and grey literature focussing on people with learning disabilities in an acute hospital setting. We limited this review to the hospital setting because we were particularly interested in the care people with learning disabilities receive in a setting that may be predominantly related to physical health. We aimed to generate evidence that may facilitate the development of more tailored patient safety interventions for people with learning disabilities in an acute hospital setting. Our specific objectives were to:

1) Understand patient safety and adverse events in this population;
2) Explore protective factors and potential explanatory mechanisms;
3) Identify patient safety interventions, improvement initiatives, recommendations and examples of good practice.

Methods

A scoping review was considered the most suitable approach to produce a comprehensive, yet broad overview of the topic area[16 17] We used Arksey and O'Malley's[18] six stage framework and subsequent amendments to guide the review.[16 19] The stages include: (1) identifying the research question(s); (2) identifying relevant research studies; (3) selecting relevant research studies; (4) charting the data; (5) collating, summarising and reporting the study findings; and (6) consulting with key stakeholders throughout the process. The review has been drafted in line with the PRISMA Extension for Scoping Reviews (PRISMA-ScR).[20] We developed a broad search strategy, informed by the PRISMA Extension for Systematic Reviews with a Focus on Health Equity (PRISMA-Equity, 2012).[21]

Patient and public involvement and engagement (PPIE)

Our review team includes a lay representative (co-author) who provided input into the protocol, reviewed the search strategy and helped develop materials for the wider PPIE approach. We invited stakeholders to contribute search terms and assist in identifying grey literature. Stakeholders included representatives from the Yorkshire Quality and Safety Research (YQSR) Group patient panel, representatives from the NIHR Yorkshire and Humber Patient Safety Translational Research Centre (NIHR YH PSTRC) Citizen Participation Group and healthcare staff.

[c] https://www.england.nhs.uk/learning-disabilities/improving-health/stomp/

For peer review only - http://bmjopen.bmj.com/site/about/guidelines.xhtml

Eligibility criteria

The ‘Population-Concept-Context’ (PCC) approach was used to specify study characteristics.[16] Inclusion and exclusion criteria were developed and iteratively refined as the review progressed. Studies reporting on patient safety, adverse events, protective factors, potential explanatory mechanisms, intervention and improvement initiatives, recommendations and good practice examples related to these topic areas were eligible. There was no restriction of study design, quantitative and qualitative methodologies were eligible for inclusion, and we limited the search to English language only.

Inclusion criteria

- Articles that report on people with learning disabilities as the core focus (population). Articles may use terms synonymous with learning disability such as intellectual disability or refer to a condition related to learning disability, for example autism[d], attention deficit hyperactivity disorder[e] or Down Syndrome.
- Articles that investigate adverse events, patient safety, protective factors, potential explanatory mechanisms, patient safety interventions and improvement initiatives, recommendations and good practice examples (concept).
- Articles relating to patients receiving care in an acute hospital setting (context). No restriction on age.
- Articles relating to any country (context).
- Study type: No restriction - qualitative, quantitative, mixed methods, case studies, primary research, retrospective review, systematic or scoping reviews/ integrative reviews / meta-synthesis.
- Language: only articles published in the English Language due to lack of resources for an interpreter.

Exclusion criteria

- Articles relating to primary care settings and inpatient mental health settings.
- Articles focussing on patient experience/ satisfaction.
- Articles focussing on a specific drug treatment or procedure without a non-learning disability comparison group.

Information sources and search strategy

Academic literature search

The search terms built on terms used in prior reviews framed around the eligibility criteria.[15 25 26 27 28] An initial limited search of OVID MEDLINE was conducted (Appendix 1). The search strategy was peer reviewed by a Knowledge and Information Librarian reviewer using the Peer Review of Electronic Search Strategies (PRESS),[29] and reviewed by academic researchers (patient safety), lay representatives and learning disability healthcare professionals. Following the initial search, all four included databases were searched: MEDLINE, CINHAL, PsycINFO and Web of Science from 2000[f] until November 28th 2019. The search was organised in three blocks: Block 1 – terms relating to learning disability (combined with OR); Block 2 – terms relating to adverse events and patient safety

[d]Autism and learning disabilities are often co-associated.[22 23]
[e]High comorbidity for learning disabilities and attention deficit hyperactivity disorder.[24]
[f]The time period searched from was 2000 in line with the seminal publication of ‘To Err is Human: Building a Safer Health System’ as this publication arguably launched the modern patient safety movement.[30]

For peer review only - http://bmjopen.bmj.com/site/about/guidelines.xhtml

(combined with OR); Block 3 – terms relating to acute hospital setting (combined with OR). Blocks 1-3 were combined with the AND function. The reference lists of included articles were assessed, and we hand searched targeted journals including: the British Journal of Learning Disabilities, Journal of Learning Disabilities, Learning Disabilities, Journal of Intellectual Disability Research, BMJ Quality and Safety, Journal of Patient Safety, Health Expectations, BMC Health Services Research, BMJ Open.

Grey literature search

The grey literature search included suggestions made via stakeholder input, such as terms to search, known publicly available materials and specific organisations to search online (Appendix 2). We searched using the same combinations of terms relating to our eligibility criteria (e.g. 'patient safety and learning disability', learning disability and hospital', 'learning disability and adverse events'). All the online materials returned were initially screened according to title/summary information. In addition, the first 100 pages of Google, Google Scholar and all materials returned from OpenGrey and Royal College of Nursing Database were screened. The latest date for grey literature searches was 10th March 2020.

Study selection

Identified articles were collated in reference software (EndNote) and duplicates removed. Study selection involved two levels of screening: 1) title and abstract 2) full-text. Three reviewers (GL, AA, JHT) screened at title and abstract level according to the eligibility criteria, and 10% were independently checked to assess agreement. Articles that appeared to be eligible were screened at full-text level. When a full-text was unavailable, authors were contacted directly. We were unable to obtain two full-texts. Two independent reviewers assessed the full-text articles (GL, AA) and at this stage the reasons for exclusion were recorded. There were no discrepancies between reviewers regarding the eligibility of articles. Two authors carried out the grey literature search (GL, AA), and one author independently screened the potential grey literature for inclusion (SM), and 10% were independently checked to assess agreement.

Charting the data

Standardised data collection forms were developed and information from academic articles and grey literature material were collated into separate data collection forms, which were piloted prior to full data extraction.[19] For academic articles, key data were extracted including: publication year, publication type, country, study design, population, and summary information relating to adverse events, patient safety focus, protective factors, potential explanatory mechanisms, patient safety intervention or improvement initiatives, recommendations and good practice examples. Following piloting, two reviewers (AA, JHT) independently extracted the data from all included articles, and one reviewer checked 10% of the data extracted for consistency (JOH).

Study quality was not assessed as the aim of the review was to synthesise the emerging evidence within the area rather than assess quality of individual articles. The grey literature data collection form was amended from the research article data collection form. Three reviewers (SM, AA, LR) independently extracted the data from all included publications using the adapted data collection form, and one reviewer checked 10% of the data extracted for consistency (JOH).

For peer review only - http://bmjopen.bmj.com/site/about/guidelines.xhtml

Data synthesis

Data were collated in two spreadsheets, one for academic articles and one for grey literature. A narrative synthesis followed to develop a narrative description of the findings and to highlight concepts that key findings could be organised around.[31 32] Authors (GL, AA, SM, LR, JOH) held meetings to discuss the key findings and generate concepts.

Results

Title and abstract screening identified 133 articles eligible for full-text screening, and 36 articles were eligible for inclusion in the review. A further 10 articles were included via backward and hand searching. Where studies appeared in review articles that met the eligibility criteria, these were not analysed separately and excluded (n = 5). In total, 41 articles were included (see figure 1). The grey literature search identified 92 potentially eligible materials, and 34 were included.

Summary characteristics

Characteristics of included articles and grey literature materials are displayed in online supplementary tables 1 and 2.

Of the academic articles, fourteen related to paediatric patients, seven to adult patients, five to pregnant women/infant outcomes, two to healthcare staff, one to healthcare staff and carers, one to adults, paid carers and hospital nurses, one to parents or guardians, three were improvement projects, and seven articles related to hospital patients/setting more generally or did not specify the participants in more detail. All studies and reviews were conducted in high-income countries. Fourteen articles were from the USA, thirteen were from the UK, nine were from Australia, two were from Canada and one each was from Taiwan, Hungary and The Netherlands. Seventeen articled were cohort studies, nine were a type of literature review, four articles used mixed methods, three were commentaries, three qualitative, three improvement projects, one case note audit and one case study. Eleven articles referred specifically to intellectual disability, eight to learning disability, eight to Down Syndrome, three to intellectual and developmental disability, three to autism, three to cognitive impairment, two to complex communication need, two to communication disability, and one to attention deficit hyperactivity disorder.[g]

Key concepts

Our data synthesis generated six concepts: 1) Adverse events, patient safety and quality of care; 2) Role of family and carers; 3) Understanding needs in hospital; 4) Maternal and infant outcomes; 5) Post-operative outcomes; and 6) Supporting initiatives, recommendations and good practice.

Adverse events, patient safety and quality of care

Eight articles concentrated on either specific types of adverse events, quality of care, and patient safety,[14 15 33 34 35 36 37 38] three of these were literature reviews. A review of falls in adult hospital patients with communication disability found that most studies reported communication disability to be related to falls, either increasing or decreasing risk.[35] A review of the experience of iatrogenic harm during hospitalisation for children with intellectual

[g]Throughout the results section we use the same terms as those used in the original articles and grey literature materials.

For peer review only - http://bmjopen.bmj.com/site/about/guidelines.xhtml

disability found that there are specific aspects of hospitalisation that expose children with intellectual disability to harms that are preventable, avoidable and not experienced to the same extent by children without intellectual disability.[15] This is consistent with the findings of a study reporting that the rate of infectious, respiratory, and gastrointestinal adverse events are higher in children with Down Syndrome[37] and a study that found that children with cognitive impairment received lower doses of analgesia and sedation medication, although the authors acknowledged it was not clear whether this was due to lower requirements or inadequate assessment.[33] Interestingly, a study assessing readmission found no significant difference in 30-day readmission rates for people with and without learning disabilities, but that 69% of readmissions of people with learning disabilities were potentially preventable.[36]

The third review article investigated the care or safety of adults with communication disabilities in hospital and concluded that patient safety incident and adverse event reporting lacked detail, and that successful advocacy affected outcomes, suggesting that when advocacy was ignored outcomes were worse. The review reported adverse event themes, including isolation due to limited methods to communicate with nurses, and that carers had a protective role in uncovering or preventing adverse events.[34]

In a mixed-methods study staff survey respondents reported feeling less confident about managing challenging behaviour and always delivering safe care to children and young people with learning disabilities, compared to children and young people without learning disabilities, as well as reporting that the environment was less safe for meeting the needs of children and young people with learning disabilities compared to those without.[38] A further mixed-methods study described the challenges in preventing and monitoring patient safety issues for people with intellectual disabilities in NHS acute hospitals, and concluded that hospitals seemed to lack effective systems for identifying patients which made monitoring safety incidents difficult.[14] Furthermore, the study reported that staff do not always readily identify patient safety issues or report them, with incident reports commonly focussed on events causing immediate or potential physical harm, and that safety issues were mostly related to delays and omissions of care.[14]

A wealth of grey literature further evidences vulnerabilities in terms of adverse events, quality of care, and patient safety for people with learning disabilities.[9 11 39 40 41 42 43 44 45 46 47 48 49 50 51 52] This included influential reports such as the 2013 Confidential Inquiry into Premature Deaths of People with Learning Disabilities (CIPOLD)[9] and the subsequent Learning Disabilities Mortality Review (LeDer) programme annual reports, which evaluate the LeDer programme.[11 45 46 47]

Role of family and carers

Six articles highlighted the significant role of families and paid carers.[6 25 53 54 55 56] Two articles were literature reviews. One review presented a synthesis of previous work around parental experience of hospitalisation with a child with intellectual disability, care quality and safety. A working model for professional parent partnership was developed which reinforced the importance of hospital/multidisciplinary approaches to care centring on the child, understanding previous negative experiences and negotiating care, and shared learning to lessen reliance on parental presence.[54] The second review article evaluated how hospital systems respond to adults with intellectual disability, their families and carers. Key themes included; individual fear of hospital encounters, reliance on paid family carers for basic

For peer review only - http://bmjopen.bmj.com/site/about/guidelines.xhtml

needs and advocacy, responsibilities and staff knowledge, skills and attitudes.[25] A qualitative interview study, which explored paid carers' roles in supporting adults with developmental disability and complex communication needs described how paid carers are often motivated by perceived responsibility for safety, well-being and communication, but that their role can sometimes be blurred with nursing and family carer roles.[53]

A mixed methods study aiming to identify factors that affect carer involvement for people with intellectual disabilities in acute hospitals presented a model for clarifying carer involvement that sought to highlight the degree to which carers are 'workers' contributing to basic nursing care, and the degree to which carers are experts or non-experts. [56] The authors suggested that making these two aspects explicit might facilitate staff to understand carer contributions more comprehensively. A key finding from a qualitative study with medical practitioners concluded that practitioners make limited use of "reasonable adjustments" and turned to caregivers to facilitate communication and manage behaviours likely to upset hospital routines.[55] Finally, a quantitative case note audit highlighted steps to deliver high-quality care to people with learning disabilities; key to achieving this was ensuring that family or carers are involved in discharge planning.[6]

In terms of grey literature, a doctoral thesis which investigated emergency healthcare from the perspective of the carers of people with learning disabilities, highlighted the relationship staff had with both service users and carers as fundamental to a high quality service.[57]

Understanding needs in hospital

Six articles had content relating to the needs of people with learning disabilities in hospital[58 59 60 61 62 63] three of these were reviews.[58 59 60] One review concluded that to ensure nurses do as much as possible to identify risk they must recognise prejudices and overcome them, develop further understanding of learning disabilities and acknowledge the rights of people with learning disabilities, and collaborate with carers and professionals.[59] Similarly, a mixed methods literature review around communication with those with complex communication needs recognised the importance of collaborating effectively with carers, as well as access to personally held written health information, inter-agency communication, devoting time to communication, and access to communication tools and aids.[60] The third review article, assessing evidence around the promotion of health, safety and welfare of adults with learning disabilities in acute care emphasised the importance of care provision, communication, staff attitudes, staff knowledge, supporters and carers and the physical environment.[58] Crucially, communication was highlighted as a fundamental issue in this article, such that people with learning disabilities often have difficultly communicating their needs. The review presented strategies and resources that may support this such as videos, accessible booklets, augmentative and alternative communication and pictures/symbols.

To help improve the inpatient experience of hospital patients with autism, a survey of parents and guardians with qualitative and quantitative items, highlighted the need for an individualised approach to assess and accommodate needs.[62] This approach was taken in a case study that described the plan of care for a patient with moderate level of learning disability scheduled for a tonsillectomy. The report gave a specific example of how investing time to understand a patient's need can improve experience.[63] When the patient's details were being checked, the door knocked into the patient's chair as staff entered the room for

For peer review only - http://bmjopen.bmj.com/site/about/guidelines.xhtml

equipment, and this exacerbated the patient's anxiety. This was acknowledged quickly and a do not disturb sign was placed on the door.

A commentary aiming to familiarise the paediatric nurse with autism and create a resource for successful inpatient treatment put forward key themes such as change is a challenge, consistent caregivers, safe environment, encouraging family involvement, ways of communicating, emotional triggers and reward systems and multidisciplinary team from admission.[61] Indeed, The NHS long term plan published in 2019,[64] emphasised that the whole NHS will improve its understanding of the needs of people with learning disabilities and autism, with plans in place for staff to receive training on supporting people with a learning disability and/ or autism alongside the implantation of national learning disability improvement standards. Furthermore, the government response to the consultation on learning disability and autism training for health and care staff also published in 2019, underlined the importance of gaining a better understanding of how to ensure that patients and service users receive safe, effective and dignified care, as the need to equip those providing care with the necessary skills, knowledge and behaviours.[65]

Maternal and infant outcomes

Five articles examined maternal and infant outcomes utilising a cohort design, either focussing on women with intellectual and developmental disabilities[66 67 68] intellectual disability and/or self reported learning difficulties[69] and attention deficit hyperactivity disorder[70] Higher rates of complications such as pre-eclampsia[66 69 68 70] preterm birth[68 69 70] low birth weight[69] and labour interventions including induction and caesarean[66 70] were reported. One study reported higher prevalence rates for hospital admission and emergency department visits during all critical postpartum periods for those with intellectual and developmental disabilities, and higher risk of repeated hospitalisations.[67]

A survey led by Patient Experience Network (not-for-profit organisation) and CHANGE (national human rights organisation) supported by NHS England, aimed to capture the experience of parents with learning disabilities.[71] Training for health professionals to better support parents with learning disability and improving accessibility to services were highlighted as essential.

Post-operative outcomes

Nine articles reported on post-operative outcomes utilising a cohort design.[72 73 74 75 76 77 78 79 80] The majority of articles focused on Down Syndrome[72 73 74 76 77 78] followed by cognitive impairment[79 80] and intellectual disability[75] Increased rates of complications[72 73 75 76 77 79 80] were reported in seven studies. However, in one study comorbidities rather than DS were a greater risk factor for complications[72] A longer length of stay was reported in four studies[73 75 76 77] with one further study reporting a similar length of stay for those with Down Syndrome compared to those without[78] In the same study, mortality and major complication rates were lower for patients with Down Syndrome[78] and a second study reported lower odds of in-hospital death for patients with Down Syndrome when controlling for other factors such as risk category and premature birth.[74] In four studies no differences in mortality were reported.[75 76 77 79]

For peer review only - http://bmjopen.bmj.com/site/about/guidelines.xhtml

Supporting initiatives, recommendations and good practice

Seven articles utilising diverse designs (including, case note audit, literature review, commentary, qualitative interview, and service improvement), reported either examples of initiatives to support safe care for people with learning disabilities in hospital, or recommendations to support good practice.[81 82 83 84 85 86 87] A qualitative content analysis of 60 documents mapped the content of existing hospital passports for people with intellectual disability and concluded that this approach can enhance safety and person-centred care, but acknowledged there is much variation between current hospital passports which may limit effectiveness[85] Three articles provided specific examples of how to enhance good practice, including a commentary highlighting how hospital pharmacists can contribute to safety when supporting people with intellectual disability in hospital,[81] a commentary focusing on the pre-surgical needs of those with Down Syndrome and how patient safety can be optimised[84] and a literature review presenting recommendations for the perioperative management of children with autism.[87]

Three articles described improvement work[82 83 86] One project identified areas of risk for people with intellectual disability whilst in hospital, and developed and successfully implemented a rapid risk assessment tool to assess immediate and potential risk, identify risk reduction actions and develop appropriate care bundles.[86] The second project identified core tasks of a specialist learning disability team to improve patient care for those with learning disabilities, examples included; educating acute staff, developing training materials for staff and trainees, considering consent issues and facilitating community support before discharge.[83] Finally, a mixed methods study comprising literature review and improvement work, developed care plans and an educational module. After completing the module, there was an increase in nurses' confidence when caring for people with learning disabilities.[82]

Further initiatives, recommendations and good practice examples were identified in the grey literature.[88 89 90 91 92 93 94 95 96 97 98 99 100 101] For brevity, we provide further information and signpost to these resources in online supplementary table 2.

For peer review only

Discussion

To the authors' knowledge, this is the first scoping review to synthesise both the academic and grey literature focusing on hospital patient safety outcomes for people with learning disabilities. Whilst, as a narrative synthesis we are unable to state unequivocally the relationship between having learning disabilities and safety outcomes, our findings do suggest that there are multiple ways in which people with learning disabilities might experience poorer outcomes compared with people without. Our review suggests that there are inequalities and inequities for a range of specific patient safety outcomes including adverse events, quality of care, maternal and infant outcomes and post-operative outcomes. This disparity needs urgent attention. Nonetheless, we did identify a range of potential protective factors, such as the roles of family and carers and the extent to which health professionals are able to understand the needs of people with learning disabilities. Research has focussed on developing interventions and good practice guidance, yet this is predominantly accounted for within the grey literature, meaning that robust evidence is still needed.

Some negative outcomes are likely through the 'direct effects' of having learning disabilities. For example, the increased incidence of co-morbidities in children with learning disabilities accounted for the increased likelihood of post-operative complications.[72] However, it is also clear that there are multiple 'indirect effects' of having learning disabilities that may amplify problems. Principal issues likely to manifest in differential outcomes included problems with communication (patients to staff, staff to patients, intra- and inter-agency), staff attitudes, variable effectiveness of 'flagging' of people with learning disabilities, the role of family and carers, and variation in the quality and level of healthcare received. These indirect effects fall squarely in the realm of quality and safety efforts, modifiable potentially through service redesign, increased resources, training, professional specialisation, and appropriate adaptation of practice.

We explore these issues through a patient safety 'lens', and what is perhaps most striking about our findings, is their lack of novelty. One of the earliest national reports within the UK – 'Healthcare for all'[41] – found similar issues, and made a series of recommendations. It is clear from our review that since this report, very little has changed in terms of the experience of people with learning disabilities and their families within acute care settings, either nationally or internationally. The exploration of this issue as a '*patient safety problem'* allows us to understand how, through the design of our healthcare system we create – and seek to solve – safety problems from the perspective of those moving through and navigating the system.

In an unrelated study, Fylan and colleagues examined the medicines management system for heart failure patients discharged from hospital into the community, and developed a framework called 'Gaps, Traps, Bridges and Props' which may be useful when thinking about our review findings.[102] '*Gaps'* occur in our systems at points of discontinuity or transition, and evidence from across patient safety literature that gaps in the structure and design of services create 'safety gaps' that present opportunities for problems for patients, especially when care is suboptimal or fails.[103 104] It is arguable that those patients with complex needs or specific vulnerabilities that require greater continuity of care, are more at risk when crossing these 'safety gaps' – in effect, their vulnerability amplifies the risk of experiencing a patient safety problem. In our review, it is evident that people with learning

For peer review only - http://bmjopen.bmj.com/site/about/guidelines.xhtml

disabilities may disproportionately suffer due to these gaps in healthcare systems. Examples of this would include poor inter agency communication,[60] and hospitals lacking effective systems for identifying patients.[14] Sometimes, the design of services/organisations goes beyond creating a '*gap'* – which may or may not result in a safety problem for patients. '*Traps'* are here defined as features of system design that *actively* make problems more likely. An example of a '*trap'* from our review is the need for training on learning disabilities for healthcare staff.[49 64 65] Without specific knowledge of, and training in caring for those with a range of learning disabilities, it is perhaps understandable that staff regularly fail to make reasonable adjustments to accommodate specific needs.[41 55]

This framing provides the possibility to ameliorate the issues that result, either through formalised '*bridges*', or further supporting the range of informal '*props'* that serve to reduce problems when care is suboptimal, or fails. '*Bridges'* are viewed as formalised features of a system, designed to span service gaps, and support continuity of care.[102] We found a number examples – from patient-held passports,[85 88] to specialist learning disability teams.[83] However, our review also found that these '*bridges'* are often inconsistently available or applied,[6] a position that could further amplify problems if staff have come to rely on them for support when needed. The most prevalent mechanism for supporting patients with learning disabilities came through the role of patients and carers. Although the need to reduce ambiguity about the role of the parent[54] and the importance of clarifying what carer involvement includes[56] were emphasised, we found a range of evidence that suggested families and carers regularly '*prop'* up services - from help with feeding and personal care,[25] to facilitating communication[55] and being involved in discharge planning[6] – and that without this '*prop'*, the outcomes for patients with learning disabilities may well be poorer.

Implications

Our review demonstrates the piecemeal and wide-ranging nature of the extant evidence, in terms of specific learning disabilities and outcomes of interest, and with a range of methodologies used. Therefore, we propose that research is needed to establish the burden of harm for people with learning disabilities as a result of patient safety incidents and poor quality of care, in hospital settings. This goes beyond learning from deaths – we need to understand what happens with care for people with learning disabilities more generally. Second, research needs to understand the mechanisms through which these effects might be seen. It is this approach that holds significant promise from the point of view of service improvement and redesign, as well as training and curriculum development. Put simply, we cannot change what we do not yet fully understand. Finally, attention must be given again to the existing recommendations from the range of reports already published.[9 11 39 41 45 46 47 93 101] There is already a wealth of learning about the problems that exist for people with learning disabilities and their families; what is needed now is policy level action.

Limitations

Despite an inclusive search strategy, relevant articles may not have been identified if they were not available in the sources searched. Additionally, due to the nature of the review, we did not conduct formal quality assessments and were therefore unable to make reflections and comparisons of article quality.

For peer review only - http://bmjopen.bmj.com/site/about/guidelines.xhtml

Conclusion

The academic and grey literature indicates that whilst in hospital, people with learning disabilities might experience poorer patient safety outcomes. The involvement of family and carers, and understanding the needs of people with learning disabilities in hospital were highlighted as potential protective factors. Many promising interventions and examples of good practice exist, however these may not be widely available or have been applied inconsistently.

For peer review only

Acknowledgements:
We are extremely grateful for the contributions from a number of people and groups. Thank you to the Yorkshire Quality and Safety Research (YQSR) Group patient panel and NIHR YH PSTRC Citizen Participation Group members for their contributions to the search strategy. Secondly, thank you to Amanda Mckie, Beth Fylan, Jonathan Benn, Kate Pickett, Steph Prady and Federica Bianchini for their contributions to the protocol draft and search strategy. Finally, thank you to Sara Ryan and John Baker for their feedback on the draft manuscript.

For peer review only

References

1 Arcaya MC, Arcaya AL, Subramanian SV. Inequalities in health: definitions, concepts, and theories. Global health action. 2015 Dec 1;8(1):27106.

2 Marmot M, Bell R. Fair society, healthy lives. Public health. 2012 Sep 1;126:S4-10.

3 Marmot M. Health equity in England: the Marmot review 10 years on. Bmj. 2020 Feb 25;368.

4 Cookson R, Propper C, Asaria M, Raine R. Socio-economic inequalities in health care in England. Fiscal Studies. 2016 Sep;37(3-4):371-403.

5 Mattox EA. Identifying vulnerable patients at heightened risk for medical error. Critical Care Nurse. 2010 Apr;30(2):61-70.

6 Sheehan R, Gandesha A, Hassiotis A, Gallagher P, Burnell M, Jones G, Kerr M, Hall I, Chaplin R, Crawford MJ. An audit of the quality of inpatient care for adults with learning disability in the UK. BMJ open. 2016 Apr 1;6(4).

7 Department of Health. Valuing people: a new strategy for learning disability for the 21st century. A White Paper. London: HMSO; 2001.

8 Cluley V. From “Learning disability to intellectual disability”—Perceptions of the increasing use of the term “intellectual disability” in learning disability policy, research and practice. British Journal of Learning Disabilities. 2018 Mar;46(1):24-32.

9 Heslop P, Blair P, Fleming P, Hoghton M, Marriott A, Russ L. Confidential Inquiry into premature deaths of people with learning disabilities (CIPOLD). Bristol: Norah Fry Research Centre. 2013.

10 Primary Care Domain, NHS Digital. Health and Care of People with Learning Disabilities: Experimental Statistics: 2016 to 2017 [Internet]. NHS Digital. NHS; 2017 [cited 2020Nov14]. Available from: https://digital.nhs.uk/data-and-information/publications/statistical/health-and-care-of-people-with-learning-disabilities/health-and-care-of-people-with-learning-disabilities-experimental-statistics-2016-to-2017

11 University of Bristol Norah Fry Centre for Disability Studies. The Learning Disabilities Mortality Review (LeDeR) Programme. Annual Report. London: Healthcare Improvement Quality Partnership, 2020.

12 Simpson S. Creating accessible healthcare environments for people with autism. Nursing Times. 2020 Jan;116(1):47-9.

13 National Patient Safety Agency (Great Britain). Understanding the patient safety issues for people with learning disabilities. National Patient Safety Agency; 2004.

14 Tuffrey-Wijne I, Goulding L, Gordon V, Abraham E, Giatras N, Edwards C, Gillard S, Hollins S. The challenges in monitoring and preventing patient safety incidents for people with intellectual disabilities in NHS acute hospitals: evidence from a mixed-methods study. BMC health services research. 2014 Dec;14(1):432.

For peer review only - http://bmjopen.bmj.com/site/about/guidelines.xhtml

15 Mimmo L, Harrison R, Hinchcliff R. Patient safety vulnerabilities for children with intellectual disability in hospital: a systematic review and narrative synthesis. BMJ paediatrics open. 2018;2(1).

16 Peters M, Godfrey C, McInerney P, Soares C, Khalil H, Parker D. The Joanna Briggs Institute reviewers' manual 2015: methodology for JBI scoping reviews.

17 Moher D, Stewart L, Shekelle P. All in the family: systematic reviews, rapid reviews, scoping reviews, realist reviews, and more. Syst Rev. 2015; 4: 183.

18 Arksey H, O'Malley L. Scoping studies: towards a methodological framework. International journal of social research methodology. 2005 Feb 1;8(1):19-32.

19 Levac D, Colquhoun H, O'Brien KK. Scoping studies: advancing the methodology. Implementation science. 2010 Dec;5(1):69.

20 Tricco AC, Lillie E, Zarin W, O'Brien KK, Colquhoun H, Levac D, Moher D, Peters MD, Horsley T, Weeks L, Hempel S. PRISMA extension for scoping reviews (PRISMA-ScR): checklist and explanation. Annals of internal medicine. 2018 Oct 2;169(7):467-73.

21 Welch V, Petticrew M, Tugwell P, Moher D, O'Neill J, Waters E, White H, PRISMA-Equity Bellagio group. PRISMA-Equity 2012 extension: reporting guidelines for systematic reviews with a focus on health equity. PLoS Med. 2012 Oct 30;9(10):e1001333.

22 Dunn K, Rydzewska E, Macintyre C, Rintoul J, Cooper SA. The prevalence and general health status of people with intellectual disabilities and autism co-occurring together: a total population study. Journal of Intellectual Disability Research. 2019 Apr;63(4):277-85.

23 O'Brien G, Pearson J. Autism and learning disability. Autism. 2004 Jun;8(2):125-40.

24 Mayes SD, Calhoun SL, Crowell EW. Learning disabilities and ADHD: Overlapping spectrum disorders. Journal of learning disabilities. 2000 Sep;33(5):417-24.

25 Iacono T, Bigby C, Unsworth C, Douglas J, Fitzpatrick P. A systematic review of hospital experiences of people with intellectual disability. BMC health services research. 2014 Dec 1;14(1):505.

26 Imms C, Adair B, Keen D, Ullenhag A, Rosenbaum P, Granlund M. 'Participation': a systematic review of language, definitions, and constructs used in intervention research with children with disabilities. Developmental Medicine & Child Neurology. 2016 Jan;58(1):29-38.

27 Panagioti M, Geraghty K, Johnson J, Zhou A, Panagopoulou E, Chew-Graham C, Peters D, Hodkinson A, Riley R, Esmail A. Association between physician burnout and patient safety, professionalism, and patient satisfaction: a systematic review and meta-analysis. JAMA internal medicine. 2018 Oct 1;178(10):1317-31.

28 Zegers M, Hesselink G, Geense W, Vincent C, Wollersheim H. Evidence-based interventions to reduce adverse events in hospitals: a systematic review of systematic reviews. BMJ open. 2016 Sep 1;6(9):e012555.

29 McGowan J, Sampson M, Salzwedel DM, Cogo E, Foerster V, Lefebvre C. PRESS peer review of electronic search strategies: 2015 guideline statement. Journal of clinical epidemiology. 2016 Jul 1;75:40-6.

For peer review only - http://bmjopen.bmj.com/site/about/guidelines.xhtml

30 Donaldson MS, Corrigan JM, Kohn LT, editors. To err is human: building a safer health system. National Academies Press; 2000 Apr 1.

31 Popay J, Roberts H, Sowden A, Petticrew M, Arai L, Rodgers M, Britten N, Roen K, Duffy S. Guidance on the conduct of narrative synthesis in systematic reviews. A product from the ESRC methods programme Version. 2006 Apr 1;1:b92.

32 Campbell M, Katikireddi SV, Sowden A, McKenzie JE, Thomson H. Improving Conduct and Reporting of Narrative Synthesis of Quantitative Data (ICONS-Quant): protocol for a mixed methods study to develop a reporting guideline. BMJ open. 2018 Feb 1;8(2):e020064.

33 Best KM, Asaro LA, Curley MA, Wypij D, Allen GL, Angus DC, Ascenzi JA, Bateman ST, Borasino S, Bowens CD, Bysani GK. Sedation Management for Critically Ill Children with Pre-Existing Cognitive Impairment. The Journal of pediatrics. 2019 Mar 1;206:204-11.

34 Hemsley B, Georgiou A, Hill S, Rollo M, Steel J, Balandin S. An integrative review of patient safety in studies on the care and safety of patients with communication disabilities in hospital. Patient education and counseling. 2016 Apr 1;99(4):501-11.

35 Hemsley B, Steel J, Worrall L, Hill S, Bryant L, Johnston L, Georgiou A, Balandin S. A systematic review of falls in hospital for patients with communication disability: highlighting an invisible population. Journal of safety research. 2019 Feb 1;68:89-105.

36 Kelly CL, Thomson K, Wagner AP, Waters JP, Thompson A, Jones S, Holland AJ, Redley M. Investigating the widely held belief that men and women with learning disabilities receive poor quality healthcare when admitted to hospital: a single-site study of 30-day readmission rates. Journal of Intellectual Disability Research. 2015 Sep;59(9):835-44.

37 Shah N, Al-Ahmari A, Al-Yamani A, Dupuis L, Stephens D, Hitzler J. Outcome and toxicity of chemotherapy for acute lymphoblastic leukemia in children with Down syndrome. Pediatric blood & cancer. 2009 Jan;52(1):14-9.

38 Oulton K, Gibson F, Carr L, Hassiotis A, Jewitt C, Kenten C, Russell J, Whiting M, Tuffrey-Wijne I, Wray J. Mapping staff perspectives towards the delivery of hospital care for children and young people with and without learning disabilities in England: a mixed methods national study. BMC health services research. 2018 Dec 1;18(1):203.

39 Royal Society for Mentally Handicapped Children and Adults. Treat Me Right!: Better Healthcare for People with Learning Disability. Mencap; 2004.

40 Royal Society for Mentally Handicapped Children and Adults. Death by indifference: Following up the Treat Me Right! report. Mencap; 2007.

41 Michael J. Healthcare for all: report of the independent inquiry into access to healthcare for people with learning disabilities. Independent Inquiry into Access to Healthcare for People with Learning Disabilities; 2008.

42 Local Government Ombudsman. Six Lives: The Provision of Public Services to People with Learning Disabilities. The Stationery Office London. 2009.

43 Department of Health. Six lives: Progress report on healthcare for people with learning disabilities. Department of Health; 2013.

For peer review only - http://bmjopen.bmj.com/site/about/guidelines.xhtml

44 Mencap. Hear my voice: healthcare. 2020. https://www.mencap.org.uk/get-involved/campaign-mencap/hear-my-voice/hear-my-voice-healthcare

45 University of Bristol Norah Fry Centre for Disability Studies. The Learning Disabilities Mortality Review (LeDeR) Programme. Annual Report. London: Healthcare Improvement Quality Partnership, 2016.

46 University of Bristol Norah Fry Centre for Disability Studies. The Learning Disabilities Mortality Review (LeDeR) Programme. Annual Report. London: Healthcare Improvement Quality Partnership, 2017.

47 University of Bristol Norah Fry Centre for Disability Studies. The Learning Disabilities Mortality Review (LeDeR) Programme. Annual Report. London: Healthcare Improvement Quality Partnership, 2018.

48 McGowan P. Ask Listen Do: Oliver's Story [Internet]. NHS Choices. NHS; 2018 [cited 2020Nov15]. Available from: https://www.england.nhs.uk/blog/ask-listen-do-olivers-story/

49 NHS England, NHS Improvement. The NHS patient safety strategy: safer culture, safer systems, safer patients. 2019. https://www.england.nhs.uk/wp-content/uploads/2020/08/190708_Patient_Safety_Strategy_for_website_v4.pdf

50 INTERIM BULLETIN UNDIAGNOSED CARDIOMYOPATHY IN A YOUNG PERSON WITH AUTISM. Healthcare Safety Investigation Branch. 2019. https://www.hsib.org.uk/documents/139/hsib_interim_bulletin_undiagnosed_cardiomyopathy_autism.pdf

51 Care Quality Commission. Declare Your Care: People with learning disabilities. 2020. https://www.cqc.org.uk/help-advice/your-stories/declare-your-care-people-learning-disabilities

52 National Institute for Health Research. Better health and care for all. 2020. http://dx.doi.org/10.3310/themedreview-04326

53 Hemsley B, Balandin S, Worrall L. Nursing the patient with developmental disability in hospital: Roles of paid carers. Qualitative Health Research. 2011 Dec;21(12):1632-42.

54 Mimmo L, Woolfenden S, Travaglia J, Harrison R. Partnerships for safe care: A meta-narrative of the experience for the parent of a child with Intellectual Disability in hospital. Health Expectations. 2019 Dec;22(6):1199-212.

55 Redley M, Lancaster I, Pitt A, Holland A, Thompson A, Bradley JR, Glover G, Thomson K, Jones S, Herbert B, Holme A. "Reasonable adjustments" under the UK's Equality Act 2010: An enquiry into the care and treatment to patients with intellectual disabilities in acute hospital settings. Journal of Applied Research in Intellectual Disabilities. 2019 Nov;32(6):1412-20.

56 Tuffrey-Wijne I, Abraham E, Goulding L, Giatras N, Edwards C, Gillard S, Hollins S. Role confusion as a barrier to effective carer involvement for people with intellectual disabilities in acute hospitals: findings from a mixed-method study. Journal of advanced nursing. 2016 Nov;72(11):2907-22.

57 Piper E. Investigating the experiences of people with learning disabilities in Accident & Emergency from a carer perspective [Doctor of Clinical Psychology]. University of Hull; 2008.

For peer review only - http://bmjopen.bmj.com/site/about/guidelines.xhtml

58 Bradbury-Jones C, Rattray J, Jones M, MacGillivray S. Promoting the health, safety and welfare of adults with learning disabilities in acute care settings: a structured literature review. Journal of Clinical Nursing. 2013 Jun;22(11-12):1497-509.
59 Brittle R. Managing the needs of people who have a learning disability. Nursing times. 2004;100(10):28-9.

60 Hemsley B, Balandin S. A metasynthesis of patient-provider communication in hospital for patients with severe communication disabilities: informing new translational research. Augmentative and Alternative Communication. 2014 Dec 1;30(4):329-43.

61 Jolly AA. Handle with Care: Top Ten Tips a Nurse Should Know Before Caring For a Hospitalized Child with Autism Spectrum Disorder. Pediatric nursing. 2015 Jan 1;41(1).

62 Kopecky K, Broder-Fingert S, Iannuzzi D, Connors S. The needs of hospitalized patients with autism spectrum disorders: a parent survey. Clinical Pediatrics. 2013 Jul;52(7):652-60.

63 Wilkinson S. Assessing the perioperative communication needs of a patient with learning disabilities: An holistic case study approach. Journal of perioperative practice. 2018 Oct;28(10):278-82.

64 NHS. The NHS long term plan. 2019. https://www.longtermplan.nhs.uk/

65 Department of Health and Social Care. Consultation outcome: Learning disability and autism training for health and care staff. 2019. https://www.gov.uk/government/consultations/learning-disability-and-autism-training-for-health-and-care-staff

66 Brown HK, Kirkham YA, Cobigo V, Lunsky Y, Vigod SN. Labour and delivery interventions in women with intellectual and developmental disabilities: a population-based cohort study. J Epidemiol Community Health. 2016 Mar 1;70(3):238-44.

67 Mitra M, Parish SL, Akobirshoev I, Rosenthal E, Simas TA. Postpartum hospital utilization among Massachusetts women with intellectual and developmental disabilities: a retrospective cohort study. Maternal and child health journal. 2018 Oct 1;22(10):1492-501.

68 Parish SL, Mitra M, Son E, Bonardi A, Swoboda PT, Igdalsky L. Pregnancy outcomes among US women with intellectual and developmental disabilities. American journal on intellectual and developmental disabilities. 2015 Sep;120(5):433-43.

69 McConnell D, Mayes R, Llewellyn G. Women with intellectual disability at risk of adverse pregnancy and birth outcomes. Journal of Intellectual Disability Research. 2008 Jun;52(6):529-35.

70 Poulton AS, Armstrong B, Nanan RK. Perinatal outcomes of women diagnosed with attention-deficit/hyperactivity disorder: an Australian Population-Based Cohort Study. CNS drugs. 2018 Apr 1;32(4):377-86.

71 Change and PEN. Hidden Voices of Maternity Parents With Learning Disabilities Speak Out. 2015. https://patientexperiencenetwork.org/wp-content/uploads/2019/10/Hidden-Voices-of-Maternity-Executive-Summary-FINAL-260815-2.pdf

For peer review only - http://bmjopen.bmj.com/site/about/guidelines.xhtml

72 Bartz-Kurycki MA, Anderson KT, Austin MT, Kao LS, Tsao K, Lally KP, Kawaguchi AL. Increased complications in pediatric surgery are associated with comorbidities and not with Down syndrome itself. Journal of Surgical Research. 2018 Oct 1;230:125-30.

73 Boylan MR, Kapadia BH, Issa K, Perfetti DC, Maheshwari AV, Mont MA. Down syndrome increases the risk of short-term complications after total hip arthroplasty. The Journal of arthroplasty. 2016 Feb 1;31(2):368-72.

74 Evans JM, Dharmar M, Meierhenry E, Marcin JP, Raff GW. Association between Down syndrome and in-hospital death among children undergoing surgery for congenital heart disease: a US population-based study. Circulation: Cardiovascular Quality and Outcomes. 2014 May;7(3):445-52.

75 Lin JA, Liao CC, Chang CC, Chang H, Chen TL. Postoperative adverse outcomes in intellectually disabled surgical patients: a nationwide population-based study. PLoS One. 2011 Oct 27;6(10):e26977.

76 Purifoy ET, Spray BJ, Riley JS, Prodhan P, Bolin EH. Effect of Trisomy 21 on Postoperative Length of Stay and Non-cardiac Surgery After Complete Repair of Tetralogy of Fallot. Pediatric Cardiology. 2019 Dec 1;40(8):1627-32.

77 Tóth R, Szántó P, Prodán Z, Lex DJ, Sápi E, Szatmári A, Gál J, Szántó T, Székely A. Down syndrome and postoperative complications after paediatric cardiac surgery: a propensity-matched analysis. Interactive cardiovascular and thoracic surgery. 2013 Oct 1;17(4):691-7.

78 Louis JD, Jodhka U, Jacobs JP, He X, Hill KD, Pasquali SK, Jacobs ML. Contemporary outcomes of complete atrioventricular septal defect repair: analysis of the Society of Thoracic Surgeons Congenital Heart Surgery Database. The Journal of thoracic and cardiovascular surgery. 2014 Dec 1;148(6):2526-31.

79 Pugely AJ, Martin CT, Gao Y, Ilgenfritz R, Weinstein SL. The incidence and risk factors for short-term morbidity and mortality in pediatric deformity spinal surgery: an analysis of the NSQIP pediatric database. Spine. 2014 Jul 1;39(15):1225-34.

80 Vervloessem D, van Leersum F, Boer D, Hop WC, Escher JC, Madern GC, de Ridder L, Bax KN. Percutaneous endoscopic gastrostomy (PEG) in children is not a minor procedure: risk factors for major complications. InSeminars in pediatric surgery 2009 May 1 (Vol. 18, No. 2, pp. 93-97). WB Saunders.

81 Flood B. Safety of People with Intellectual Disabilities in Hospital. What Can the Hospital Pharmacist Do to Improve Quality of Care?. Pharmacy. 2017 Sep;5(3):44.

82 Friese T, Ailey S. Specific standards of care for adults with intellectual disabilities. Nursing Management. 2015 Mar 25;22(1).

83 Glasby AM. Meeting the needs of people with learning disabilities in acute care. British journal of nursing. 2002 Nov 28;11(21):1389-92.

84 Lewanda AF, Matisoff A, Revenis M, Harahsheh A, Futterman C, Nino G, Greenberg J, Myseros JS, Rosenbaum KN, Summar M. Preoperative evaluation and comprehensive risk assessment for children with D own syndrome. Pediatric Anesthesia. 2016 Apr;26(4):356-62.

For peer review only - http://bmjopen.bmj.com/site/about/guidelines.xhtml

85 Northway R, Rees S, Davies M, Williams S. Hospital passports, patient safety and person-centred care: A review of documents currently used for people with intellectual disabilities in the UK. Journal of clinical nursing. 2017 Dec;26(23-24):5160-8.

86 Read S, Johnson T. Rapid risk assessment in acute hospital for patients with intellectual disabilities. Advances in Mental Health and Intellectual Disabilities. 2012 Nov 16;6(6):321-32.

87 Vlassakova BG, Emmanouil DE. Perioperative considerations in children with autism spectrum disorder. Current opinion in anaesthesiology. 2016 Jun 1;29(3):359-66.
88 Kent A. Improving acute care of people with learning disabilities. Nursing Times. 2008 Feb 5;104(5):32-3.

89 Royal College of Nursing. Dignity in health care for people with learning disabilities. 2009. http://oxleas.nhs.uk/site-media/cms-downloads/RCN_Dignity_in_healthcare.pdf

90 Guidelines and Audit Implementation Network. Guidelines on Caring For People with a Learning Disability in General Hospital Setting. 2010. https://rqia.org.uk/RQIA/files/81/81662c46-b7bb-43a5-9496-a7f2d919c2a3.pdf

91 The Hillingdon Hospitals NHS Foundation Trust. Responding to the needs of people with learning disabilities [Internet]; [cited 2020Nov16]. Available from: https://www.thh.nhs.uk/about/safety/learning_dis.php

92 Ailey S, Hart R. Comprehensive Program to Support Patients and Staff Improves Hospital Experience for Adult Patients With Intellectual and Developmental Disabilities [Internet]. AHRQ Innovations Exchange: Innovations and Tools to Improve Quality and Reduce Disparities. Agency for Healthcare Research and Quality; 2010 [cited 2020Nov16]. Available from: https://www.innovations.ahrq.gov/profiles/comprehensive-program-support-patients-and-staff-improves-hospital-experience-adult

93 Public Health Nursing Division, Department of Health in partnership with the Modernising Learning Disabilities Nursing UK Implementation Group. Strengthening the Commitment: One year on: Progress report on the UK Modernising Learning Disabilities Nursing Review. 2014. https://assets.publishing.service.gov.uk/government/uploads/system/uploads/attachment_data/file/309153/Strengthening_the_commitment_one_year_on_published.pdf

94 Bowness B. Improving general hospital care of patients who have a learning disability. 2014. http://www.1000livesplus.wales.nhs.uk/sitesplus/documents/1011/How to %2822%29 Learning Disabilites Care Bundle web.pdf

95 Blair J. Everybody's life has worth – Getting it right in hospital for people with an intellectual disability and reducing clinical risks [Internet]. University of Hertfordshire; 2014 [cited 2020Nov16]. Available from: http://www.intellectualdisability.info/how-to-guides/articles/everybodys-life-has-worth-getting-it-right-in-hospital-for-people-with-an-intellectual-disability-and-reducing-clinical-risks

96 Public Health England. Working together 2: Easy steps to improve support for people with learning disabilities in hospital Guidance for hospitals, families and paid support staff. 2014. http://cdn.basw.co.uk/upload/basw_14709-8.pdf

For peer review only - http://bmjopen.bmj.com/site/about/guidelines.xhtml

97 National Quality Board. National Guidance on Learning from Deaths. A Framework for NHS Trusts and NHS Foundation Trusts on Identifying, Reporting, Investigating and Learning from Deaths in Care. 2017. https://www.england.nhs.uk/wp-content/uploads/2017/03/nqb-national-guidance-learning-from-deaths.pdf

98 NHS England. Ask Listen Do Resource for Organisations and Practitioners. 2018. https://www.england.nhs.uk/wp-content/uploads/2018/10/ask-listen-do-for-organisations-and-practitioners-v1.pdf

99 Mencap and The National Autistic Society: Treat me well campaign. The Oliver McGowan mandatory training in learning disability and autism [Internet]. Mencap; 2018 [cited 2020Nov16]. Available from: https://www.mencap.org.uk/get-involved/campaign-mencap/treat-me-well/announcement-learning-disability-training-health-and

100 NHS Improvement. The learning disability improvement standards for NHS trusts. 2018. https://www.england.nhs.uk/wpcontent/uploads/2020/08/v1.17_Improvement_Standards_added_note.pdf

101 The Westminster Commission on Autism. A Spectrum of Obstacles An Inquiry into Access to Healthcare for Autistic People. 2016. https://westminsterautismcommission.files.wordpress.com/2016/03/ar1011_ncg-autism-report-july-2016.pdf

102 Fylan B, Marques I, Ismail H, Breen L, Gardner P, Armitage G, Blenkinsopp A. Gaps, traps, bridges and props: a mixed-methods study of resilience in the medicines management system for patients with heart failure at hospital discharge. BMJ open. 2019 Feb 1;9(2):e023440.

103 O'Hara JK, Aase K, Waring J. Scaffolding our systems? Patients and families 'reaching in'as a source of healthcare resilience. BMJ Qual Saf. 2019; 28 (1): 3–6.

104 O'Hara JK, Baxter R, Hardicre N. 'Handing over to the patient': A FRAM analysis of transitional care combining multiple stakeholder perspectives. Applied Ergonomics. 2020 May 1;85:103060.

For peer review only - http://bmjopen.bmj.com/site/about/guidelines.xhtml

For peer review only

Figure 1. PRISMA flow diagram.

For peer review only - http://bmjopen.bmj.com/site/about/guidelines.xhtml

For peer review only

For peer review only - http://bmjopen.bmj.com/site/about/guidelines.xhtml

For peer review only

For peer review only - http://bmjopen.bmj.com/site/about/guidelines.xhtml

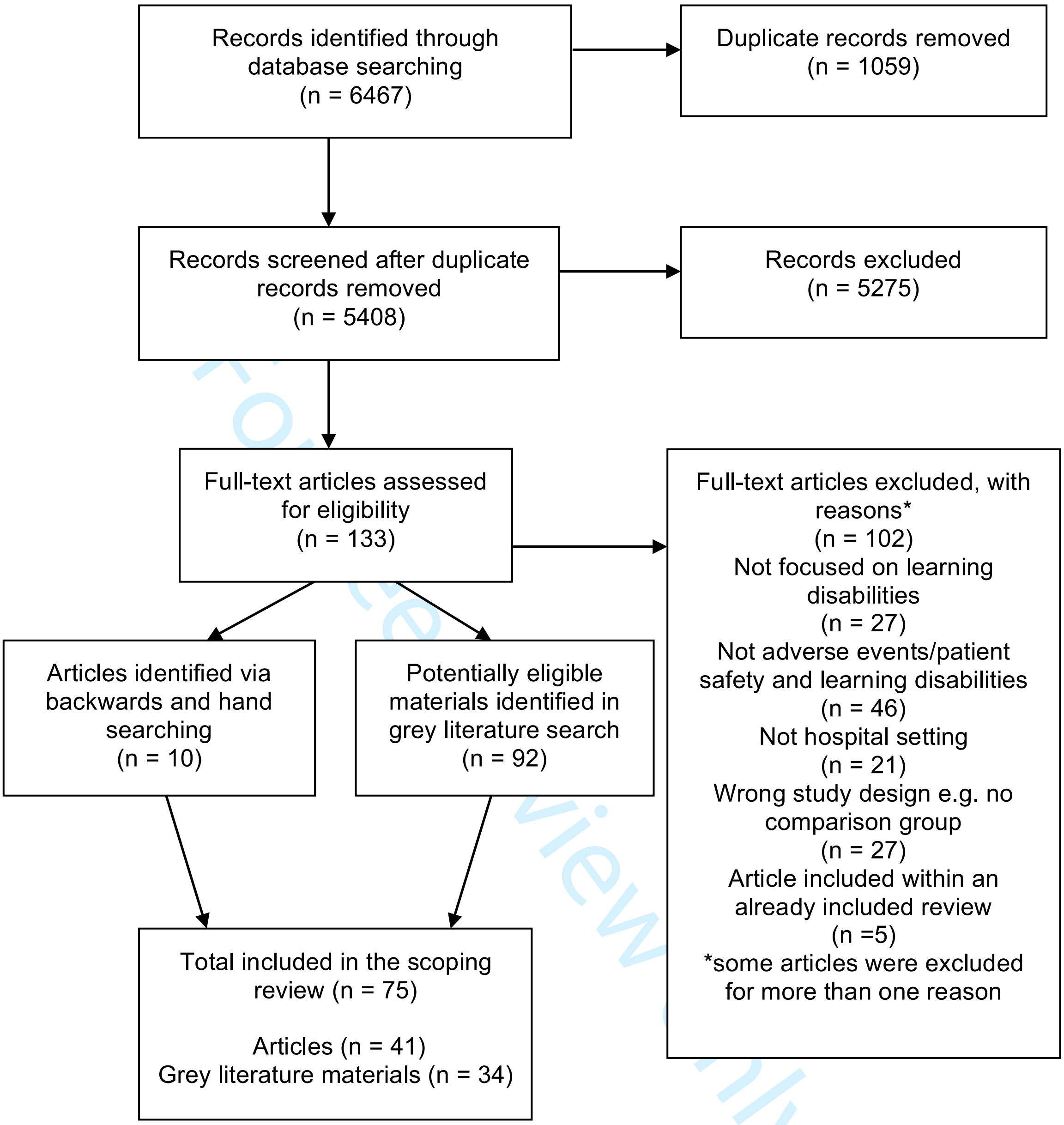
Records identified through database searching (n = 6467)
Duplicate records removed (n = 1059)
Records screened after duplicate records removed (n = 5408)
Records excluded (n = 5275)
Full-text articles assessed for eligibility (n = 133)
Full-text articles excluded, with reasons* (n = 102)
Not focused on learning disabilities (n = 27)
Not adverse events/patient safety and learning disabilities (n = 46)
Not hospital setting (n = 21)
Wrong study design e.g. no comparison group (n = 27)
Article included within an already included review (n =5)
*some articles were excluded for more than one reason
Articles identified via backwards and hand searching (n = 10)
Potentially eligible materials identified in grey literature search (n = 92)
Total included in the scoping review (n = 75)
Articles (n = 41)
Grey literature materials (n = 34)

Appendix 1 MEDLINE search
Total once population, concept and context combined = 4087
Restricted to year 2000 onwards and English Language only = 3355

Search terms	Results
Learning disability (Population)	
Intellectual disabilit* .mp.	36221
Statemented .mp.	1
Communication disorder*1 .mp.	2692
Communication impairment*1 .mp.	502
Complex communication needs .mp.	165
Intellectual* handicap* .mp.	99
Intellectual impairment*1 .mp.	1072
Learning difficult* .mp.	1793
Non?speaking .mp.	58
Mental handicap .mp.	551
Asperger*1 .mp.	2424
Down syndrome .mp.	20162
Complex need*1 .mp.	1087
Autis* .mp.	37553
Dyslexi* .mp.	7078
Different need*1 .mp.	606
Altered need*1 .mp.	9
Sensory impair* .mp.	1594
ADHD .mp.	19740
Attention deficit .mp.	32081
Hyperact* .mp.	53234
Global development delay .mp.	21
Mental retardation .mp.	20971
Cognition disorder* .mp.	60219
Learning disorder* .mp.	10138
Developmental disabilit* .mp.	18873
Cognitive disabilit* .mp.	890
Cognitive impairment*1 .mp.	48628
Communication disabilit* .mp.	154
Adverse events (Concept)	
Adverse event*1 .mp.	123116
Sentinel event*1 .mp.	768
Near miss*2 .mp.	1726
Close call*1 .mp.	126
Critical outcome*1 .mp.	217
Adverse outcome*1.mp.	21887
Safety event*1 .mp.	741
Never event*1 .mp.	284
Serious incident*1 .mp.	139
Untoward incident*1 .mp.	45
Clinical incident*1 .mp.	163
Incident report*1 .mp.	911
Patient safety incident*1.mp.	317
Safety incident*1 .mp.	490
Patient safety (Concept)	
Iatrogenic disease* .mp.	35039
Medical error .mp.	10808
Patient safety.mp.	1285
Human error*.mp.	1693
((adverse or avoidable or preventable or unsafe or safet*) ADJ2 (event* or outcome* or complication* or death* or effect* or reaction* or accident* or injur*)) .mp.	1598108

For peer review only

((medica* or diagnostic or therapeautic or administration or dispensing or prescri*) ADJ2 (error* or mistake* or fault*)) .mp.	56303
(patient* ADJ2 (risk* or incident* or accident* or harm*)) .mp.	99086
Near miss* .mp.	1733
Never event*.mp.	285
Untoward incident* .mp.	45
Serious incident* .mp.	145
Serious report* event* .mp.	16
Medical error*1 .mp.	17806
Iatrogenic .mp.	25461
Hospital (Context)	
Hospital*1 .mp.	937110
Acute care .mp.	16563
Secondary care .mp.	5211
Tertiary care unit*1 .mp.	115
Ward*1 .mp.	40623
Department*1 .mp.	238101
In?patient*1 .mp.	88189
Out?patient*1 .mp.	132903
Triage*1 .mp.	17878
Intensive care .mp.	129300
Critical care .mp.	54161
Urgent care .mp.	1544
Internal medicine .mp.	21071
A&E .mp.	17067
Accident and emergency .mp.	3744
Emergency care .mp.	6633
Emergency medicine .mp.	18626
Emergency treatment .mp.	12279
Emergency admission .mp.	741
Hospitali#ation .mp.	157081
Ambulatory care .mp.	46042
Perioperative care .mp.	15315
Preoperative care .mp.	44087
Hospitali#ed .mp.	82540
Perioperative .mp.	79749
Preoperative .mp.	207609
Postoperative .mp.	556109
Re?operative .mp.	1720
Post?operative .mp.	556113
Admission .mp.	148583
Casualty .mp.	5064
Discharge .mp.	140976
Emergency department .mp.	61318

For peer review only

Appendix 2 Grey literature publicly available material searched

Mencap, NHS England, NHS Improvement, Healthcare Quality Improvement Partnership (HQIP), Healthcare Safety Investigation Branch (HSIB), The Kings Fund, Agency for Healthcare Research and Quality (AHRQ), Care Quality Commission (CQC), The Health Foundation, World Health Organisation, Institute of Healthcare improvement, Collaborations for Leadership in Applied Health Research and Care (CLAHRC), Nuffield Trust, Public Health England, The Office for National Statistics, The National Institute for Clinical Excellence, Mind, Learning Disability England, the British Institute of Learning Disabilities (BILD), Learning Disability Practice – RCNi, The National Research Center on Learning Disabilities (NRCLD), Royal College of Nursing, Royal College of Midwives, Nursing & Midwifery Council, Faculty of Dental Surgery, Faculty of Intensive Care Medicine,
Royal College of Anaesthetists, Royal College of Emergency Medicine, Royal College of Obstetricians and Gynaecologists, Royal College of Paediatrics and Child Health, Royal College of Physicians of Edinburgh, Royal College of Physicians of Ireland, Royal College of Physicians of London, Royal College of Physicians and Surgeons of Glasgow, Royal College of Surgeons in Ireland, Royal College of Surgeons of Edinburgh, Royal College of Surgeons of England.

For peer review only

Online Supplementary Table 1. Included articles with author, year, country, aims, participants, methodology and key findings (presented in alphabetical order)

Author	Year	Country	Aims	Participants	Methodology	Key findings
Bartz-Kurycki et al[72]	2018	USA	Investigate whether DS is a risk factor for postoperative complications in paediatric patients undergoing gastrointestinal and non-cardiac thoracic surgery, and determine factors associated with complications	Total: 91,478 patients <18y old who underwent gastrointestinal or non-cardiac thoracic surgery. With DS: 1,476 (1.6%)	Quantitative: Retrospective cohort study using univariate analysis and multivariate logistic regression	DS patients had significantly higher postoperative complication rates than controls. However, comorbidities rather than DS were a greater risk factor for complications
Best, Asaro and Curley[33]	2019	USA	Describe, compare and evaluate indicators of the efficacy of analgesia and sedation management for critically ill children with and without CI	Total: 2,449 patients 2 weeks – 17y old were included in who underwent invasive mechanical ventilation for acute airway and/or parenchymal lung disease<br>With CI: 412	Quantitative: Retrospective cohort study using linear, cumulative logit, logistic, multinomial logistic, proportional hazards, and Poisson regression and chi-squared	CI patients received significantly lower doses of analgesia and sedation medication than those without CI. However, it was unclear if this was due to lower requirements or vulnerabilities to inadequate assessment
Boylan et al[73]	2016	USA	Assess and compare short-term outcomes of total hip arthroplasty in patients with and without DS	Total: 543,085 patients who underwent total hip arthroplasty in 1998 – 2010.<br>With DS: 241 (0.04%)	Quantitative: Retrospective cohort study using logistic regression modelling, independent sample T-tests and linear regression	DS patients had significantly increased rates of medical, surgical and any complication compared to matched controls. This included pneumonia, urinary tract infection and wound haemorrhage. Patients with DS also had a longer mean length of stay
Bradbury-Jones et al[58]	2013	UK	Review evidence regarding the promotion of health, safety and welfare of adults with LD in acute care	Studies focussed on adults with LDs	Literature review	Six areas of influence on the health, safety and welfare of adults with LDs in acute hospitals were identified: care provision, communication,

For peer review only

						staff attitudes, staff knowledge, supporters and carers and physical environment
Brittle[59]	2004	UK	Consider how nurses may deal with increasing numbers of LD patients accessing generic health services including hospitals	Studies including people with LDs accessing generic health services	Literature review	To ensure that nurses do as much as possible to recognise risk when caring for people with LDs, they must; recognise any prejudices and overcome them, acknowledge that people with LDs have the same rights to healthcare as others, develop further understanding of LDs and collaborate with carers and professionals
Brown et al[66]	2016	Canada	Compare the occurrence of labour induction, c-section and operative vaginal delivery in women with and without IDD and determine whether pre-pregnancy health conditions or pregnancy complications explain any differences	Total: 386,706 deliveries to 263,284 women<br>With IDD: 3,932 deliveries to 2,584 women<br>Without IDD: 382,774 deliveries to 260,700 women	Quantitative: Retrospective cohort study using Poisson regression and mediation analysis	Women with IDD were younger, lived in lower income areas and had higher rates of pre-pregnancy health conditions including: pre-existing diabetes mellitus, herpes or HIV, epilepsy, and psychiatric disorders. They had higher rates of complications such as pre-eclampsia and fetal death, and more likely to have labour interventions such as induction and caesarean. Psychiatric disorders were the most important variable for labour induction and caesarean
Evans et al[74]	2014	USA	Identify differences in in-hospital	Total: 51,309 patients	Quantitative:	When controlling for risk

For peer review only - http://bmjopen.bmj.com/site/about/guidelines.xhtml

			mortality after cardiac surgery in paediatric patients with and without DS	<18y old who underwent surgery to correct congenital heart disease	Retrospective cohort study using multivariable logistic regression analysis	category, premature birth, presence of ≥1 major non-cardiac structural anomalies, and age, a diagnosis of DS was associated with a lower odds of in-hospital death
Flood[81]	2017	UK	Raise awareness of how hospital pharmacists can contribute to safety when supporting people with ID in hospital	Discussing studies and policies focussing on people with ID in hospital	Commentary	To help pharmacists ensure people with IDs receive reasonably adjusted quality care it is important that; pharmacists know that a patient has IDs, pharmacy staff are aware of general healthcare and specific medication-related issues, transitions of care are considered as they are particularly vulnerable for people with IDs and people with IDs require equitable care that is appropriate for their needs
Friese and Ailey[82]	2015	USA	Develop care plans and an educational module for nurses caring for patients with LDs	Nurses completed a pre-evaluation (n = 75) and post-evaluation (n = 99) questionnaire. Over 300 nurses completed the educational module	Mixed methods comprising: Literature review and PDSA cycles to develop care plans and educational module. Quantitative assessment of nurses' confidence in caring for patients with LD after completing the module using Chi squared	Key components of care plans were communication, a safe environment, enhancing patients' behaviour and cooperation with care, and carer involvement. Nurse educational module aimed to increase understanding of needs of LD patients, improve communication and prevent adverse events. After completing the education module analysis showed

For peer review only

						significant improvement in nurses' confidence when caring for patients with LDs
Glasby[83]	2002	UK	Explore how a specialist LD team aimed to improve patient care for those with LDs	A specialist learning disability team of nursing and support staff set up to improve care for people with LDs	Improvement project: Observational	Core tasks of LD team included: accompanying individuals to appointments, ensuring individuals understands what is going to happen in hospital, considering consent issues, liaising with wards to help them understand the person's needs, providing practical support and advocating for the person's needs in hospital, enabling carers to have a break, facilitating community support before discharge, following up after discharge to ensure that all needs are being met, educating acute staff and developing training materials for staff and trainees
Hemsley, Balandin and Worrall[53]	2011	Australia	Determine how paid carers, adults with developmental disability and CCNs and hospital nurses view paid carers' roles in supporting adults with developmental disability and CCNs in hospital	Total: 45 participants<br>Adults with developmental disability and CCNs: 15<br>Paid carers: 15<br>Hospital nurses: 15	Qualitative: Interview study using narrative analysis	Paid carers volunteer informal hours in the acute hospital setting, motivated by perceived responsibility for safety, well-being and communication needs in hospital. Their role can be confused and blurred with both nursing and family carer roles

For peer review only

Hemsley and Balandin[60]	2014	Australia	Review and map the evidence regarding communication in hospital for patients with severe communication disabilities to propose evidence-based core strategies to improve communication in hospital, and to propose a translational research agenda	Studies including people with lifelong disability and CCNs	Mixed methods literature review: Qualitative meta-synthesis and quantitative meta-analysis	The reviewed highlighted the importance of; poor inter agency communication (requires coordination to help stakeholders navigate the complex system), devoting time to communication (value individuals who take time to speak directly to the patient), ensure access to communication tools and aids, access to personally held written health information and collaborate effectively with carers
Hemsley et al[34]	2016	Australia	Identify research reports regarding investigating the care or safety of adults with communication disabilities in hospital, and to analyse findings according to the generic model of patient safety	Studies including hospital patients with communication disabilities	Literature review	Patient safety incident and adverse event reporting lacked detail e.g. little demographic, descriptive, temporal and categorical information about the patient and staff and how events were detected. Successful advocacy affected outcomes, although where advocacy was ignored outcomes were worse. Stories of adverse events themes included; suffering, isolation due to not having a method to communicate with nurses, a perilous care situation culminating in an adverse event and protective carers discovering or

For peer review only

						forestalling an adverse event
Hemsley et al[35]	2019	Australia	Examine evidence to guide future research on falls in adult hospital patients with communication disability, identify contributory factors to falls, increase awareness of those with potential additional risk for falling and inform hospital policies on measures needed to reduce falls	Studies including adults with communication disabilities	Literature review	Varied terminology used to describe communication disabilities. Diagnosis included as a falls risk factor in some studies. Most studies found communication disability to be related to falls (to both increase and decrease risk) but did not elaborate upon the finding, making an important omission to knowledge contribution
Iacono et al[25]	2014	Australia	Evaluate the evidence regarding how hospital systems respond to adults with ID, their families and carers	Studies focussed on people with ID or carers or staff perceptions and experiences of quality of care of people with ID	Literature review: Systematic and meta-synthesis	Seven themes including; individual fear of hospital encounters, reliance on paid and family carers for basic needs and advocacy, responsibilities and staff knowledge, skills and attitudes. Despite repeated calls for staff training, in one recent study staff themselves identified their lack of knowledge and need for ID training
Jolly[61]	2015	USA	Familiarise the paediatric nurse with autism and create a resource for successful inpatient treatment of a child with the disorder	Discussing knowledge of working with children with autism	Commentary	Themes included; understanding autism, encouraging family involvement, best way of communicating, change is a challenge for children with autism, consistent caregivers, safe environment, emotional triggers and reward systems,

						and MDT from admission.
Kelly et al[36]	2015	UK	Compare 30-day hospital readmission rates of people with and without LDs	Total: 66,870 patients<br>With LDs: 256	Quantitative: Retrospective cohort study using chi-squared	No significant difference in 30-day readmission rates for patients with and without LDs. However, 69% of readmissions of those with LDs were potentially preventable. Those with more profound LDs were at greater risk of experiencing poor quality care and experiencing readmission within 30 days, and this group comprised over half of the PPRs
Kopecky et al[62]	2013	USA	Characterise the needs of hospital patients with autism in various categories via a survey to aid facilitating the inpatient experience and improve quality of care	80 parents and guardians of patients with autism	Mixed methods: Survey comprising qualitative and quantitative items	Those with autism have a wide range of challenges and needs including communication, social/pragmatic concerns, and sensory processing while in the hospital. An individualised approach must be used to assess and accommodate needs. 50% of parents had safety concerns for their child's hospital stay including; wandering or elopement, self-injury or aggression, pica, and pulling out IV tubing
Lewanda et al[84]	2016	USA	Optimise patient safety for children with DS by choosing the most appropriate setting and perioperative personnel, and to mitigate those risk	Specialist team presenting their knowledge on working with children with DS	Commentary	Pre-surgical evaluations for children with DS should identify appropriate personal and equipment and focus on; combining 2+ compatible

For peer review only

			factors amenable to intervention			surgical procedures under one anaesthesia event, assessing for undiagnosed or residual heart disease and the presence of pulmonary hypertension, considering potential cervical spine instability, assess if patient is taking dietary supplements and having various options available for anaesthesia during surgery
Lin et al[75]	2011	Taiwan	Clarify whether ID is an independent risk factor for in-hospital major surgeries, and to validate the postoperative adverse outcomes in patients with ID	Total: 3,983 patients with preoperative diagnosis of ID who underwent inpatient major surgeries in 2004 - 2007	Quantitative: Cohort study using descriptive statistics and multivariate logistic regression	Surgical patients with ID had significantly higher incidence of preoperative comorbidities and postoperative complications while consuming more medical resources than controls. They also had higher average length of hospital stay and use of intensive care, as well as higher rates of acute renal failure, pneumonia, postoperative bleeding septicaemia, stroke and any complications. Postoperative 30-day mortality showed no significant difference between patients with or without ID after adjusting teaching hospital, low income, urbanization and coexisting disease
McConnell,	2008	Australia	Explore the prevalence of poor	Total: 834 women who	Quantitative: Cohort	Pregnant women with ID

Mayes and Llewellyn[69]			pregnancy and birth outcomes in women with ID and/or self-reported learning difficulties in an antenatal population	gave birth to 839 children.<br>With ID and/or self-reported learning difficulties: 54<br>Without ID and/or self-reported learning difficulties: 785	study using descriptive statistics	and/or self-reported learning difficulties had significantly higher prevalence of pre-eclampsia than those without. There were also higher rates of pre-term delivery and low birth weight, but this was not statistically significant
Mimmo et al[54]	2019	Australia	Identify evidence regarding the parental experience of hospitalisation with a child with ID and care quality and safety	Studies focussed on parents or carers of children who are inpatients with ID	Literature review: scoping review and meta-synthesis	Findings from 11 studies were consolidated into five themes; being more than a parent, importance of role negotiation to reduce ambiguity about the role of the parent, building trust and relationships through effective communication, the cumulative effect of previous experiences of hospitalisation and healthcare staff taking time to know the child as an individual. Partnerships in care are vital to deliver safe care for children with ID
Mimmo, Harrison and Hinchcliff[15]	2018	Australia	Narratively synthesise evidence concerning the experience of iatrogenic harm during hospitalisation for children with ID	Studies focussed on child inpatients' with ID	Literature review: systematic using narrative analysis	16 papers provided evidence that HCWs stereotype behaviours as normal when it may be a sign of pain. Assumptions around the experience of pain or behaviour may result in unnecessary respiratory compromise, admission to intensive care and prolonged hospital admission for the child with ID. There are

For peer review only

						specific aspects of hospitalisation that expose children with ID to harms that are preventable, avoidable and not experienced to the same extent by children without ID
Mitra et al[67]	2018	USA	Compare the risk of postpartum hospital admission and emergency department visits during the first postpartum year among women with and without ID	Total: 779,513 deliveries by Massachusetts women who gave birth in 2002–2012. Mothers with IDD: 1,104 Mothers without IDD: 778,409	Quantitative: Retrospective cohort study using chi-square, t-tests and Wilcoxon rank-sum tests	Women with IDD had higher prevalence rates for hospital admission and emergency department visits during all critical postpartum periods than those without IDD, with at least 2x higher rates for any hospitalisations within 1–42, 43–90, and 91–365 days after childbirth. Women with IDD also had a higher risk for repeated hospitalisations
Northway et al[85]	2017	UK	Map the content of existing hospital passports for people with ID to inform nursing practice and future research	Hospital passports of 60 people with ID in hospital	Qualitative: content analysis.	60 documents developed by provider organisations in the UK and Northern Ireland were reviewed and varied considerably in terms of length, title and content. Most frequent content included; Name, Level of communication (expression and understanding), Level of support required with nutrition, Mobility, Sleeping, Communication of pain and distress, Behaviour, Personal care, Allergies, Contact person. Patient and primary

For peer review only

						care information absent in some documents. Concerns it may give relatives or carers a false sense of security
Parish et al[68]	2015	USA	Explore and compare both the pregnancy outcomes of women with and without IDD, and health outcomes of children born to mothers with and without IDD	Total: 3,859,539 pregnant women<br>Women with IDD: 1,706<br>Women without IDD: 3,857,833	Quantitative: Retrospective cohort study using chi-square and logistic regressions	Women with IDD were significantly more likely to have a c-section, preterm birth or early or threatened labour and had increased risk of labour complications including preeclampsia or hypertensive complications, early labour, and other complications of pregnancy or birth than those without. They also had significantly longer days in the hospital for delivery for deliveries with and without complications
Poulton, Armstrong and Nanan[70]	2018	Australia	Investigate the impact of ADHD and the effect of stimulant medication on women's perinatal outcomes	Total: 5,056 women treated with stimulants for ADHD in 1982 - 2012 who gave birth in 1994 - 2012	Quantitative: Cohort study	Women treated for ADHD with stimulants at any time (before, before and during, or only after the index pregnancy) had lower rates of spontaneous labour, and higher rates of caesarean delivery, active new-born resuscitation, and neonatal admission. 4 h. stimulant treatment for ADHD before or before and during pregnancy was also associated with higher rates of preeclampsia, preterm birth, and low 1-min Apgar score
Purifoy et al[76]	2019	USA	Determine whether DS is	Total: 6,482 patients	Quantitative:	Patients with DS had longer

For peer review only

			associated with higher mortality, longer length of stay and greater incidence of gastrostomy and/or tracheostomy after complete repair of tetralogy of Fallot	aged 1 day - 19y old who had a diagnosis of DS and underwent complete surgical repair of tetralogy of Fallot in Jan 2004 – Dec 2016	Retrospective cohort study using chi-square	mean postoperative length of stay after complete repair and greater incidence of postoperative gastrostomy than those without. However, no significant difference in mortality prior to discharge or rates of postoperative tracheostomy
Read, Johnson and Tristan[86]	2012	UK	Identify areas of risk for patients with ID whilst in hospital to develop a rapid risk assessment tool for use in an acute hospital to assess immediate and potential risk, identify risk reduction actions and develop appropriate care bundles	Total: 54 rapid risk assessments with people with IDs admitted to the pilot wards	Improvement project: PDSA cycles	Implementation of the care bundles gave them structure and clear evidence-based guidance on which to deliver the best care for those with IDs. There was a reduction in bed days, lowering the risk of adverse events occurring, saving money in bed days and readmission penalties
Redley et al[55]	2019	UK	Understand the views of qualified medical practitioners regarding reasonable adjustments and the quality of the care and treatment provided to adult inpatients with ID	Total: 14 medical practitioners	Qualitative: Interview study using thematic analysis	Medical practitioners focused on two accounts: the patients' communication difficulties and vulnerability to behaviours that did not conform to a hospital's expectations, and their biomedical complexities. They reported making limited use of “reasonable adjustments" and turned to caregivers to facilitate communication and manage behaviours likely to upset hospital routines
Shah et al[37]	2009	Canada	Review outcomes and toxicity of chemotherapy for acute lymphoblastic leukaemia in	Total: 30 patients with DS and acute lymphoblastic leukaemia	Quantitative: Cohort study using Cox proportional hazards	The rate of infectious, respiratory, and gastrointestinal adverse

			children with DS	treated at a hospital in 1985 – 2004	and a matched generalized linear model	events was significantly higher in children with DS. Patients with DS also spent more days in hospital, particularly during the induction phase of treatment. However, the delays of subsequent treatment phases and differences between the need for intravenous opiate analgesia, parenteral nutrition and blood products were not significantly different
Sheehan et al[6]	2016	UK	Examine steps that hospitals are taking to deliver high-quality care to people with a LD, and examine any impact these have on care quality	Total: 176 patients case note audit from adults with LDs who received inpatient hospital care in acute general and mental health services in May 2013 - April 2014	Quantitative: Clinical case note audit study using multivariable logistic regression	The strongest performance was in ensuring that family or carers were involved in discharge planning (84% evidencing that this was enacted). Weight measurement or BMI was recorded in 58% notes. Compliance with all other audit criteria was <50%. Records of swallowing assessments, epilepsy risk assessment (for those with epilepsy) and that a health passport was used fared particularly badly, with evidence of these interventions in only 19%, 21% and 24% cases, respectively. For most quality indicators, there was a non-statistically significant trend for

For peer review only

						improved performance in services with a LD liaison nurse. The presence of an electronic flagging system showed less evidence of benefit
St Louis et al[78]	2014	USA	Descriptively analyse surgical outcomes from repair of complete atrioventricular septal defect in infants	Total: 2,399 patients who underwent surgery in 2008 – 2011.<br>With DS: 78.4%	Quantitative: Retrospective cohort study using Wilcoxon rank sum tests	Mortality and major complication rates are lower for patients with DS than for those without. Length of stay was similar. Although overall mortality is generally low for the entire cohort of patients undergoing repair of complete atrioventricular septal defect, patients at extremes of low weight and age have worse outcomes
Toth et al[77]	2013	Hungary	Compare postoperative morbidity and mortality of paediatric patients with and without DS who underwent heart surgery	Total: 1,6667 patients <18y old who underwent heart surgery and were admitted to the cardiac ICU in 2003 - Dec 2008<br>Neonates: 298<br>Infants: 570<br>Children: 799	Quantitative: Retrospective cohort study using $\chi 2$-test, Fisher's exact test and t-tests using a non-parsimonious multivariable logistic regression model	The occurrence of low output syndrome, pulmonary complication, renal failure and severe infection was higher in those with DS, and were more likely to undergo prolonged mechanical ventilation and have an increased length of hospital stay. However, after propensity matching, there was no significant variation between the group including differences in mortality
Tuffrey-Wijne et al[14]	2014	UK	To describe the challenges in preventing and monitoring patient safety issues for people with ID in NHS acute hospitals,	Survey response from 990 staff and 88 carers. Interviews with 68 hospital staff and 37	Mixed methods	Staff did not always readily identify patient safety issues or report them. Incident reports focused mostly around

For peer review only

			to describe the range of the patient safety issues faced by patients with intellectual disabilities in the study (from those that caused potential harm but no known harm, to those that caused actual harm); and to explore the underlying contributory factors to these safety issues	carers. Observation of in-patients with intellectual disabilities (n = 8); monitoring of incident reports (n = 272) and complaints involving people with intellectual disabilities		events causing immediate or potential physical harm, such as falls. Hospitals lacked effective systems for identifying patients with intellectual disabilities within their service, making monitoring safety incidents for this group difficult.<br><br>The safety issues described by the participants were mostly related to delays and omissions of care, in particular: inadequate provision of basic nursing care, misdiagnosis, delayed investigations and treatment, and non-treatment decisions and DNACPR orders
Tuffrey-Wijne et al[56]	2016	UK	Identify factors that affect carer involvement for people with ID in acute hospitals and develop guidance to promote effective carer involvement	Survey response from 990 staff and 88 carers. Interviews with 68 hospital staff and 37 carers	Mixed methods	Very high staff awareness of good practice. Carers were generally satisfied with how they were treated. However, a significant minority were dissatisfied on factors including expectations to provide basic nursing care, their expertise not being acted upon and discrepancies in perspective on the role of carers. A new model for clarifying carer involvement includes; the degree carers are 'workers' contributing to

For peer review only

						basic nursing care the degree carers are experts or non-experts. Making these two aspects of carer roles explicit may help staff to understand the particular contributions of carers, avoiding tensions
Vlassakova and Emmanouil[87]	2016	USA	Summarise experiences and recommendations for the perioperative management of children with autism	Studies focussed on children with autism	Literature review	Children with autism each display a unique behavioural profile. Collecting information about the patient in advance, establishing good rapport with the family, clear communication with all members of the perioperative team are key to success. Minimising perioperative stress, providing quiet environment, avoiding use of potential harmful medications assure smooth perioperative care and minimise adverse events
Wilkinson[63]	2018	UK	Produce a case study detailing the plan of care for an 18 year old male patient with a moderate level of LD who was scheduled for a tonsillectomy in hospital	Focussing on an 18 year old male patient with LD	Case study	Through collaboration and effective communication between practitioners, anaesthetists, surgeon and recovery care staff, the patient was admitted for his procedure with full knowledge of his individual needs and concerns, despite his limited communication skills. The NHS passport was a valuable document in the practitioners'

For peer review only

						toolbox, offering great merit in its holistic approach to patient care
Oulton et al[38]	2018	UK	Compare and identify factors that facilitate and prevent children and young people with and without LDs and long term conditions from receiving equal access to high quality hospital care and services	Interviews: 65 staff in senior clinical or managerial roles or those employed specifically to work with children and young people with LDs. Survey: 2,261 clinical and non-clinical staff with contact with children and young people and their families	Mixed methods	Two key themes; national variation and staff uncertainty. Lack of knowledge about policies systems at an organisational level to support care of children and young people with LDs. Considerable variation between hospitals ranging from those appearing to have few or no systems, policies or practices in place specifically for this group, with partial systems, policies or practices in place and those with a cohesive and comprehensive level of provision. There was a lack of standardised systems in place for communicating that an individual has a LD. Also a distinct lack of systems in place for recording that an individual is involved in a complaint or the subject of clinical incident has a LD
Pugely et al[79]	2014	USA	Analyse the incidence of, and risk factors for, short-term complications after paediatric deformity spinal surgery	Total: 2,005 Elective cases for deformity spinal surgery in patients <19y old	Quantitative: Retrospective cohort of a prospective cohort using univariate regression analysis	Risk factors for complications in children after deformity spinal surgery included developmental delay and CI. These variables were not associated with mortality
Vervloessem	2009	Netherlands	Compare complication rates	Total: 467 paediatric	Quantitative:	CI was a significant risk factor

For peer review only

et al[80]			with other centres and identify risk factors for major complications related to percutaneous endoscopic gastrostomy placement in children	patients undergoing percutaneous endoscopic gastrostomy placement at the hospital	Retrospective cohort study using univariate and multivariate logistic regression analyses	for complications

Notes. ADHD = Attention Deficit Hyperactivity Disorder, BMI = Body Mass Index, CCNs = Complex Communication Needs, CI = Cognitive Impairment, c-section = caesarean section, DNACPR = Do Not Attempt Cardiopulmonary Resuscitation, DS = Downs Syndrome, HCWs = Healthcare Workers, ICU = Intensive Care Unit, ID = Intellectual Disability, IDs = Intellectual Disabilities, IDD = Intellectual and Developmental Disabilities, IV = Intravenous Tubing, LD = Learning Disability, LDs = Learning Disabilities, PDSA = Plan Do Study Act, PPRs = Potentially Preventable Readmissions.

For peer review only

Online Supplementary Table 2. Included grey literature material with author, webpage, year, title, aims and key information (presented in ascending date order)

Author	Webpage	Year	Title	Aims	Key information
Mencap[39]	https://www.mencap.org.uk/sites/default/files/2016-08/treat_me_right.pdf	2004	Treat me right! Better healthcare for people with a learning disability	To highlight that despite many policy reports (starting in 1992) there have been few changes in health care delivery for people with learning disabilities	Use of case studies to illustrate need for change and recommendations for change:<br>- Training for health professionals that should involve people with a learning disability.<br>- All NHS organisations must fully comply with the DDA to provide equal access to healthcare<br>- Healthcare services must address the problem of health inequalities<br>- Hospitals must fulfil their legal duty of care and provide appropriate levels of support to patients who have a learning disability<br>- There must be an inquiry into the premature deaths of people with a learning disability
Mencap[40]	https://www.mencap.org.uk/sites/default/files/2016-06/DBIreport.pdf	2007	Death by indifference	To understand the causes of deaths for people with learning disabilities in hospital	The report presents six case studies. The report highlights that the underlying cause of death for many people with a learning disability who die in hospital is the widespread ignorance and indifference throughout our healthcare services towards people with a learning disability, and their families and carers
Allyson Kent[88]	https://www.nursingtimes.net/roles/learning-disability-nurses/improving-acute-care-of-people-with-learning-disabilities-05-02-2008/	2008	Improving acute care of people with learning disabilities	To report on the development of a patient passport system for use by people with learning disabilities entering acute care settings	The patient passport is a simple tool that articulates people's individual needs and seeks to bridge the communication gap in acute care. The patient passport has evolved as a result of listening to the needs of people with learning disabilities, parents and carers and acute staff
Elizabeth Piper[57]	https://hydra.hull.ac.uk/assets/h	2008	Investigating the experiences of people with learning	To investigate emergency	Interview findings include the relationship staff had with both service users and carers was

For peer review only

	ull:5754a/content		disabilities in Accident & Emergency from a carer perspective	healthcare - as delivered via Accident & Emergency - from the perspective of the carers of people with learning disabilities	considered to be fundamental to a high quality service. Themes identified included Interactions that are valuing, emotional responsiveness, support, compliance and responsibilities
Sir Jonathan Michael and the Independent Inquiry into Access to Healthcare for People with Learning Disabilities[41]	https://webarchive.nationalarchives.gov.uk/20130105064250/http://www.dh.gov.uk/en/Publicationsandstatistics/Publications/PublicationsPolicyAndGuidance/DH_099255	2008	Healthcare for all: report of the independent inquiry into access to healthcare for people with learning disabilities	To review research and evidence, and the views of witnesses and stakeholders to understand the safety of care for people with learning disabilities	The report highlighted that there are risks within the care system for people with learning disabilities and these risks are exacerbated by that lack of 'reasonable adjustments' to services. A large number of avoidable deaths were also found. The report makes recommendations for changes within the healthcare system and gives examples of how to implement these.
The Parliamentary and Health Service Ombudsman[42]	https://assets.publishing.service.gov.uk/government/uploads/system/uploads/attachment_data/file/250750/0203.pdf	2009	Six lives: the provision of public services to people with learning disabilities	To illustrate some significant failures in service across both health and social care for people with learning disabilities	Six investigation reports into deaths of people with learning disabilities following referral to the Ombudsman when complaints had not been satisfactorily answered by care providers;<br>The areas of concern included:<br>- Communication<br>- Partnership working and co-ordination<br>- Relationships with families and carers<br>- Failure to follow routine procedures<br>- Quality of management<br>- Advocacy<br>The case studies are powerful reminders of how things can go wrong, some with examples of unsafe care and adverse events in care delivered by NHS acute Trusts
Royal College of Nursing[89]	http://oxleas.nhs.uk/site-media/cms-downloads/RCN	2009	Dignity in health care for people with learning disabilities	To provide a resource to support nursing staff to improve	Examples of good practice that makes care safer for people with learning disabilities including staff training and accessibility of care

For peer review only

	_Dignity_in_healthcare.pdf			dignity in health care for people with learning disabilities	
Guidelines and Audit Implementation Network[90]	https://rqia.org.uk/RQIA/files/81/81662c46-b7bb-43a5-9496-a7f2d919c2a3.pdf	2010	Guidelines on caring for people with a learning disability in general hospital settings	To develop guidelines for care delivery to enhance safe and effective care throughout the journey within the general hospital setting for people with a learning disability	The guidelines, developed by a range of health professionals, support staff to provide safe and effective care for people with a learning disability. Guidance is given for each stage of hospital journey and include communication, attitudes, values and training for staff
The Hillingdon Hospitals NHS Foundation Trust[91]	https://www.thh.nhs.uk/about/safety/learning_dis.php<br><br>https://www.thh.nhs.uk/documents/_Patients/Guidelines_patients_learning_disabilities_Dec2011.pdf	Good Practice Guidelines - 2012	Responding to the needs of people with learning disabilities	To provide an equal service for people with learning disabilities	Examples of good practice from one Trust to improve safety and experience during hospital admission for patients with learning disabilities
The Confidential Inquiry into premature deaths of people with learning disabilities team[9]	https://www.hqip.org.uk/resource/confidential-enquiry-into-deaths-of-people-with-learning-disabilities-cipold-2013/#.XkP6jy7Fly4	2013	Confidential enquiry into deaths of people with Learning Disabilities	To investigate and compare the sequence of events leading to known deaths of 247 people, comprising people with learning disabilities and comparator cases from five Primary Care Trust areas	Most (96%) were of white UK ethnicity and 22% were under the age of 50 when they died. The median age of death for people with learning disabilities (65y for men; 63y for women) was significantly less than for the UK population (78y for men and 83y for women). Men with learning disabilities died, on average, 13 years sooner than men in the general population, and women with learning disabilities died 20 years sooner than women in the general population. The study revealed that the quality and effectiveness of health

				of South West England, to assess avoidable or premature deaths	and social care given to people with learning disabilities was deficient in a number of ways
Agency for Healthcare Research and Quality[92]	https://www.innovations.ahrq.gov/profiles/comprehensive-program-support-patients-and-staff-improves-hospital-experience-adult	2013	Service Delivery Innovation Profile Comprehensive program to support patients and staff improves hospital experience for adult patients with intellectual and developmental disabilities	To develop a comprehensive set of strategies for patients and staff designed to improve the hospital experience for adult patients with intellectual and developmental disabilities	The program provides pre-hospitalisation tours, a telephone "helpline" to request special accommodations, procedure-specific informational booklets, participation in a buddy program, and a collection of multisensory materials for relaxation and distraction. It has been reported to improve patient experience
Department of Health[43]	https://assets.publishing.service.gov.uk/government/uploads/system/uploads/attachment_data/file/212292/Six_lives_2nd_Progress_Report_on_Healthcare_for_People_with_Learning_Disabilities_-_full_report.pdf	2013	Six Lives: Progress Report on Healthcare for People with Learning Disabilities	To report progress in responding to the Ombudsmen's recommendations in 2010 following the 'Six Lives' report which investigated the deaths of six people with learning disabilities, first highlighted by Mencap	Progress on issues which were of particular concern to people with learning disabilities and family carers in 2010 are reported. Issues included capacity and consent; staff understanding (including communications, information and reasonable adjustments); and complaints and advocacy
Mencap[44]	https://www.mencap.org.uk/get-involved/campaign-mencap/hear-my-voice/hear-my-voice-healthcare	2014	Hear my voice: healthcare<br><br>Jayne and Jonathan's story about their brother Paul	NHS to take action to stop 1,200 preventable annual deaths of people with a learning disability in hospital by; everyone with a learning disability	Jayne and Jonathan shared the story of their brother Paul, who spent three weeks in intensive care before being prematurely transferred to a general ward where he experienced poor quality care, and later died. This involved; family members concerns of deterioration being disregarded, missing notes, prescribing drugs which worsened his condition and misinterpreting symptoms to be

For peer review only

	https://www.youtube.com/watch?v=JZA9cClHqWA&feature=emb_logo			getting a quality annual health check, a health plan, and offered a hospital passport. GPs, doctors and nurses to also have training on reasonable adjustments they need to make to give quality care to people with a learning disability	due to the learning disabilities. As a result the family initiated the campaign
Department of Health[93]	https://assets.publishing.service.gov.uk/government/uploads/system/uploads/attachment_data/file/309153/Strengthening_the_commitment_one_year_on_published.pdf	2014	Strengthening the Commitment: One year on: Progress report on the UK Modernising Learning Disabilities Nursing Review	To report the progress made in ensuring people with learning disabilities of all ages have access to expert learning disabilities nursing	Progress made in strengthening capacity, capability, quality and the profession are reported using recommendations and positive practice
1000 Lives Improvement, which is part of Public Health Wales[94]	http://www.1000livesplus.wales.nhs.uk/sitesplus/documents/1011/How%20to%20%2822%29%20Learning%20Disabilites%20Care%20Bundle%20web.pdf	2014	Improving general hospital care of patients who have a learning disability	To enable healthcare organisations and their teams to successfully implement a series of interventions to improve the safety and quality of care that patients with learning disabilities receive	An improvement guide that describes a care bundle of interventions and driver diagram. The guide also includes details of specific interventions in the appendices
Jim Blair[95]	http://www.intellectualdisability.i	2014	Everybody's life has worth – Getting it right in hospital for	To address the need for	Central reasonable adjustments for people with an intellectual disability are clear e.g. No

	nfo/how-to-guides/articles/everybodys-life-has-worth-getting-it-right-in-hospital-for-people-with-an-intellectual-disability-and-reducing-clinical-risks		people with an intellectual disability and reducing clinical risks	reasonable adjustments, and other issues, by using examples of a hospital passport, assessing the mental capacity of a person, and how to improve care provided and reduce clinical risks	fixed visiting times. Hospital passports vital and cover aspects other than health such as likes and dislikes, how communicating with the person, and how they express pain
Public Health England[96]	http://cdn.basw.co.uk/upload/basw_14709-8.pdf	2015	Working together 2: Easy steps to improve support for people with learning disabilities in hospital	An update of the Working together guide published in 2008 to help hospital staff, family members and paid support staff work jointly before, during and towards the end of any hospital admission (unplanned or planned) so an individual with learning disabilities could get good support and treatment	People with learning disabilities should get the help they need from health services, applying any reasonable adjustments required. Health professionals should listen more to the families and support staff of people with learning disabilities because they usually know most about the people they support and what help they need. Health staff should not assume that relatives or paid support staff of a person with learning disabilities will provide care while the person is in hospital; any such support must be discussed and agreed, taking account of their needs and supporting them appropriately. Case studies highlighted good positive practice in planning and delivering care
Patient Experience Network (not for profit organisation)<br><br>CHANGE	https://patientexperiencenetwork.org/wp-content/uploads/2019/10/Hidden-Voices-of-Maternity-	2015	Hidden Voices of Maternity: Parents with Learning Disabilities Speak Out	To capture the experience of parents with learning disabilities and offer recommendations for service	Provide training for health professionals to better support parents with learning disability, improve accessibility to services. Establish a visible lead in a provider organisation whose role is to support learning disabilities as opposed to mental health or other area

For peer review only

(national human rights organisation led by disabled people)[71]	Executive-Summary-FINAL-260815-2.pdf			improvements to support care to become more person- and family-centred	
Programme led by the University of Bristol and commissioned by the Healthcare Quality Improvement Partnership on behalf of NHS England[45]	https://www.bristol.ac.uk/media-library/sites/sps/leder/LeDeR annual report October 2016_FINAL v8.pdf	2016	The Learning Disabilities Mortality Review Annual Report	To evaluate the LeDeR programme	The 2015-2016 report found that there was a significantly higher mortality rate for people with learning disabilities compared to people without. Common underlying causes of mortality were circulatory and respiratory diseases and cancers. A high proportion of deaths were from causes classified as amenable to good medical care
The Westminster Commission on Autism[101]	https://westminsterautismcommission.files.wordpress.com/2016/03/ar1011_ncg-autism-report-july-2016.pdf	2016	A Spectrum of Obstacles<br><br>An Inquiry into Access to Healthcare for Autistic People	To highlight what good quality, person centred healthcare, tailored to the needs of those on the autistic spectrum, can achieve. The report is a call for ensuring equal access to quality healthcare for all on the autistic spectrum and to make this widespread and institutionalised.	The report follows a seven-month inquiry chaired by Barry Sheerman MP. Consultation with over 900 autistic people, families and professionals to investigate issues highlighted in the inquiry. The report revealed obstacles that autistic people encounter when accessing healthcare, and presents six recommendations around: Training; Inspection; Data; Annual Health Checks; Leadership; and Resources.
National Quality Board[97]	https://www.england.nhs.uk/wp-content/uploads/2017/03/nqb-national-	2017	National Guidance on Learning from Deaths	To provide a framework for NHS Trusts and NHS Foundation Trusts on identifying,	Guidance to providers:<br>- All deaths of people with learning disabilities aged four years and older are subject to review using LeDeR methodology<br>- The LeDeR programme is currently being

For peer review only

	guidance-learning-from-deaths.pdf			reporting, investigating and learning from deaths in care	rolled out across England. Full coverage is anticipated in all Regions by the end of 2017. If there is a death of a person with learning disabilities in an acute setting in an area that is not yet covered by the LeDeR programme, Trusts are recommended to use the SJR process or a methodology of equivalent quality that meets the requirements for the data that must be collected as an interim measure
Programme led by the University of Bristol and commissioned by the Healthcare Quality Improvement Partnership on behalf of NHS England[46]	https://www.hqip.org.uk/wp-content/uploads/2018/05/LeDeR-annual-report-2016-2017-Final-6.pdf	2018	The Learning Disabilities Mortality Review Annual Report	To evaluate the LeDeR programme	The 2017 annual report suggested that 1,311 deaths were notified to the programme. Most people male (57%), single (96%) and of white ethnic background (93%). Just over a quarter had mild learning disabilities (27%), 33% had moderate learning disabilities, 29% severe learning disabilities and 11% profound or multiple learning disabilities. Approximately one in ten usually lived alone and had been in an out-of-area placement (9%)
NHS England[98]	https://www.england.nhs.uk/wp-content/uploads/2018/10/ask-listen-do-for-organisations-and-practitioners-v1.pdf	2018	Ask Listen Do Resource for Organisations and Practitioners	Tips for health, social care and education organisations and practitioners<br><br>Making feedback, concerns and complaints easier for people with a learning disability, autistic people, families and carers	This Ask, Listen, Do resource is for social care, health and education organisations, large or small, and their practitioners who support people with a learning disability, and autistic people. Four key themes within the resource; partnership, communication, processes and leadership
Mencap and The National Autistic Society: Treat me well campaign[99]	https://www.mencap.org.uk/get-involved/campaign-mencap/treat-	2018	Oliver McGowan mandatory training in learning disability and autism	Train health and social care staff to provide better health and social care outcomes for	A wider roll-out of the training is underway, with plans to evaluate. Mencap has already delivered the training to around 1,800 healthcare staff. 98% of participants said they wanted to change the way they deliver

For peer review only

	me-well/announcement-learning-disability-training-health-and			people with a learning disability and autism, focused on raising awareness and understanding. The training is co-designed and co-delivered by people with a learning disability, autism, family carers and experts in the subject matter	healthcare for people with a learning disability after taking part
Paula McGowan[48]	https://www.england.nhs.uk/blog/ask-listen-do-olivers-story/	2018	Blog<br><br>Ask Listen Do: Oliver's Story	Demonstrating the importance of the principles of Ask, Listen Do for healthcare providers of those with a learning disability to better understand and manage situations safely	Oliver had mild cerebral palsy, focal epilepsy and mild autism, and was admitted to hospital due to seizure activity. Oliver's health deteriorated and he passed away a few weeks later due to neuroleptic malignant syndrome, a rare but serious side effect of antipsychotic medications given to control his agitation in hospital, caused by his epilepsy and autism
NHS Improvement[100]	https://www.england.nhs.uk/wp-content/uploads/2020/08/v1.17_Improvement_Standards_added_note.pdf	2018	The learning disability improvement standards for NHS Trusts	Develop new standards to help NHS Trusts measure the quality of care they provide to people with learning disabilities, autism or both	The report comprises four key standards; respecting and protecting rights, inclusion and engagement, workforce and specialist learning disability services
Programme led by the University of Bristol and commissioned	https://www.hqip.org.uk/resource/the-learning-disabilities-	2019	The Learning Disabilities Mortality Review Annual Report	To evaluate LeDeR programme	The 2018 annual report suggested that 4,302 deaths were notified to the programme, approximately 86% of the estimated number of deaths of people with learning disabilities in

by the Healthcare Quality Improvement Partnership on behalf of NHS England[47]	mortality-review-annual-report-2018/ - .XkP6Wi7FIy4				England each year. The proportion of people with learning disabilities dying in hospital is higher (62%) than in the general population (46%). Almost a half (48%) of deaths received care that the reviewer felt met or exceeded good practice. The proportion of deaths from people from Black, Asian and Minority Ethnic groups was lower (10%), than that from the population in England as a whole (14%). However, children and young people from BAME groups were overrepresented in deaths of people with learning disabilities
NHS England[64]	https://www.longtermplan.nhs.uk/publication/nhs-long-term-plan/	2019	The NHS long term plan	To set out a long term plan to make the NHS fit for the future	Providing the right care and support for children with a learning disability is part of long term plan. The plan specifies that the whole NHS will improve its understanding of the needs of people with learning disabilities and autism, and work together to improve their health and wellbeing. NHS staff will receive information and training on supporting people with a learning disability and/ or autism. National learning disability improvement standards will be implemented and will apply to all services funded by the NHS
NHS England and NHS improvement[49]	https://improvement.nhs.uk/documents/5472/190708_Patient_Safety_Strategy_for_website_v4.pdf	2019	The NHS patient safety strategy	To develop a patient safety culture and a patient safety system	Must ensure people with a learning disability are more visible; that they are listened to; and that reasonable adjustments are made to ensure they have better access to healthcare. Mandatory training on learning disability and autism to give health and care staff the knowledge and skills to accomplish this. Need understanding of safety issues: reduce harm from the effects of inappropriate psychotropic medicine use, care and treatment reviews. By 2023/24 all NHS-commissioned care will meet the learning disability improvement standards
Department of Health and	https://www.gov.uk/government/	2019	Consultation outcome. Learning disability and	To gain a better understanding of	Mandatory learning disability and autism training was one of the commitments made in

Social Care[65]	consultations/learning-disability-and-autism-training-for-health-and-care-staff		autism training for health and care staff	how to ensure that patients and service users receive safe, effective and dignified care and that those who provide care have the knowledge, skills and behaviours to support people with learning disabilities and autistic people	in the Government's response to the second annual report of the LeDeR Programme. Training should focus on understanding learning disability and autism, the legislative context and making reasonable adjustments
Healthcare Safety Investigation Branch[50]	https://www.hsib.org.uk/documents/139/hsib_interim_bulletin_undiagnosed_cardiomyopathy_autism.pdf	2019	Undiagnosed cardoimyopathy of a young person with Autism	To investigate the death of an autistic patient with an undiagnosed heart problem	Identified safety issues included: The patient was not seen by a learning disability specialist and their altered physiological markers were attributed to emotional distress. Lack of national guidelines on aesthetic assessment and threshold for patients with learning disabilities and autism
Care Quality Commission[51]	https://www.cqc.org.uk/help-advice/your-stories/declare-your-care-people-learning-disabilities	2020	Declare Your Care: People with learning disabilities	A year-long, campaign focusing on four key population groups which have lower awareness of CQC	Survey findings revealed that people with a learning disability are more likely to regret not complaining about poor care than those without. The main reasons they or their carers want to raise a concern were: lack of information about a health condition and treatment options are not well explained. Stories presented

NIHR dissemination centre[52]	https://content.nihr.ac.uk/nihrdc/themedreview-04326-BCAHFA/Better-Health_Care-For-FINALWEB.pdf	2020	NIHR themed review: health and care services for people with learning disabilities	To review NIHR research on health and care services for people with learning disabilities	There is consistent evidence from confidential enquiries into unexpected deaths that general hospitals vary greatly in how well they make adjustments for patients who have learning disabilities. Research indicates problems in how hospitals implement these recommendations including confidence of general hospital staff in addressing the needs of patients. Learning disability nurses were found to make valued contributions to care. Little is known about what difference patient passports make to how well staff are able to adjust the care they provide
Programme led by the University of Bristol and commissioned by the Healthcare Quality Improvement Partnership on behalf of NHS England[11]	https://www.hqip.org.uk/wp-content/uploads/2020/07/LeDeR_2019_annual_report_FINAL.pdf	2020[a]	The Learning Disabilities Mortality Review Annual Report	To evaluate the LeDeR programme	The 2019 annual report suggested that 7,145 deaths were notified to the programme, 6,629 were adults and 516 were children (4-17y). The review process had been completed for 45% of these deaths. 58% were males; 90% were white British; 30% had mild learning disabilities, 33% had moderate learning disabilities, 27% severe learning disabilities and 10% profound and multiple learning disabilities. People with profound and multiple learning disabilities, and people from Black, Asian and Minority Ethnic groups died disproportionately at younger ages

Notes. CQC = Care Quality Commission, DDA = Disability Discrimination Act, GP = General Practitioner, LeDeR = Learning Disabilities Mortality Review, MCA = Mental Capacity Act, NHS = National Health Service, NICE = National Institute for Health and Care Excellence, NIHR = National Institute for Health Research, SJR = Structured Judgement Review.

[a]This report was published in July 2020 after our initial grey literature search.

For peer review only

Preferred Reporting Items for Systematic reviews and Meta-Analyses extension for Scoping Reviews (PRISMA-ScR) Checklist

SECTION	ITEM	PRISMA-ScR CHECKLIST ITEM	REPORTED ON PAGE #
**TITLE**			
Title	1	Identify the report as a scoping review.	1
**ABSTRACT**			
Structured summary	2	Provide a structured summary that includes (as applicable): background, objectives, eligibility criteria, sources of evidence, charting methods, results, and conclusions that relate to the review questions and objectives.	2
**INTRODUCTION**			
Rationale	3	Describe the rationale for the review in the context of what is already known. Explain why the review questions/objectives lend themselves to a scoping review approach.	4
Objectives	4	Provide an explicit statement of the questions and objectives being addressed with reference to their key elements (e.g., population or participants, concepts, and context) or other relevant key elements used to conceptualize the review questions and/or objectives.	4
**METHODS**			
Protocol and registration	5	Indicate whether a review protocol exists; state if and where it can be accessed (e.g., a Web address); and if available, provide registration information, including the registration number.	n/a
Eligibility criteria	6	Specify characteristics of the sources of evidence used as eligibility criteria (e.g., years considered, language, and publication status), and provide a rationale.	4-5
Information sources*	7	Describe all information sources in the search (e.g., databases with dates of coverage and contact with authors to identify additional sources), as well as the date the most recent search was executed.	5-6
Search	8	Present the full electronic search strategy for at least 1 database, including any limits used, such that it could be repeated.	Appendix 1
Selection of sources of evidence†	9	State the process for selecting sources of evidence (i.e., screening and eligibility) included in the scoping review.	6
Data charting process‡	10	Describe the methods of charting data from the included sources of evidence (e.g., calibrated forms or forms that have been tested by the team before their use, and whether data charting was done independently or in duplicate) and any processes for obtaining and confirming data from investigators.	6
Data items	11	List and define all variables for which data were sought and any assumptions and simplifications made.	n/a
Critical appraisal of individual sources of evidence§	12	If done, provide a rationale for conducting a critical appraisal of included sources of evidence; describe the methods used and how this information was used in any data synthesis (if appropriate).	n/a
Synthesis of results	13	Describe the methods of handling and summarizing the data that were charted.	6-7

For peer review only - http://bmjopen.bmj.com/site/about/guidelines.xhtml

SECTION	ITEM	PRISMA-ScR CHECKLIST ITEM	REPORTED ON PAGE #
**RESULTS**			
Selection of sources of evidence	14	Give numbers of sources of evidence screened, assessed for eligibility, and included in the review, with reasons for exclusions at each stage, ideally using a flow diagram.	8
Characteristics of sources of evidence	15	For each source of evidence, present characteristics for which data were charted and provide the citations.	9
Critical appraisal within sources of evidence	16	If done, present data on critical appraisal of included sources of evidence (see item 12).	n/a
Results of individual sources of evidence	17	For each included source of evidence, present the relevant data that were charted that relate to the review questions and objectives.	Tables 1 and 2
Synthesis of results	18	Summarize and/or present the charting results as they relate to the review questions and objectives.	9-13
**DISCUSSION**			
Summary of evidence	19	Summarize the main results (including an overview of concepts, themes, and types of evidence available), link to the review questions and objectives, and consider the relevance to key groups.	14-15
Limitations	20	Discuss the limitations of the scoping review process.	15
Conclusions	21	Provide a general interpretation of the results with respect to the review questions and objectives, as well as potential implications and/or next steps.	15-16
**FUNDING**			
Funding	22	Describe sources of funding for the included sources of evidence, as well as sources of funding for the scoping review. Describe the role of the funders of the scoping review.	26

JBI = Joanna Briggs Institute; PRISMA-ScR = Preferred Reporting Items for Systematic reviews and Meta-Analyses extension for Scoping Reviews.

* Where *sources of evidence* (see second footnote) are compiled from, such as bibliographic databases, social media platforms, and Web sites.

† A more inclusive/heterogeneous term used to account for the different types of evidence or data sources (e.g., quantitative and/or qualitative research, expert opinion, and policy documents) that may be eligible in a scoping review as opposed to only studies. This is not to be confused with *information sources* (see first footnote).

‡ The frameworks by Arksey and O'Malley (6) and Levac and colleagues (7) and the JBI guidance (4, 5) refer to the process of data extraction in a scoping review as data charting.

§ The process of systematically examining research evidence to assess its validity, results, and relevance before using it to inform a decision. This term is used for items 12 and 19 instead of "risk of bias" (which is more applicable to systematic reviews of interventions) to include and acknowledge the various sources of evidence that may be used in a scoping review (e.g., quantitative and/or qualitative research, expert opinion, and policy document).

From: Tricco AC, Lillie E, Zarin W, O'Brien KK, Colquhoun H, Levac D, et al. PRISMA Extension for Scoping Reviews (PRISMAScR): Checklist and Explanation. Ann Intern Med. 2018;169:467–473. doi: 10.7326/M18-0850.

For peer review only - http://bmjopen.bmj.com/site/about/guidelines.xhtml

BMJ Open

Exploring patient safety outcomes for people with learning disabilities in acute hospital settings: A scoping review

Journal:	*BMJ Open*
Manuscript ID	bmjopen-2020-047102.R1
Article Type:	Original research
Date Submitted by the Author:	15-Apr-2021
Complete List of Authors:	Louch, Gemma; Bradford Teaching Hospitals NHS Foundation Trust, Bradford Institute for Health Research; NIHR Yorkshire and Humber Patient Safety Translational Research Centre<br>Albutt, Abigail; Bradford Teaching Hospitals NHS Foundation Trust, Bradford Institute for Health Research; NIHR Yorkshire and Humber Patient Safety Translational Research Centre<br>Joanna Harlow-Trigg , Joanna; University of Leeds, School of Psychology<br>Moore, Sally; Bradford Teaching Hospitals NHS Foundation Trust, Bradford Institute for Health Research<br>Smyth, Kate ; NIHR Yorkshire and Humber Patient Safety Translational Research Centre; Lancashire Teaching Hospitals NHS Foundation Trust<br>Ramsey, Lauren; Bradford Teaching Hospitals NHS Foundation Trust, Bradford Institute for Health Research ; NIHR Yorkshire and Humber Patient Safety Translational Research Centre<br>O'Hara, Jane; University of Leeds, School of Healthcare; NIHR Yorkshire and Humber Patient Safety Translational Research Centre
**Primary Subject Heading**:	Health services research
Secondary Subject Heading:	Health services research
Keywords:	Quality in health care < HEALTH SERVICES ADMINISTRATION & MANAGEMENT, Organisation of health services < HEALTH SERVICES ADMINISTRATION & MANAGEMENT, HEALTH SERVICES ADMINISTRATION & MANAGEMENT

SCHOLARONE™
Manuscripts

I, the Submitting Author has the right to grant and does grant on behalf of all authors of the Work (as defined in the below author licence), an exclusive licence and/or a non-exclusive licence for contributions from authors who are: i) UK Crown employees; ii) where BMJ has agreed a CC-BY licence shall apply, and/or iii) in accordance with the terms applicable for US Federal Government officers or employees acting as part of their official duties; on a worldwide, perpetual, irrevocable, royalty-free basis to BMJ Publishing Group Ltd ("BMJ") its licensees and where the relevant Journal is co-owned by BMJ to the co-owners of the Journal, to publish the Work in this journal and any other BMJ products and to exploit all rights, as set out in our licence.

The Submitting Author accepts and understands that any supply made under these terms is made by BMJ to the Submitting Author unless you are acting as an employee on behalf of your employer or a postgraduate student of an affiliated institution which is paying any applicable article publishing charge ("APC") for Open Access articles. Where the Submitting Author wishes to make the Work available on an Open Access basis (and intends to pay the relevant APC), the terms of reuse of such Open Access shall be governed by a Creative Commons licence – details of these licences and which Creative Commons licence will apply to this Work are set out in our licence referred to above.

Other than as permitted in any relevant BMJ Author's Self Archiving Policies, I confirm this Work has not been accepted for publication elsewhere, is not being considered for publication elsewhere and does not duplicate material already published. I confirm all authors consent to publication of this Work and authorise the granting of this licence.

For peer review only

Exploring patient safety outcomes for people with learning disabilities in acute hospital settings: A scoping review

Gemma Louch[1 2], Abigail Albutt[1 2], Joanna Harlow-Trigg[1 4], Sally Moore[1], Kate Smyth[2 3], Lauren Ramsey[1 2] and Jane K. O'Hara[1 2 5]

1 Yorkshire Quality and Safety Research Group, Bradford Institute for Health Research, Bradford, UK

2 NIHR Yorkshire and Humber Patient Safety Translational Research Centre, UK

3 Lancashire Teaching Hospitals NHS Foundation Trust

4 School of Psychology, University of Leeds, Leeds, UK

5 School of Healthcare, University of Leeds, Leeds, UK

Corresponding author:
Dr Gemma Louch
Gemma.Louch@bthft.nhs.uk

Twitter @Gemma_Louch

Contributors:
GL conceived the idea for the scoping review, led all stages of the scoping review and drafted the manuscript. AA contributed to all stages of the scoping review. KS reviewed the protocol, contributed to the search strategy and worked with the research team to develop materials for the wider Patient and Public Involvement and Engagement (PPIE) approach. JHT, SM and LR contributed to the literature searching, screening and data extraction. JOH contributed to the consistency checks and wrote the first draft of the discussion. All authors provided comments and approved the final version.

Funding:
The research was funded by the National Institute for Health Research Yorkshire and Humber Patient Safety Translational Research Centre (NIHR YH PSTRC). The views expressed in this article are those of the author(s) and not necessarily those of the NHS, the NIHR or the Department of Health and Social Care.

Competing interests: None declared.

Patient consent for publication: Not required.

For peer review only - http://bmjopen.bmj.com/site/about/guidelines.xhtml

Abstract

Objectives

To produce a narrative synthesis of published academic and grey literature focussing on patient safety outcomes for people with learning disabilities in an acute hospital setting.

Design

Scoping review with narrative synthesis.

Methods

The review followed the six stages of the Arksey and O'Malley framework. We searched four research databases from January 2000 to March 2021, in addition to hand searching and backwards searching using terms relating to our eligibility criteria – patient safety and adverse events, learning disability, and hospital setting. Following stakeholder input, we searched grey literature databases and specific websites of known organisations until March 2020. Potentially relevant articles and grey literature materials were screened against the eligibility criteria. Findings were extracted and collated in data charting forms.

Results

45 academic articles and 33 grey literature materials were included, and we organised the findings around six concepts: 1) Adverse events, patient safety and quality of care; 2) Maternal and infant outcomes; 3) Post-operative outcomes; 4) Role of family and carers; 5) Understanding needs in hospital; and 6) Supporting initiatives, recommendations and good practice examples. The findings suggest inequalities and inequities for a range of specific patient safety outcomes including adverse events, quality of care, maternal and infant outcomes and post-operative outcomes, in addition to potential protective factors, such as the roles of family and carers and the extent to which health professionals are able to understand the needs of people with learning disabilities.

Conclusion

People with learning disabilities appear to experience poorer patient safety outcomes in hospital. The involvement of family and carers, and understanding and effectively meeting the needs of people with learning disabilities may play a protective role. Promising interventions and examples of good practice exist, however many of these have not been implemented consistently and warrant further robust evaluation.

Keywords

Learning disability, patient safety, hospital, adverse events, intellectual disability

Strengths and limitations of this study

- A key strength is the synthesis of both academic and grey literature materials
- A further strength is our approach to patient and public involvement and engagement throughout the review process
- We did not conduct formal quality assessments and are therefore unable to make reflections and comparisons of article quality

For peer review only - http://bmjopen.bmj.com/site/about/guidelines.xhtml

Introduction

Inequalities in health and inequities in access to healthcare and technologies are a persistent and significant problem.[1 2 3] It is clear from previous research that certain demographic factors are associated with increased likelihood of poorer health, and variation in the use of and access to healthcare services.[4 5]

One population that may experience greater vulnerabilities in terms of health and healthcare inequalities are people with learning disabilities. These vulnerabilities might arise as a result of barriers to accessing services and challenges associated with service organisation and delivery.[6] Learning disabilities are defined as 'the presence of a significantly reduced ability to understand new or complex information, to learn new skills (impaired intelligence), with a reduced ability to cope independently (impaired social functioning) which started before adulthood, with a lasting effect on development'[a]. In this review we have also drawn from the definition presented in the White Paper *Valuing People*,[7] which states that learning disability includes the presence of:

- a significantly reduced ability to understand new or complex information, to learn new skills (impaired intelligence), with;
- a reduced ability to cope independently (impaired social functioning);
- which started before adulthood, with a lasting effect on development.

This broad definition includes adults with autism who also have learning disabilities, but not those with a higher-level autistic spectrum disorder, such as some people with Asperger's Syndrome. Learning disability is the term most commonly used in the UK, although it is recognised as being synonymous with intellectual disability.[8]

In 2013, the final report of a Confidential Inquiry into Premature Deaths of People with Learning Disabilities (CIPOLD) in England was published.[9] The report found that people with learning disabilities have higher rates of avoidable death compared to the general population, and that avoidable deaths arising from causes relating to poorer quality healthcare were more common in this population. On average, the life expectancy of people with learning disabilities is shorter than the general population.[10] The 2019 Learning Disabilities Mortality Review (LeDeR) report highlighted that people with learning disabilities died from an avoidable medical cause of death twice as frequently as people in the general population, and that the greatest difference between people with learning disabilities and the general population was in relation to medical causes of death which are treatable with access to timely and effective healthcare.[11]

In the UK the need for accessible healthcare environments for people with autism is recognised,[12] and in 2019, the Government announced plans to pilot and then roll out learning disability and autism mandatory training for health and care staff in England[b]. Furthermore, national projects such as Stopping Over-Medication of People with a Learning Disability, Autism or Both (STOMP)[c] have addressed issues around medicines practices.

---

[a] https://www.datadictionary.nhs.uk/data_dictionary/nhs_business_definitions/l/learning_disability_de.asp?shownav=1
[b] https://www.gov.uk/government/consultations/learning-disability-and-autism-training-for-health-and-care-staff
[c] https://www.england.nhs.uk/learning-disabilities/improving-health/stomp/

For peer review only - http://bmjopen.bmj.com/site/about/guidelines.xhtml

Although there is increasing interest in this important issue from academics, healthcare staff, managers and policy makers, much of this has focussed on health inequalities and healthcare access more generally. What has been lacking to date is a critical examination of this issue as a patient safety phenomenon. This is important, as it opens up new avenues for conceptualising this problem, along with different framings for potential improvement and service development.

There is clear evidence that people with learning disabilities may be more at risk in terms of patient safety in hospital as well as known challenges around recognising and reporting patient safety incidents in this population.[13 14 15] Therefore, the need to bring together what is known about the safety of people with learning disabilities receiving healthcare, is clear.

In this review, we aimed to produce a narrative synthesis of published academic and grey literature focussing on people with learning disabilities in an acute hospital setting. We limited this review to the hospital setting because we were particularly interested in the care people with learning disabilities receive in a setting that may be predominantly related to physical health. We aimed to generate evidence that may facilitate the development of more tailored patient safety interventions for people with learning disabilities in an acute hospital setting. Our specific objectives were to:

1) Understand patient safety and adverse events in this population;
2) Explore protective factors and potential explanatory mechanisms;
3) Identify patient safety interventions, improvement initiatives, recommendations and examples of good practice.

Methods

A scoping review was considered the most suitable approach to produce a comprehensive, yet broad overview of the topic area[16 17] We used Arksey and O'Malley's[18] six stage framework and subsequent amendments to guide the review.[16 19] The stages include: (1) identifying the research question(s); (2) identifying relevant research studies; (3) selecting relevant research studies; (4) charting the data; (5) collating, summarising and reporting the study findings; and (6) consulting with key stakeholders throughout the process. The review has been drafted in line with the PRISMA Extension for Scoping Reviews (PRISMA-ScR).[20] We developed a broad search strategy, informed by the PRISMA Extension for Systematic Reviews with a Focus on Health Equity (PRISMA-Equity, 2012).[21]

Patient and public involvement and engagement (PPIE)

Our review team includes a lay representative (co-author) who provided input into the protocol, reviewed the search strategy and helped develop materials for the wider PPIE approach. We invited stakeholders to contribute search terms and assist in identifying grey literature. Stakeholders included representatives from the Yorkshire Quality and Safety Research (YQSR) Group patient panel, representatives from the NIHR Yorkshire and Humber Patient Safety Translational Research Centre (NIHR YH PSTRC) Citizen Participation Group and healthcare staff.

Eligibility criteria

The 'Population-Concept-Context' (PCC) approach was used to specify study characteristics.[16] Inclusion and exclusion criteria were developed and iteratively refined as

For peer review only - http://bmjopen.bmj.com/site/about/guidelines.xhtml

the review progressed. Studies reporting on patient safety, adverse events, protective factors, potential explanatory mechanisms, intervention and improvement initiatives, recommendations and good practice examples related to these topic areas were eligible. There was no restriction of study design, quantitative and qualitative methodologies were eligible for inclusion, and we limited the search to English language only.

Inclusion criteria

- Articles that report on people with learning disabilities as the core focus (population). Articles may use terms synonymous with learning disability such as intellectual disability or refer to a condition related to learning disability, for example autism[d], attention deficit hyperactivity disorder[e] or Down Syndrome.
- Articles that investigate adverse events, patient safety, protective factors, potential explanatory mechanisms, patient safety interventions and improvement initiatives, recommendations and good practice examples (concept).
- Articles relating to patients receiving care in an acute hospital setting (context). No restriction on age.
- Articles relating to any country (context).
- Study type: No restriction - qualitative, quantitative, mixed methods, case studies, primary research, retrospective review, systematic or scoping reviews/ integrative reviews / meta-synthesis.
- Language: only articles published in the English Language due to lack of resources for an interpreter.

Exclusion criteria

- Articles relating to primary care settings and inpatient mental health settings.
- Articles focussing on patient experience/ satisfaction.
- Articles focussing on a specific drug treatment or procedure without a non-learning disability comparison group.

Information sources and search strategy

Academic literature search

The search terms built on terms used in prior reviews framed around the eligibility criteria.[15 22 23 24 25] An initial limited search of MEDLINE was conducted (Appendix 1). The search strategy was peer reviewed by a Knowledge and Information Librarian reviewer using the Peer Review of Electronic Search Strategies (PRESS),[26] and reviewed by academic researchers (patient safety), lay representatives and learning disability healthcare professionals. Following the initial search, all four included databases were searched: MEDLINE, CINAHL, PsycINFO and Web of Science from 2000 until March 12th 2021. The time period searched from was 2000 in line with the seminal publication of 'To Err is Human: Building a Safer Health System' as this publication arguably launched the modern patient safety movement.[27] The search was organised in three blocks: Block 1 – terms relating to learning disability (combined with OR); Block 2 – terms relating to adverse events and patient safety (combined with OR); Block 3 – terms relating to acute hospital setting (combined with OR). Blocks 1-3 were combined with the AND function. The reference lists of included articles were assessed, and we hand searched targeted journals including: the

[d]Autism and learning disabilities are often co-associated.[28 29]
[e]High comorbidity for learning disabilities and attention deficit hyperactivity disorder.[30]

For peer review only - http://bmjopen.bmj.com/site/about/guidelines.xhtml

British Journal of Learning Disabilities, Journal of Learning Disabilities, Journal of Intellectual Disability Research, BMJ Quality and Safety, Journal of Patient Safety, Health Expectations, BMC Health Services Research, BMJ Open.

Grey literature search

The grey literature search included suggestions made via stakeholder input, such as terms to search, known publicly available materials and specific organisations to search online (Appendix 2). We searched using the same combinations of terms relating to our eligibility criteria (e.g. 'patient safety and learning disability', 'learning disability and hospital', 'learning disability and adverse events'). All the online materials returned were initially screened according to title/summary information. In addition, the first 100 pages of Google, Google Scholar and all materials returned from OpenGrey and Royal College of Nursing Database were screened. The latest date for grey literature searches was 10th March 2020.

Study selection

Identified articles were collated in reference software (EndNote) and duplicates removed. Study selection involved two levels of screening: 1) title and abstract 2) full-text. Three reviewers (GL, AA, JHT) screened at title and abstract level according to the eligibility criteria, and 10% were independently checked to assess agreement. Articles that appeared to be eligible were screened at full-text level. When a full-text was unavailable, authors were contacted directly. We were unable to obtain two full-texts. Two independent reviewers assessed the full-text articles (GL, AA) and at this stage the reasons for exclusion were recorded. There were no discrepancies between reviewers regarding the eligibility of articles. Two authors carried out the grey literature search (GL, AA), and one author independently screened the potential grey literature for inclusion (SM), and 10% were independently checked to assess agreement.

Charting the data

Standardised data collection forms were developed and information from academic articles and grey literature material were collated into separate data collection forms, which were piloted prior to full data extraction.[19] For academic articles, key data were extracted including: publication year, publication type, country, study design, population, and summary information relating to adverse events, patient safety focus, protective factors, potential explanatory mechanisms, patient safety intervention or improvement initiatives, recommendations and good practice examples. Following piloting, two reviewers (AA, JHT) independently extracted the data from all included articles, and one reviewer checked 10% of the data extracted for consistency (JOH).

Study quality was not assessed as the aim of the review was to synthesise the emerging evidence within the area rather than assess quality of individual articles. The grey literature data collection form was amended from the research article data collection form. Three reviewers (SM, AA, LR) independently extracted the data from all included publications using the adapted data collection form, and one reviewer checked 10% of the data extracted for consistency (JOH).

Data synthesis

Data were collated in two spreadsheets, one for academic articles and one for grey literature. A narrative synthesis followed to develop a narrative description of the findings

For peer review only - http://bmjopen.bmj.com/site/about/guidelines.xhtml

and to highlight concepts that key findings could be organised around.[31 32] Authors (GL, AA, SM, LR, JOH) held meetings to discuss the key findings and generate concepts.

Results

Title and abstract screening identified 140 articles eligible for full-text screening. Where studies appeared in review articles that met the eligibility criteria, these were not analysed separately and excluded (n = 7). Thirty-four articles were eligible for inclusion in the review. A further 11 articles were included via backward and hand searching. In total, 45 articles were included (see figure 1). The grey literature search identified 92 potentially eligible materials, and 33 were included.

Summary characteristics

Characteristics of included articles and grey literature materials are displayed in Appendix 3 and 4.

Of the academic articles, nineteen related to paediatric patients, five to pregnant women/infant outcomes, four to adult patients, two to healthcare staff, one to healthcare staff and carers, one to parents or guardians, and thirteen articles related to hospital patients/setting more generally or did not specify the participants in more detail. All studies and reviews were conducted in high-income countries. Eighteen articles were from the USA, thirteen were from the UK, eight were from Australia, two were from Canada, two were from Taiwan, and one each was from Hungary and The Netherlands. Twenty-one articles were retrospective and/or cohort studies, six were a type of literature review, four were discussion/opinion pieces, three articles used mixed methods, three improvement projects, two were qualitative, two were featured/special interest articles, one commentary, one case study, one short report, and one secondary analysis. Fourteen articles referred specifically to intellectual disability, ten to Down Syndrome, eight to learning disability, five to autism, three to intellectual and developmental disability, two to communication disability, one to developmental delay, one to cognitive impairment, and one to attention deficit hyperactivity disorder.[f]

Key concepts

Our data synthesis generated six concepts: 1) Adverse events, patient safety and quality of care; 2) Maternal and infant outcomes; 3) Post-operative outcomes; 4) Role of family and carers; 5) Understanding needs in hospital; and 6) Supporting initiatives, recommendations and good practice. We present these concepts below and specify how they map onto the three review objectives.

Objective 1 - Understand patient safety and adverse events in this population.

Adverse events, patient safety and quality of care

Six articles concentrated on either specific types of adverse events, quality of care, or had a patient safety focus (see table 1). A systematic review of the experience of iatrogenic harm during hospitalisation for children with intellectual disability found that there are specific aspects of hospitalisation that expose children with intellectual disability to harms that are preventable, avoidable and not experienced to the same extent by children without

[f]Throughout the results section we use the same terms as those used in the original articles and grey literature materials.

For peer review only - http://bmjopen.bmj.com/site/about/guidelines.xhtml

intellectual disability.[15] Also focussed on children, a further study indicated that children with pre-existing cognitive impairment received lower doses of analgesia and sedation medication, although the authors acknowledged it was not clear whether this was due to lower requirements or inadequate assessment.[33]

An integrative review investigated the care and safety of adults with communication disabilities in hospital and included a significant amount of studies specifically focussed on intellectual disability.[34] The review concluded that patient safety incident and adverse event reporting lacked detail, and that successful advocacy affected outcomes, suggesting that when advocacy was ignored outcomes were worse. The review reported adverse event themes, including isolation due to limited methods to communicate with nurses, and that carers had a protective role in uncovering or preventing adverse events. Two primary studies reported within the aforementioned integrative review warrant further attention.[14 35] Firstly, a mixed-methods study concluded that hospitals often lack effective systems for identifying patients which makes monitoring safety incidents difficult. This study also highlighted that staff do not always readily identify patient safety issues or report them, with incident reports commonly focussed on events causing immediate or potential physical harm, and that safety issues were mostly related to delays and omissions of care.[14] Secondly, a study underpinned by a conceptual framework on patient safety aimed to identify factors that promote and compromise the implementation of reasonably adjusted healthcare services for patients with intellectual disabilities. This study emphasised the importance of ward culture, staff attitudes and staff knowledge in ensuring that hospital services are accessible to vulnerable patients.[35]

A study assessing readmission found no significant difference in 30-day readmission rates for people with and without learning disabilities, but that 69% of readmissions of people with learning disabilities were potentially preventable,[36] and a study examining outcomes and toxicity of chemotherapy for acute lymphoblastic leukaemia in children with Down Syndrome found that these patients spent more days in hospital particularly during the induction phase of treatment.[37]

In a mixed-methods study, staff survey respondents reported feeling less confident about managing challenging behaviour and always delivering safe care to children and young people with learning disabilities, compared to children and young people without learning disabilities, as well as reporting that the environment was less safe for meeting the needs of children and young people with learning disabilities compared to those without.[38]

A wealth of grey literature further evidenced vulnerabilities in terms of adverse events, quality of care, and patient safety for people with learning disabilities.[9 11 39 40 41 42 43 44 45 46 47 48 49 50 51 52] This included influential reports such as the 2013 Confidential Inquiry into Premature Deaths of People with Learning Disabilities (CIPOLD)[9] and the subsequent Learning Disabilities Mortality Review (LeDer) programme annual reports, which evaluate the LeDer programme.[11 45 46 47]

For peer review only - http://bmjopen.bmj.com/site/about/guidelines.xhtml

Table 1 An overview of articles relating to adverse events, patient safety and quality of care.

Author and year	Aim and method/article type	Key findings
Mimmo, Harrison and Hinchcliff (2018)[15]	Narratively synthesise evidence concerning the experience of iatrogenic harm during hospitalisation for children with ID<br>*Systematic review and narrative synthesis*	16 papers provided evidence around: the assumptions of HCWs; reliance on parental presence; and the need for HCWs to understand the IDs experienced by children in their care. There are specific aspects of hospitalisation that expose children with ID to harms that are preventable, avoidable and not experienced to the same extent by children without ID
Best, Asaro and Curley (2019)[33]	To compare current analgesia and sedation management practices between critically ill children with pre-existing CI and critically ill neurotypical children, including possible indicators of therapeutic efficacy<br>*Secondary analysis of prospective data*	CI patients received significantly lower doses of analgesia and sedation medication than those without CI. However, it was unclear if this was due to lower requirements or vulnerabilities to inadequate assessment
Hemsley et al (2016)[34]	Identify research reports regarding investigating the care or safety of adults with communication disabilities in hospital, and to analyse findings according to the generic model of patient safety<br>*Literature review*	Patient safety incident and adverse event reporting lacked detail e.g. little demographic, descriptive, temporal and categorical information about the patient and staff and how events were detected. Successful advocacy affected outcomes, although where advocacy was ignored outcomes were worse. Stories of adverse events themes included; suffering, isolation due to not having a method to communicate with nurses, a perilous care situation culminating in an adverse event and protective carers discovering or forestalling an adverse event
Kelly et al (2016)[36]	Compare 30-day hospital readmission rates of people with and without LDs<br>*Retrospective audit*	No significant difference in 30-day readmission rates for patients with and without LDs. However, 69% of readmissions of those with LDs were potentially preventable. Those with more profound LDs were at greater risk of experiencing poor quality care and experiencing readmission within 30 days, and this group comprised over half of the PPRs
Shah et al (2009)[37]	Review outcomes and toxicity of chemotherapy for acute lymphoblastic leukaemia in children with DS<br>*Cohort*	Patients with DS spent more days in hospital, particularly during the induction phase of treatment

For peer review only - http://bmjopen.bmj.com/site/about/guidelines.xhtml

Oulton et al (2018)[38]	Compare and identify factors that facilitate and prevent children and young people with and without LDs and long term conditions from receiving equal access to high quality hospital care and services *Mixed methods*	Two key themes; national variation and staff uncertainty. Lack of knowledge about policies systems at an organisational level to support care of children and young people with LDs. Considerable variation between hospitals ranging from those appearing to have few or no systems, policies or practices in place specifically for this group, with partial systems, policies or practices in place and those with a cohesive and comprehensive level of provision. There was a lack of standardised systems in place for communicating that an individual has a LD. Also a distinct lack of systems in place for recording that an individual involved in a complaint or the subject of clinical incident has a LD

Notes. CI = Cognitive Impairment, DS = Downs Syndrome, HCWs = Healthcare Workers, ID = Intellectual Disability, LD = Learning Disability, LDs = Learning Disabilities, PPRs = Potentially Preventable Readmissions.

For peer review only

For peer review only - http://bmjopen.bmj.com/site/about/guidelines.xhtml

Maternal and infant outcomes

Five articles examined maternal and infant outcomes utilising a retrospective and/or cohort design, either focussing on women with intellectual and developmental disabilities[53 54 55] intellectual disability and/or self reported learning difficulties[56] and attention deficit hyperactivity disorder.[57] Higher rates of complications such as pre-eclampsia[53 55 56 57] preterm birth[55 57] low birth weight[55 56] and labour interventions including induction and caesarean[53 55 57] were reported. One study reported higher prevalence rates for hospital admission and emergency department visits during all critical postpartum periods for those with intellectual and developmental disabilities, and higher risk of repeated hospitalisations.[54]

A survey led by Patient Experience Network (not-for-profit organisation) and CHANGE (national human rights organisation) supported by NHS England, aimed to capture the experience of parents with learning disabilities.[58] Training for health professionals to better support parents with learning disability and improving accessibility to services were highlighted as essential.

Post-operative outcomes

The post-operative experience featured significantly in the systematic review of the experience of iatrogenic harm during hospitalisation for children with intellectual disability included within this review (referred to in Objective 1 findings).[15] Thirteen further articles reported on post-operative outcomes.[59 60 61 62 63 64 65 66 67 68 69 70 71] The majority of articles included data relating to Down Syndrome,[59 60 61 63 64 65 68 69] followed by intellectual disability,[62 67 70] developmental delay,[66] and Autism Spectrum Disorder.[71] Increased rates of complications[60 62 63 66 69 70] were reported in a number of studies. However, in one study comorbidities rather than Down Syndrome were a greater risk factor for complications when adjusting for other covariates,[59] and after propensity matching, another study also focussing on patients with and without Down Syndrome, found no significant variation regarding rates of postoperative complications.[64] Furthermore, one study focussing on risk factors for major complications related to percutaneous endoscopic gastrostomy placement in children concluded that when adjusting for other variables, ID was not a significant risk factor.[67]

A longer length of stay was reported in four studies[60 62 63 70] with one study reporting a similar length of stay for those with Down Syndrome compared to those without[65], and one study reporting that patients with Autism Spectrum Disorder had a shorter length of stay and were less likely to experience complications.[71] In one study mortality and major complication rates were lower for patients with Down Syndrome.[65] Similarly, further studies also focussing on Down Syndrome found mortality and medical complications to be significantly lower for patients with Down Syndrome with no significant differences in terms of surgical complications,[68] and lower odds of in-hospital death for patients with Down Syndrome when controlling for other factors such as risk category and premature birth.[61] In four studies no differences in mortality were reported,[62 63 64 66] and in one study children with intellectual disability had a higher risk of 30-day mortality compared to children with no intellectual disability.[70]

Objective 2 - Explore protective factors and potential explanatory mechanisms.

Role of family and carers

Reliance on parental presence as a protective factor from poor care quality was emphasised in the systematic review of the experience of iatrogenic harm during hospitalisation for

For peer review only - http://bmjopen.bmj.com/site/about/guidelines.xhtml

children with intellectual disability included within this review (referred to in Objective 1 findings).[15] Furthermore, a primary study included within an already included literature review[34] (reported in Objective 1) warranted further attention within this concept. The qualitative interview study explored paid carers' roles in supporting adults with developmental disability and complex communication needs and described how paid carers are often motivated by perceived responsibility for safety, well-being and communication, but that their role can sometimes be blurred with nursing and family carer roles.[72]

Five further articles highlighted the significant role of families and carers. A meta-narrative approach to understand the experience for the parent of a child with intellectual disability in hospital resulted in a synthesis of 11 studies. A working model for professional parent partnership was developed which reinforced the importance of hospital/multidisciplinary approaches to care centring on the child, understanding previous negative experiences and negotiating care, and shared learning to lessen reliance on parental presence.[73] A further review evaluated how hospital systems respond to adults with intellectual disability, their families and carers. Key themes included; individual fear of hospital encounters, reliance on paid family carers for basic needs and advocacy, responsibilities and staff knowledge, skills and attitudes.[22]

A key finding from a qualitative study with medical practitioners concluded that practitioners make limited use of "reasonable adjustments" and turned to caregivers to facilitate communication and manage behaviours likely to upset hospital routines.[74] A mixed methods study aiming to identify factors that affect carer involvement for people with intellectual disabilities in acute hospitals presented a model for clarifying carer involvement that sought to highlight the degree to which carers are 'workers' contributing to basic nursing care, and the degree to which carers are experts or non-experts.[75] The authors suggested that making these two aspects explicit might facilitate staff to understand carer contributions more comprehensively. Finally, a quantitative case note audit demonstrated poor performance across a range of elements of hospital care for people with learning disability.[6] One notable positive finding of the audit was that in most cases family or carers were involved in discharge planning.[6] However, the thoroughness of this was questioned as many carers were not signposted to an assessment of their needs prior to discharge.

In terms of grey literature, a doctoral thesis which investigated emergency healthcare from the perspective of the carers of people with learning disabilities, highlighted the relationship staff had with both service users and carers as fundamental to a high quality service.[76]

Understanding needs in hospital

Six articles had content relating to the needs of people with learning disabilities in hospital.[77 78 79 80 81 82] One article concluded that to ensure nurses do as much as possible to identify risk they must recognise prejudices and overcome them, develop further understanding of learning disabilities and acknowledge the rights of people with learning disabilities, and collaborate with carers and professionals.[78] Similarly, a literature review around communication, recognised the importance of collaborating effectively with carers, as well as access to personally held written health information, inter-agency communication, devoting time to communication, and access to communication tools and aids.[79] A literature review assessing evidence around the promotion of health, safety and welfare of adults with learning disabilities in acute care emphasised the importance of care provision,

For peer review only - http://bmjopen.bmj.com/site/about/guidelines.xhtml

communication, staff attitudes, staff knowledge, supporters and carers and the physical environment.[77] Crucially, communication was highlighted as a fundamental issue, such that people with learning disabilities often have difficulty communicating their needs. The literature review presented strategies and resources that may support this such as videos, accessible booklets, augmentative and alternative communication and pictures/symbols.

To help improve the inpatient experience of hospital patients with autism, a survey of parents and guardians with qualitative and quantitative items highlighted the need for an individualised approach to assess and accommodate needs.[81] This approach was taken in a case study that described the plan of care for a patient with moderate level of learning disability scheduled for a tonsillectomy. The report gave a specific example of how investing time to understand a patient's need can improve experience.[82] When the patient's details were being checked, the door knocked into the patient's chair as staff entered the room for equipment, and this exacerbated the patient's anxiety. This was acknowledged quickly and a do not disturb sign was placed on the door.

An article aiming to familiarise the paediatric nurse with autism and create a resource for successful inpatient treatment put forward key themes such as change is a challenge, consistent caregivers, safe environment, encouraging family involvement, ways of communicating, emotional triggers and reward systems and multidisciplinary team from admission.[80] Indeed, the NHS long term plan published in 2019,[83] emphasised that the whole NHS will improve its understanding of the needs of people with learning disabilities and autism, with plans in place for staff to receive training on supporting people with a learning disability and/or autism alongside the implementation of national learning disability improvement standards. Furthermore, the government response to the consultation on learning disability and autism training for health and care staff also published in 2019, underlined the importance of gaining a better understanding of how to ensure that patients and service users receive safe, effective and dignified care, and the need to equip those providing care with the necessary skills, knowledge and behaviours.[84]

The importance of staff being knowledgeable about the children they care for and their ID also featured in the systematic review of the experience of iatrogenic harm during hospitalisation for children with intellectual disability included within this review (referred to in Objective 1 and 2 findings).[15]

Objective 3 - Identify patient safety interventions, improvement initiatives, recommendations and examples of good practice.

Supporting initiatives, recommendations and good practice

Ten articles utilising diverse designs (including commentary/opinion pieces, qualitative methods, service improvement, discussion/special interest/featured articles and short reports), reported either examples of initiatives to support safe care for people with learning disabilities in hospital, or recommendations to support good practice (see table 2).[85 86 87 88 89 90 91 92 93 94] A qualitative content analysis of 60 documents mapped the content of existing hospital passports for people with intellectual disability and concluded that this approach can enhance safety and person-centred care, but acknowledged there is much variation between current hospital passports which may limit effectiveness.[89] Six articles provided specific examples of how to enhance good practice.[85 88 91 92 93 94] These included a commentary highlighting how hospital pharmacists can contribute to safety when supporting people with

For peer review only - http://bmjopen.bmj.com/site/about/guidelines.xhtml

intellectual disability in hospital,[85] a special interest/review article focusing on the pre-surgical needs of those with Down Syndrome and how patient safety can be optimised,[88]and an opinion piece/review presenting recommendations for the perioperative management of children with autism.[91] Additionally, a featured article presented how simulations can educate nurses to maintain safety when caring for patients with Autism Spectrum Disorder,[92] and a short report highlighted the importance of: reliable identification of children with intellectual disability; exploring indirect indicators of poor quality care; and consumer engagement and the voice of the child with intellectual disability.[93] Finally, a research/discussion article explored key issues in working with people with intellectual disabilities and provided methods to improve the care provided.[94]

Three articles described improvement work.[86 87 90] One project identified areas of risk for people with intellectual disability whilst in hospital, and developed and successfully implemented a rapid risk assessment tool to assess immediate and potential risk, identify risk reduction actions and develop appropriate care bundles.[90] The second project identified core tasks of a specialist learning disability team to improve patient care for those with learning disabilities, examples included; educating acute staff, developing training materials for staff and trainees, considering consent issues and facilitating community support before discharge.[87] A mixed methods study comprising literature review and improvement work, developed care plans and an educational module. After completing the module, there was an increase in nurses' confidence when caring for people with learning disabilities.[86]

Further initiatives, recommendations and good practice examples were identified in the grey literature.[95 96 97 98 99 100 101 102 103 104 105 106 107] For brevity, we provide further information and signpost to these resources in Appendix 4.

For peer review only

For peer review only - http://bmjopen.bmj.com/site/about/guidelines.xhtml

Table 2 An overview of articles relating to supporting initiatives, recommendations and good practice.

Author and year	Aim and method/article type	Key findings
Blair (2013)[94]	To explore key issues in working with people with IDs and how to minimise clinical risk and ensure care is provided in an appropriate, timely and lawful manner<br>*Research/discussion article*	Discussion and practice examples around the following areas: core reasonable adjustments; hospital passport; assessing a person's capacity to consent to treatment; involving people with IDs in improving services and safety; how to improve care for people with an ID and reduce clinical risks; and reducing clinical risk improving care
Flood (2017)[85]	Raise awareness of how hospital pharmacists can contribute to safety when supporting people with ID in hospital<br>*Commentary*	To help pharmacists ensure people with IDs receive reasonably adjusted quality care it is important that; pharmacists know that a patient has IDs, pharmacy staff are aware of general healthcare and specific medication-related issues, transitions of care are considered as they are particularly vulnerable for people with IDs and people with IDs require equitable care that is appropriate for their needs
Friese and Ailey (2015)[86]	Develop care plans and an educational module for nurses caring for patients with LDs<br>*Improvement project*	Key components of care plans were communication, a safe environment, enhancing patients' behaviour and cooperation with care, and carer involvement. Nurse educational module aimed to increase understanding of needs of LD patients, improve communication and prevent adverse events. After completing the education module analysis showed significant improvement in nurses' confidence when caring for patients with LDs
Glasby (2002)[87]	Explore how a specialist LD team aimed to improve patient care for those with LDs<br>*Improvement project*	Core tasks of LD team included: accompanying individuals to appointments, ensuring individuals understand what is going to happen in hospital, considering consent issues, liaising with wards to help them understand the person's needs, providing practical support and advocating for the person's needs in hospital, enabling carers to have a break, facilitating community support before discharge, following up after discharge to ensure that all needs are being met, educating acute staff and developing training materials for staff and trainees
Lewanda et al (2016)[88]	Optimise patient safety for children with DS by choosing the most appropriate setting and perioperative personnel, and to mitigate those risk factors amenable to intervention<br>*Special interest article/review*	Pre-surgical evaluations for children with DS should identify appropriate personal and equipment and focus on; combining 2+ compatible surgical procedures under one anaesthesia event, assessing for undiagnosed or residual heart disease and the presence of pulmonary hypertension, considering potential cervical spine instability, assess if patient is taking dietary supplements and having various options available for anaesthesia during surgery

For peer review only - http://bmjopen.bmj.com/site/about/guidelines.xhtml

McIntosh et al (2020)[92]	Address unintentional injuries (e.g. medication, sharps, physical injury, diet, and overstimulation) that an individual with ASD may experience while in a health care environment<br>*Featured article*	Simulations can educate nurses to maintain safety when caring for a patient with ASD in the professional environment. This article presents simulation ideas/activities around: medication, diet, environment, sharps, hypersensitivity, ASD routines, treatment, stimming behaviours, and crisis management
Mimmo et al (2020)[93]	Highlight areas that must be addressed to provide the foundation for measuring, understanding and enhancing equity in the quality of care for children with ID<br>*Short report*	The report highlights the importance of: 1) reliable identification of children with ID; 2) exploring indirect indicators of poor quality care; and 3) consumer engagement and the voice of the child with ID
Northway et al (2017)[89]	Map the content of existing hospital passports for people with ID to inform nursing practice and future research<br>*Qualitative - content analysis*	60 documents developed by provider organisations in the UK and Northern Ireland were reviewed and varied considerably in terms of length, title and content. Most frequent content included; Name, Level of communication (expression and understanding), Level of support required with nutrition, Mobility, Sleeping, Communication of pain and distress, Behaviour, Personal care, Allergies, Contact person. Patient and primary care information absent in some documents. Concerns it may give relatives or carers a false sense of security
Read, Johnson and Tristan (2012)[90]	Identify areas of risk for patients with ID whilst in hospital to develop a rapid risk assessment tool for use in an acute hospital to assess immediate and potential risk, identify risk reduction actions and develop appropriate care bundles<br>*Improvement project*	Implementation of the care bundles gave structure and clear evidence-based guidance to deliver the best care for those with IDs. There was a reduction in bed days, lowering the risk of adverse events occurring, saving money in bed days and readmission penalties
Vlassakova and Emmanouil (2016)[91]	Summarise experiences and recommendations for the perioperative management of children with autism<br>*Opinion piece/review*	Children with autism each display a unique behavioural profile. Collecting information about the patient in advance, establishing good rapport with the family, clear communication with all members of the perioperative team are key to success. Minimising perioperative stress, providing quiet environment, avoiding use of potential harmful medications assure smooth perioperative care and minimise adverse events

Notes. ASD = Autism Spectrum Disorder, DS = Downs Syndrome, ID = Intellectual Disability, IDs = Intellectual Disabilities, LD = Learning Disability, LDs = Learning Disabilities.

For peer review only - http://bmjopen.bmj.com/site/about/guidelines.xhtml

Discussion

To the authors' knowledge, this is the first scoping review to synthesise both the academic and grey literature focusing on hospital patient safety outcomes for people with learning disabilities. Whilst, as a narrative synthesis we are unable to state unequivocally the relationship between having learning disabilities and safety outcomes, our findings do suggest that there are multiple ways in which people with learning disabilities might experience poorer outcomes compared with people without. Our review demonstrates that there are inequalities and inequities for a range of specific patient safety outcomes including adverse events, quality of care, maternal and infant outcomes and post-operative outcomes. This disparity needs urgent attention. Nonetheless, we did identify a range of potential protective factors, such as the roles of family and carers and the extent to which health professionals are able to understand the needs of people with learning disabilities. Research has focussed on developing interventions and good practice guidance, yet this is predominantly accounted for within the grey literature, meaning that robust evidence is still needed.

Some poorer outcomes are likely through the 'direct effects' of having a learning disability, for example, the increased incidence of co-morbidities in children with learning disabilities accounted for the increased likelihood of post-operative complications in one study.[59] However, it is also abundantly clear that there are multiple 'indirect effects' of having learning disabilities that may amplify problems. The review highlighted the prevailing potential risk of inadequate systems to identify and flag people with a learning disability when they enter an acute hospital setting, and the knock on effect this can have on the ability to effectively monitor patient safety incidents for these patients.[14 93] Crucially, if patient identification and flagging and therefore patient safety incident monitoring is not fit for purpose, this creates a significant knowledge gap which greatly limits the development of much needed solutions to address patient safety issues.

Further principal issues likely to manifest in differential outcomes included problems with communication (e.g. patients to staff, staff to patients, intra- and inter-agency), staff attitudes, the role of family and carers, staff awareness and knowledge/training, and variation in the quality and level of healthcare received. These indirect effects fall squarely in the realm of quality and safety efforts, modifiable potentially through service redesign, increased resources, training, professional specialisation, and appropriate adaptation of practice. Promising interventions and good practice examples were identified such as risk assessment tools,[90] preoperative and perioperative management recommendations,[88 91] hospital passports,[89 94 95] and education modules.[86]

We explore these issues through a patient safety 'lens', and what is perhaps most striking about our findings, is their lack of novelty. One of the earliest national reports within the UK – 'Healthcare for all'[41] – found similar issues, and made a series of recommendations. It is clear from our review that since this report, very little has changed in terms of the experience of people with learning disabilities and their families within acute care settings, either nationally or internationally. The exploration of this issue as a '*patient safety problem'* allows us to understand how, through the design of our healthcare system we create – and seek to solve – safety problems from the perspective of those moving through and navigating the system.

For peer review only - http://bmjopen.bmj.com/site/about/guidelines.xhtml

In an unrelated study, Fylan and colleagues examined the medicines management system for heart failure patients discharged from hospital into the community, and developed a framework called 'Gaps, Traps, Bridges and Props' which may be useful when thinking about our review findings.[108] '*Gaps'* occur in our systems at points of discontinuity or transition, and evidence from across patient safety literature that gaps in the structure and design of services create 'safety gaps' that present opportunities for problems for patients, especially when care is suboptimal or fails.[109 110] It is arguable that those patients with complex needs or specific vulnerabilities that require greater continuity of care, are more at risk when crossing these 'safety gaps' – in effect, their vulnerability amplifies the risk of experiencing a patient safety problem. In our review, it is evident that people with learning disabilities may disproportionately suffer due to these gaps in healthcare systems. Examples of this would include poor inter agency communication,[79] and hospitals lacking effective systems for identifying and flagging patients.[14] Sometimes, the design of services/organisations goes beyond creating a '*gap'* – which may or may not result in a safety problem for patients. '*Traps'* are here defined as features of system design that *actively* make problems more likely. An example of a '*trap'* from our review is the need for training on learning disabilities for healthcare staff.[49 83 84] Without specific knowledge of, and training in caring for those with a range of learning disabilities, it is perhaps understandable that staff regularly fail to make reasonable adjustments to accommodate specific needs.[74]

This framing provides the possibility to ameliorate the issues that result, either through formalised '*bridges*', or further supporting the range of informal '*props'* that serve to reduce problems when care is suboptimal, or fails. '*Bridges'* are viewed as formalised features of a system, designed to span service gaps, and support continuity of care.[108] We found a number examples – from patient-held passports,[89 94 95] to specialist learning disability teams.[87] However, our review also found that these '*bridges'* are often inconsistently available or applied, a position that could further amplify problems if staff have come to rely on them for support when needed. The most prevalent mechanism for supporting patients with learning disabilities came through the role of patients and carers. Although the need to reduce ambiguity about the role of the parent[73] and the importance of clarifying what carer involvement includes[75] were emphasised, we found a range of evidence that suggested families and carers regularly '*prop'* up services – from help with feeding and personal care,[22] to facilitating communication[74] and being involved in discharge planning[6] – and that without this '*prop'*, the outcomes for patients with learning disabilities may well be poorer.

Implications

Our review demonstrates the piecemeal and wide-ranging nature of the extant evidence, in terms of specific learning disabilities and outcomes of interest, and with a range of methodologies used. Therefore, we propose that research is needed to establish the burden of harm for people with learning disabilities as a result of patient safety incidents and poor quality of care, in hospital settings. This goes beyond learning from deaths – we need to understand what happens with care for people with learning disabilities more generally. Second, research needs to understand the mechanisms through which these effects might be seen. It is this approach that holds significant promise from the point of view of service improvement and redesign, as well as training and curriculum development. Put simply, we cannot change what we do not yet fully understand. Finally, attention must be given again to the existing recommendations from the range of reports already published. For example, common recommendations across many previous reports include: the need for better

For peer review only - http://bmjopen.bmj.com/site/about/guidelines.xhtml

systems to identify people with learning disabilities in healthcare settings;[9 39 41 46] the need for improved communication and information sharing between agencies and providers;[9 46 47] and the need for education and training in caring for people with learning disabilities.[11 39 41 46 47 49 83 84] There is already a wealth of learning about the problems that exist for people with learning disabilities and their families, what is needed now is policy level action.

Limitations

Despite an inclusive search strategy, relevant articles may not have been identified if they were not available in the sources searched. Additionally, due to the nature of the review, we did not conduct formal quality assessments and were therefore unable to make reflections and comparisons of article quality.

Conclusion

The academic and grey literature indicates that whilst in hospital, people with learning disabilities might experience poorer patient safety outcomes. The involvement of family and carers, and understanding the needs of people with learning disabilities in hospital were highlighted as potential protective factors. Many promising interventions and examples of good practice exist, however these may not be widely available or have been applied inconsistently.

For peer review only

Acknowledgements:
We are extremely grateful for the contributions from a number of people and groups. Thank you to the Yorkshire Quality and Safety Research (YQSR) Group patient panel and NIHR YH PSTRC Citizen Participation Group members for their contributions to the search strategy. Secondly, thank you to Amanda Mckie, Beth Fylan, Jonathan Benn, Kate Pickett, Steph Prady and Federica Bianchini for their contributions to the protocol draft and search strategy. Finally, thank you to Sara Ryan and John Baker for their feedback on the draft manuscript.

Data availability statement:
No additional data available.

Ethics statement
This scoping review did not involve human participants or unpublished secondary data. Therefore, approval from a research ethics committee was not required.

For peer review only

For peer review only - http://bmjopen.bmj.com/site/about/guidelines.xhtml

References

1 Arcaya MC, Arcaya AL, Subramanian SV. Inequalities in health: definitions, concepts, and theories. Global health action. 2015 Dec 1;8(1):27106.

2 Marmot M, Bell R. Fair society, healthy lives. Public health. 2012 Sep 1;126:S4-10.

3 Marmot M. Health equity in England: the Marmot review 10 years on. Bmj. 2020 Feb 25;368.

4 Cookson R, Propper C, Asaria M, Raine R. Socio-economic inequalities in health care in England. Fiscal Studies. 2016 Sep;37(3-4):371-403.

5 Mattox EA. Identifying vulnerable patients at heightened risk for medical error. Critical Care Nurse. 2010 Apr;30(2):61-70.

6 Sheehan R, Gandesha A, Hassiotis A, Gallagher P, Burnell M, Jones G, Kerr M, Hall I, Chaplin R, Crawford MJ. An audit of the quality of inpatient care for adults with learning disability in the UK. BMJ open. 2016 Apr 1;6(4).

7 Department of Health. Valuing people: a new strategy for learning disability for the 21st century. A White Paper. London: HMSO; 2001.

8 Cluley V. From "Learning disability to intellectual disability"—Perceptions of the increasing use of the term "intellectual disability" in learning disability policy, research and practice. British Journal of Learning Disabilities. 2018 Mar;46(1):24-32.

9 Heslop P, Blair P, Fleming P, Hoghton M, Marriott A, Russ L. Confidential Inquiry into premature deaths of people with learning disabilities (CIPOLD). Bristol: Norah Fry Research Centre. 2013.

10 Primary Care Domain, NHS Digital. Health and Care of People with Learning Disabilities: Experimental Statistics: 2016 to 2017 [Internet]. NHS Digital. NHS; 2017 [cited 2020Nov14]. Available from: https://digital.nhs.uk/data-and-information/publications/statistical/health-and-care-of-people-with-learning-disabilities/health-and-care-of-people-with-learning-disabilities-experimental-statistics-2016-to-2017

11 University of Bristol Norah Fry Centre for Disability Studies. The Learning Disabilities Mortality Review (LeDeR) Programme. Annual Report. London: Healthcare Improvement Quality Partnership, 2020.

12 Simpson S. Creating accessible healthcare environments for people with autism. Nursing Times. 2020 Jan;116(1):47-9.

13 National Patient Safety Agency (Great Britain). Understanding the patient safety issues for people with learning disabilities. National Patient Safety Agency; 2004.

14 Tuffrey-Wijne I, Goulding L, Gordon V, Abraham E, Giatras N, Edwards C, Gillard S, Hollins S. The challenges in monitoring and preventing patient safety incidents for people with intellectual disabilities in NHS acute hospitals: evidence from a mixed-methods study. BMC health services research. 2014 Dec;14(1):432.

15 Mimmo L, Harrison R, Hinchcliff R. Patient safety vulnerabilities for children with intellectual disability in hospital: a systematic review and narrative synthesis. BMJ paediatrics open. 2018;2(1).

For peer review only - http://bmjopen.bmj.com/site/about/guidelines.xhtml

16 Peters M, Godfrey C, McInerney P, Soares C, Khalil H, Parker D. The Joanna Briggs Institute reviewers' manual 2015: methodology for JBI scoping reviews.

17 Moher D, Stewart L, Shekelle P. All in the family: systematic reviews, rapid reviews, scoping reviews, realist reviews, and more. Syst Rev. 2015; 4: 183.

18 Arksey H, O'Malley L. Scoping studies: towards a methodological framework. International journal of social research methodology. 2005 Feb 1;8(1):19-32.

19 Levac D, Colquhoun H, O'Brien KK. Scoping studies: advancing the methodology. Implementation science. 2010 Dec;5(1):69.

20 Tricco AC, Lillie E, Zarin W, O'Brien KK, Colquhoun H, Levac D, Moher D, Peters MD, Horsley T, Weeks L, Hempel S. PRISMA extension for scoping reviews (PRISMA-ScR): checklist and explanation. Annals of internal medicine. 2018 Oct 2;169(7):467-73.

21 Welch V, Petticrew M, Tugwell P, Moher D, O'Neill J, Waters E, White H, PRISMA-Equity Bellagio group. PRISMA-Equity 2012 extension: reporting guidelines for systematic reviews with a focus on health equity. PLoS Med. 2012 Oct 30;9(10):e1001333.

22 Iacono T, Bigby C, Unsworth C, Douglas J, Fitzpatrick P. A systematic review of hospital experiences of people with intellectual disability. BMC health services research. 2014 Dec 1;14(1):505.

23 Imms C, Adair B, Keen D, Ullenhag A, Rosenbaum P, Granlund M. 'Participation': a systematic review of language, definitions, and constructs used in intervention research with children with disabilities. Developmental Medicine & Child Neurology. 2016 Jan;58(1):29-38.

24 Panagioti M, Geraghty K, Johnson J, Zhou A, Panagopoulou E, Chew-Graham C, Peters D, Hodkinson A, Riley R, Esmail A. Association between physician burnout and patient safety, professionalism, and patient satisfaction: a systematic review and meta-analysis. JAMA internal medicine. 2018 Oct 1;178(10):1317-31.

25 Zegers M, Hesselink G, Geense W, Vincent C, Wollersheim H. Evidence-based interventions to reduce adverse events in hospitals: a systematic review of systematic reviews. BMJ open. 2016 Sep 1;6(9):e012555.

26 McGowan J, Sampson M, Salzwedel DM, Cogo E, Foerster V, Lefebvre C. PRESS peer review of electronic search strategies: 2015 guideline statement. Journal of clinical epidemiology. 2016 Jul 1;75:40-6.

27 Donaldson MS, Corrigan JM, Kohn LT, editors. To err is human: building a safer health system. National Academies Press; 2000 Apr 1.

28 Dunn K, Rydzewska E, Macintyre C, Rintoul J, Cooper SA. The prevalence and general health status of people with intellectual disabilities and autism co-occurring together: a total population study. Journal of Intellectual Disability Research. 2019 Apr;63(4):277-85.

29 O'Brien G, Pearson J. Autism and learning disability. Autism. 2004 Jun;8(2):125-40.

30 Mayes SD, Calhoun SL, Crowell EW. Learning disabilities and ADHD: Overlapping spectrum disorders. Journal of learning disabilities. 2000 Sep;33(5):417-24.

For peer review only - http://bmjopen.bmj.com/site/about/guidelines.xhtml

31 Popay J, Roberts H, Sowden A, Petticrew M, Arai L, Rodgers M, Britten N, Roen K, Duffy S. Guidance on the conduct of narrative synthesis in systematic reviews. A product from the ESRC methods programme Version. 2006 Apr 1;1:b92.

32 Campbell M, Katikireddi SV, Sowden A, McKenzie JE, Thomson H. Improving Conduct and Reporting of Narrative Synthesis of Quantitative Data (ICONS-Quant): protocol for a mixed methods study to develop a reporting guideline. BMJ open. 2018 Feb 1;8(2):e020064.

33 Best KM, Asaro LA, Curley MA, Wypij D, Allen GL, Angus DC, Ascenzi JA, Bateman ST, Borasino S, Bowens CD, Bysani GK. Sedation Management for Critically Ill Children with Pre-Existing Cognitive Impairment. The Journal of pediatrics. 2019 Mar 1;206:204-11.

34 Hemsley B, Georgiou A, Hill S, Rollo M, Steel J, Balandin S. An integrative review of patient safety in studies on the care and safety of patients with communication disabilities in hospital. Patient education and counseling. 2016 Apr 1;99(4):501-11.

35 Tuffrey-Wijne I, Goulding L, Giatras N, Abraham E, Gillard S, White S, Edwards C, Hollins S. The barriers to and enablers of providing reasonably adjusted health services to people with intellectual disabilities in acute hospitals: evidence from a mixed-methods study. BMJ open. 2014 Apr 1;4(4).

36 Kelly CL, Thomson K, Wagner AP, Waters JP, Thompson A, Jones S, Holland AJ, Redley M. Investigating the widely held belief that men and women with learning disabilities receive poor quality healthcare when admitted to hospital: a single-site study of 30-day readmission rates. Journal of Intellectual Disability Research. 2015 Sep;59(9):835-44.

37 Shah N, Al-Ahmari A, Al-Yamani A, Dupuis L, Stephens D, Hitzler J. Outcome and toxicity of chemotherapy for acute lymphoblastic leukemia in children with Down syndrome. Pediatric blood & cancer. 2009 Jan;52(1):14-9.

38 Oulton K, Gibson F, Carr L, Hassiotis A, Jewitt C, Kenten C, Russell J, Whiting M, Tuffrey-Wijne I, Wray J. Mapping staff perspectives towards the delivery of hospital care for children and young people with and without learning disabilities in England: a mixed methods national study. BMC health services research. 2018 Dec 1;18(1):203.

39 Royal Society for Mentally Handicapped Children and Adults. Treat Me Right!: Better Healthcare for People with Learning Disability. Mencap; 2004.

40 Royal Society for Mentally Handicapped Children and Adults. Death by indifference: Following up the Treat Me Right! report. Mencap; 2007.

41 Michael J. Healthcare for all: report of the independent inquiry into access to healthcare for people with learning disabilities. Independent Inquiry into Access to Healthcare for People with Learning Disabilities; 2008.

42 Local Government Ombudsman. Six Lives: The Provision of Public Services to People with Learning Disabilities. The Stationery Office London. 2009.

43 Department of Health. Six lives: Progress report on healthcare for people with learning disabilities. Department of Health; 2013.

44 Mencap. Hear my voice: healthcare. 2020. https://www.mencap.org.uk/get-involved/campaign-mencap/hear-my-voice/hear-my-voice-healthcare [illegible]

For peer review only - http://bmjopen.bmj.com/site/about/guidelines.xhtml

45 University of Bristol Norah Fry Centre for Disability Studies. The Learning Disabilities Mortality Review (LeDeR) Programme. Annual Report. London: Healthcare Improvement Quality Partnership, 2016.

46 University of Bristol Norah Fry Centre for Disability Studies. The Learning Disabilities Mortality Review (LeDeR) Programme. Annual Report. London: Healthcare Improvement Quality Partnership, 2017.

47 University of Bristol Norah Fry Centre for Disability Studies. The Learning Disabilities Mortality Review (LeDeR) Programme. Annual Report. London: Healthcare Improvement Quality Partnership, 2018.

48 McGowan P. Ask Listen Do: Oliver's Story [Internet]. NHS Choices. NHS; 2018 [cited 2020Nov15]. Available from: https://www.england.nhs.uk/blog/ask-listen-do-olivers-story/

49 NHS England, NHS Improvement. The NHS patient safety strategy: safer culture, safer systems, safer patients. 2019. https://www.england.nhs.uk/wp-content/uploads/2020/08/190708_Patient_Safety_Strategy_for_website_v4.pdf

50 INTERIM BULLETIN UNDIAGNOSED CARDIOMYOPATHY IN A YOUNG PERSON WITH AUTISM. Healthcare Safety Investigation Branch. 2019. https://www.hsib.org.uk/documents/139/hsib_interim_bulletin_undiagnosed_cardiomyopathy_autism.pdf

51 Care Quality Commission. Declare Your Care: People with learning disabilities. 2020. https://www.cqc.org.uk/help-advice/your-stories/declare-your-care-people-learning-disabilities

52 National Institute for Health Research. Better health and care for all. 2020. http://dx.doi.org/10.3310/themedreview-04326

53 Brown HK, Kirkham YA, Cobigo V, Lunsky Y, Vigod SN. Labour and delivery interventions in women with intellectual and developmental disabilities: a population-based cohort study. J Epidemiol Community Health. 2016 Mar 1;70(3):238-44.

54 Mitra M, Parish SL, Akobirshoev I, Rosenthal E, Simas TA. Postpartum hospital utilization among Massachusetts women with intellectual and developmental disabilities: a retrospective cohort study. Maternal and child health journal. 2018 Oct 1;22(10):1492-501.

55 Parish SL, Mitra M, Son E, Bonardi A, Swoboda PT, Igdalsky L. Pregnancy outcomes among US women with intellectual and developmental disabilities. American journal on intellectual and developmental disabilities. 2015 Sep;120(5):433-43.

56 McConnell D, Mayes R, Llewellyn G. Women with intellectual disability at risk of adverse pregnancy and birth outcomes. Journal of Intellectual Disability Research. 2008 Jun;52(6):529-35.

57 Poulton AS, Armstrong B, Nanan RK. Perinatal outcomes of women diagnosed with attention-deficit/hyperactivity disorder: an Australian Population-Based Cohort Study. CNS drugs. 2018 Apr 1;32(4):377-86.

58 Change and PEN. Hidden Voices of Maternity Parents With Learning Disabilities Speak Out. 2015. https://patientexperiencenetwork.org/wp-content/uploads/2019/10/Hidden-Voices-of-Maternity-Executive-Summary-FINAL-260815-2.pdf

For peer review only - http://bmjopen.bmj.com/site/about/guidelines.xhtml

59 Bartz-Kurycki MA, Anderson KT, Austin MT, Kao LS, Tsao K, Lally KP, Kawaguchi AL. Increased complications in pediatric surgery are associated with comorbidities and not with Down syndrome itself. Journal of Surgical Research. 2018 Oct 1;230:125-30.

60 Boylan MR, Kapadia BH, Issa K, Perfetti DC, Maheshwari AV, Mont MA. Down syndrome increases the risk of short-term complications after total hip arthroplasty. The Journal of arthroplasty. 2016 Feb 1;31(2):368-72.

61 Evans JM, Dharmar M, Meierhenry E, Marcin JP, Raff GW. Association between Down syndrome and in-hospital death among children undergoing surgery for congenital heart disease: a US population-based study. Circulation: Cardiovascular Quality and Outcomes. 2014 May;7(3):445-52.

62 Lin JA, Liao CC, Chang CC, Chang H, Chen TL. Postoperative adverse outcomes in intellectually disabled surgical patients: a nationwide population-based study. PLoS One. 2011 Oct 27;6(10):e26977.

63 Purifoy ET, Spray BJ, Riley JS, Prodhan P, Bolin EH. Effect of Trisomy 21 on Postoperative Length of Stay and Non-cardiac Surgery After Complete Repair of Tetralogy of Fallot. Pediatric Cardiology. 2019 Dec 1;40(8):1627-32.

64 Tóth R, Szántó P, Prodán Z, Lex DJ, Sápi E, Szatmári A, Gál J, Szántó T, Székely A. Down syndrome and postoperative complications after paediatric cardiac surgery: a propensity-matched analysis. Interactive cardiovascular and thoracic surgery. 2013 Oct 1;17(4):691-7.

65 Louis JD, Jodhka U, Jacobs JP, He X, Hill KD, Pasquali SK, Jacobs ML. Contemporary outcomes of complete atrioventricular septal defect repair: analysis of the Society of Thoracic Surgeons Congenital Heart Surgery Database. The Journal of thoracic and cardiovascular surgery. 2014 Dec 1;148(6):2526-31.

66 Pugely AJ, Martin CT, Gao Y, Ilgenfritz R, Weinstein SL. The incidence and risk factors for short-term morbidity and mortality in pediatric deformity spinal surgery: an analysis of the NSQIP pediatric database. Spine. 2014 Jul 1;39(15):1225-34.

67 Vervloessem D, van Leersum F, Boer D, Hop WC, Escher JC, Madern GC, de Ridder L, Bax KN. Percutaneous endoscopic gastrostomy (PEG) in children is not a minor procedure: risk factors for major complications. InSeminars in pediatric surgery 2009 May 1 (Vol. 18, No. 2, pp. 93-97). WB Saunders.

68 Cairo SB, Zeinali LI, Berkelhamer SK, Harmon CM, Rao SO, Rothstein DH. Down syndrome and postoperative complications in children undergoing intestinal operations. Journal of pediatric surgery. 2019 Sep 1;54(9):1832-7.

69 Graber TJ, Baskin PL, Soria C, Greenberg M, Gabriel RA, Brzenski A. An assessment of perioperative respiratory adverse events and difficult intubation in pediatric patients with Trisomy 21. Pediatric Anesthesia. 2021 Jan 23.

70 Huang SY, Chang CC, Lin CS, Yeh CC, Lin JA, Cherng YG, Chen TL, Liao CC. Adverse outcomes after major surgery in children with intellectual disability. Developmental Medicine & Child Neurology. 2021 Feb;63(2):211-7.

71 Printz JN, Mirkin KA, Hollenbeak CS, Carr MM. Morbidity after tonsillectomy in children with autism spectrum disorders. American journal of otolaryngology. 2019 Sep 1;40(5):667-72.

For peer review only - http://bmjopen.bmj.com/site/about/guidelines.xhtml

72 Hemsley B, Balandin S, Worrall L. Nursing the patient with developmental disability in hospital: Roles of paid carers. Qualitative Health Research. 2011 Dec;21(12):1632-42.

73 Mimmo L, Woolfenden S, Travaglia J, Harrison R. Partnerships for safe care: A meta-narrative of the experience for the parent of a child with Intellectual Disability in hospital. Health Expectations. 2019 Dec;22(6):1199-212.

74 Redley M, Lancaster I, Pitt A, Holland A, Thompson A, Bradley JR, Glover G, Thomson K, Jones S, Herbert B, Holme A. “Reasonable adjustments” under the UK's Equality Act 2010: An enquiry into the care and treatment to patients with intellectual disabilities in acute hospital settings. Journal of Applied Research in Intellectual Disabilities. 2019 Nov;32(6):1412-20.

75 Tuffrey-Wijne I, Abraham E, Goulding L, Giatras N, Edwards C, Gillard S, Hollins S. Role confusion as a barrier to effective carer involvement for people with intellectual disabilities in acute hospitals: findings from a mixed-method study. Journal of advanced nursing. 2016 Nov;72(11):2907-22.

76 Piper E. Investigating the experiences of people with learning disabilities in Accident & Emergency from a carer perspective [Doctor of Clinical Psychology]. University of Hull; 2008.

77 Bradbury-Jones C, Rattray J, Jones M, MacGillivray S. Promoting the health, safety and welfare of adults with learning disabilities in acute care settings: a structured literature review. Journal of Clinical Nursing. 2013 Jun;22(11-12):1497-509.

78 Brittle R. Managing the needs of people who have a learning disability. Nursing times. 2004;100(10):28-9.

79 Hemsley B, Balandin S. A metasynthesis of patient-provider communication in hospital for patients with severe communication disabilities: informing new translational research. Augmentative and Alternative Communication. 2014 Dec 1;30(4):329-43.

80 Jolly AA. Handle with Care: Top Ten Tips a Nurse Should Know Before Caring For a Hospitalized Child with Autism Spectrum Disorder. Pediatric nursing. 2015 Jan 1;41(1).

81 Kopecky K, Broder-Fingert S, Iannuzzi D, Connors S. The needs of hospitalized patients with autism spectrum disorders: a parent survey. Clinical Pediatrics. 2013 Jul;52(7):652-60.

82 Wilkinson S. Assessing the perioperative communication needs of a patient with learning disabilities: An holistic case study approach. Journal of perioperative practice. 2018 Oct;28(10):278-82.

83 NHS. The NHS long term plan. 2019. https://www.longtermplan.nhs.uk/

84 Department of Health and Social Care. Consultation outcome: Learning disability and autism training for health and care staff. 2019. https://www.gov.uk/government/consultations/learning-disability-and-autism-training-for-health-and-care-staff

85 Flood B. Safety of People with Intellectual Disabilities in Hospital. What Can the Hospital Pharmacist Do to Improve Quality of Care?. Pharmacy. 2017 Sep;5(3):44.

86 Friese T, Ailey S. Specific standards of care for adults with intellectual disabilities. Nursing Management. 2015 Mar 25;22(1).

For peer review only - http://bmjopen.bmj.com/site/about/guidelines.xhtml

87 Glasby AM. Meeting the needs of people with learning disabilities in acute care. British journal of nursing. 2002 Nov 28;11(21):1389-92.

88 Lewanda AF, Matisoff A, Revenis M, Harahsheh A, Futterman C, Nino G, Greenberg J, Myseros JS, Rosenbaum KN, Summar M. Preoperative evaluation and comprehensive risk assessment for children with D own syndrome. Pediatric Anesthesia. 2016 Apr;26(4):356-62.

89 Northway R, Rees S, Davies M, Williams S. Hospital passports, patient safety and person-centred care: A review of documents currently used for people with intellectual disabilities in the UK. Journal of clinical nursing. 2017 Dec;26(23-24):5160-8.

90 Read S, Johnson T. Rapid risk assessment in acute hospital for patients with intellectual disabilities. Advances in Mental Health and Intellectual Disabilities. 2012 Nov 16;6(6):321-32.

91 Vlassakova BG, Emmanouil DE. Perioperative considerations in children with autism spectrum disorder. Current opinion in anaesthesiology. 2016 Jun 1;29(3):359-66.

92 McIntosh CE, Thomas CM. Improving Safety of Patients With Autism Spectrum Disorder Through Simulation. Clinical Simulation in Nursing. 2020 Aug 1;45:1-5.

93 Mimmo L, Woolfenden S, Travaglia J, Harrison R. Creating equitable healthcare quality and safety for children with intellectual disability in hospital. Child: care, health and development. 2020 Sep;46(5):644-9.

94 Blair J. Everybody's life has worth–Getting it right in hospital for people with an intellectual disability and reducing clinical risks. Clinical Risk. 2013 May;19(3):58-63.

95 Kent A. Improving acute care of people with learning disabilities. Nursing Times. 2008 Feb 5;104(5):32-3.

96 Royal College of Nursing. Dignity in health care for people with learning disabilities. 2009. http://oxleas.nhs.uk/site-media/cms-downloads/RCN_Dignity_in_healthcare.pdf

97 Guidelines and Audit Implementation Network. Guidelines on Caring For People with a Learning Disability in General Hospital Setting. 2010. https://rqia.org.uk/RQIA/files/81/81662c46-b7bb-43a5-9496-a7f2d919c2a3.pdf

98 The Hillingdon Hospitals NHS Foundation Trust. Responding to the needs of people with learning disabilities [Internet]; [cited 2020Nov16]. Available from: https://www.thh.nhs.uk/about/safety/learning_dis.php

99 Ailey S, Hart R. Comprehensive Program to Support Patients and Staff Improves Hospital Experience for Adult Patients With Intellectual and Developmental Disabilities [Internet]. AHRQ Innovations Exchange: Innovations and Tools to Improve Quality and Reduce Disparities. Agency for Healthcare Research and Quality; 2010 [cited 2020Nov16]. Available from: https://www.innovations.ahrq.gov/profiles/comprehensive-program-support-patients-and-staff-improves-hospital-experience-adult

For peer review only - http://bmjopen.bmj.com/site/about/guidelines.xhtml

100 Public Health Nursing Division, Department of Health in partnership with the Modernising
Learning Disabilities Nursing UK Implementation Group. Strengthening the Commitment: One year on: Progress report on the UK Modernising Learning Disabilities Nursing Review. 2014. https://assets.publishing.service.gov.uk/government/uploads/system/uploads/attachment_data/file/309153/Strengthening_the_commitment_one_year_on_published.pdf

101 Bowness B. Improving general hospital care of patients who have a learning disability. 2014. http://www.1000livesplus.wales.nhs.uk/sitesplus/documents/1011/How to %2822%29 Learning Disabilites Care Bundle web.pdf

102 Public Health England. Working together 2: Easy steps to improve support for people with learning disabilities in hospital Guidance for hospitals, families and paid support staff. 2014. http://cdn.basw.co.uk/upload/basw_14709-8.pdf

103 National Quality Board. National Guidance on Learning from Deaths. A Framework for NHS Trusts and NHS Foundation Trusts on Identifying, Reporting, Investigating and Learning from Deaths in Care. 2017. https://www.england.nhs.uk/wp-content/uploads/2017/03/nqb-national-guidance-learning-from-deaths.pdf

104 NHS England. Ask Listen Do Resource for Organisations and Practitioners. 2018. https://www.england.nhs.uk/wp-content/uploads/2018/10/ask-listen-do-for-organisations-and-practitioners-v1.pdf

105 Mencap and The National Autistic Society: Treat me well campaign. The Oliver McGowan mandatory training in learning disability and autism [Internet]. Mencap; 2018 [cited 2020Nov16]. Available from: https://www.mencap.org.uk/get-involved/campaign-mencap/treat-me-well/announcement-learning-disability-training-health-and

106 NHS Improvement. The learning disability improvement standards for NHS trusts. 2018. https://www.england.nhs.uk/wpcontent/uploads/2020/08/v1.17_Improvement_Standards_added_note.pdf

107 The Westminster Commission on Autism. A Spectrum of Obstacles An Inquiry into Access to Healthcare for Autistic People. 2016. https://westminsterautismcommission.files.wordpress.com/2016/03/ar1011_ncg-autism-report-july-2016.pdf

108 Fylan B, Marques I, Ismail H, Breen L, Gardner P, Armitage G, Blenkinsopp A. Gaps, traps, bridges and props: a mixed-methods study of resilience in the medicines management system for patients with heart failure at hospital discharge. BMJ open. 2019 Feb 1;9(2):e023440.

109 O'Hara JK, Aase K, Waring J. Scaffolding our systems? Patients and families 'reaching in'as a source of healthcare resilience. BMJ Qual Saf. 2019; 28 (1): 3–6.

110 O'Hara JK, Baxter R, Hardicre N. 'Handing over to the patient': A FRAM analysis of transitional care combining multiple stakeholder perspectives. Applied Ergonomics. 2020 May 1;85:103060.

For peer review only - http://bmjopen.bmj.com/site/about/guidelines.xhtml

Figure 1. PRISMA flow diagram.

For peer review only

For peer review only - http://bmjopen.bmj.com/site/about/guidelines.xhtml

For peer review only

For peer review only - http://bmjopen.bmj.com/site/about/guidelines.xhtml

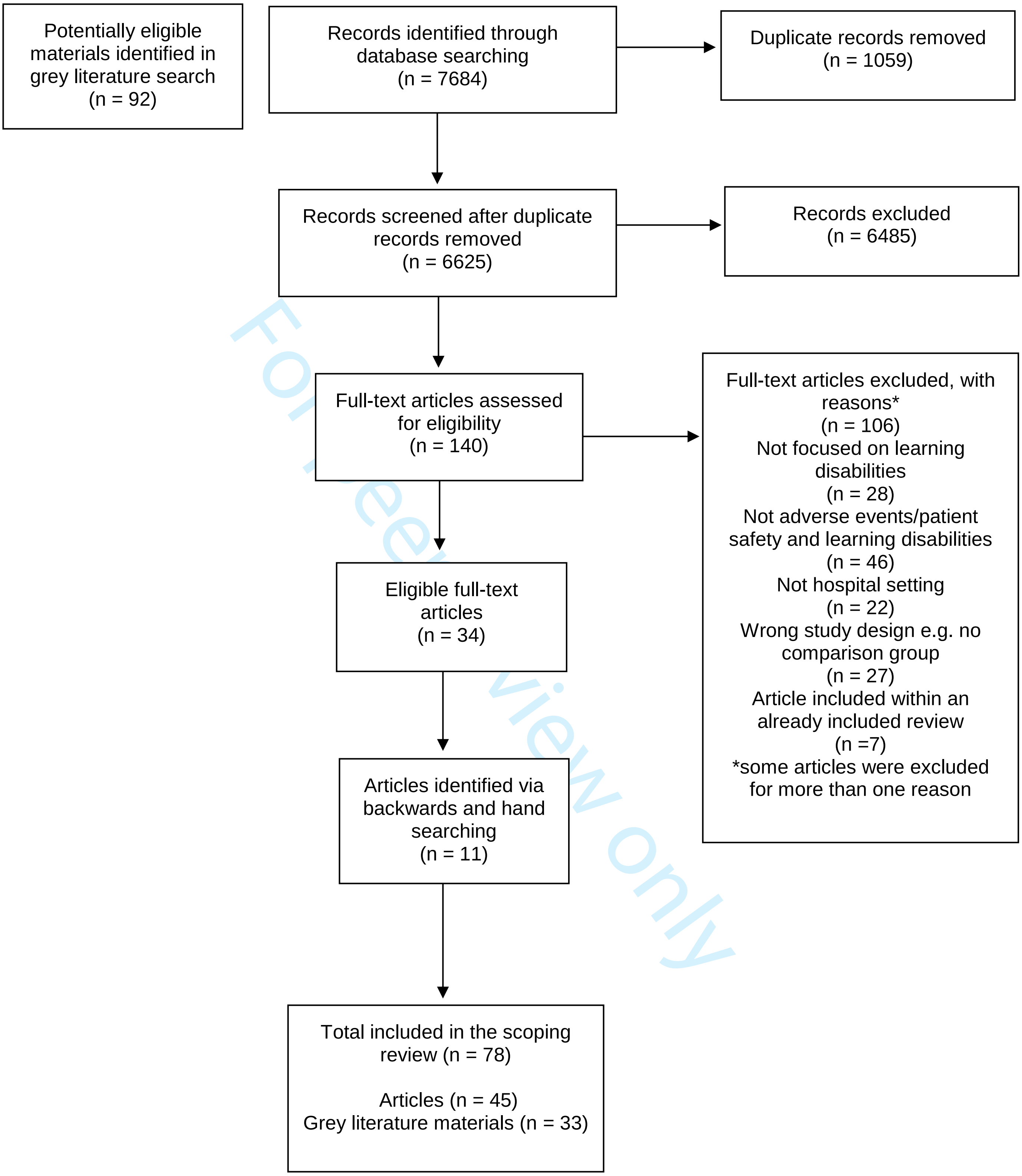

Figure 1. PRISMA flow diagram.

For peer review only - http://bmjopen.bmj.com/site/about/guidelines.xhtml

Appendix 1 MEDLINE search terms

**Learning disability (Population)**
Learning disabilit* .mp.
Intellectual disabilit* .mp.
Statemented .mp.
Communication disorder*1 .mp.
Communication impairment*1 .mp.
Complex communication needs .mp.
Intellectual* handicap* .mp.
Intellectual impairment*1 .mp.
Learning difficult* .mp.
Non?speaking .mp.
Mental handicap .mp.
Asperger*1 .mp.
Down syndrome .mp.
Complex need*1 .mp.
Autis* .mp.
Dyslexi* .mp.
Different need*1 .mp.
Altered need*1 .mp.
Sensory impair* .mp.
ADHD .mp.
Attention deficit .mp.
Hyperact* .mp.
Global development delay .mp.
Mental retardation .mp.
Cognition disorder* .mp.
Learning disorder* .mp.
Developmental disabilit* .mp.
Cognitive disabilit* .mp.
Cognitive impairment*1 .mp.
Communication disabilit* .mp.
**Adverse events and patient safety (Concept)**
Adverse event*1 .mp.
Sentinel event*1 .mp.
Near miss*2 .mp.
Close call*1 .mp.
Critical outcome*1 .mp.
Adverse outcome*1.mp.
Safety event*1 .mp.
Never event*1 .mp.
Serious incident*1 .mp.
Untoward incident*1 .mp.
Clinical incident*1 .mp.
Incident report*1 .mp.
Patient safety incident*1 .mp.
Safety incident*1 .mp.
Iatrogenic disease* .mp.
Medical error .mp.
Patient safety .mp.
Human error* .mp.
((adverse or avoidable or preventable or unsafe or safet*) ADJ2 (event* or outcome* or complication* or death* or effect* or reaction* or accident* or injur*)) .mp.
((medica* or diagnostic or therapeautic or administration or dispensing or prescri*) ADJ2 (error* or mistake* or fault*)) .mp.
(patient* ADJ2 (risk* or incident* or accident* or harm*)) .mp.
Near miss* .mp.
Never event* .mp.
Untoward incident* .mp.
Serious incident* .mp.

For peer review only

Serious report* event* .mp.<br>Medical error*1 .mp.<br>Iatrogenic .mp.
**Acute hospital setting (Context)**
Hospital*1 .mp.<br>Acute care .mp.<br>Secondary care .mp.<br>Tertiary care unit*1 .mp.<br>Ward*1 .mp.<br>Department*1 .mp.<br>In?patient*1 .mp.<br>Out?patient*1 .mp.<br>Triage*1 .mp.<br>Intensive care .mp.<br>Critical care .mp.<br>Urgent care .mp.<br>Internal medicine .mp.<br>A&E .mp.<br>Accident and emergency .mp.<br>Emergency care .mp.<br>Emergency medicine .mp.<br>Emergency treatment .mp.<br>Emergency admission .mp.<br>Hospitali#ation .mp.<br>Ambulatory care .mp.<br>Perioperative care .mp.<br>Preoperative care .mp.<br>Hospitali#ed .mp.<br>Perioperative .mp.<br>Preoperative .mp.<br>Postoperative .mp.<br>Re?operative .mp.<br>Post?operative .mp.<br>Admission .mp.<br>Casualty .mp.<br>Discharge .mp.<br>Emergency department .mp.

For peer review only

Appendix 2 Grey literature publicly available material searched

Mencap, NHS England, NHS Improvement, Healthcare Quality Improvement Partnership (HQIP), Healthcare Safety Investigation Branch (HSIB), The Kings Fund, Agency for Healthcare Research and Quality (AHRQ), Care Quality Commission (CQC), The Health Foundation, World Health Organisation, Institute of Healthcare improvement, Collaborations for Leadership in Applied Health Research and Care (CLAHRC), Nuffield Trust, Public Health England, The Office for National Statistics, The National Institute for Clinical Excellence, Mind, Learning Disability England, the British Institute of Learning Disabilities (BILD), Learning Disability Practice – RCNi, The National Research Center on Learning Disabilities (NRCLD), Royal College of Nursing, Royal College of Midwives, Nursing & Midwifery Council, Faculty of Dental Surgery, Faculty of Intensive Care Medicine,
Royal College of Anaesthetists, Royal College of Emergency Medicine, Royal College of Obstetricians and Gynaecologists, Royal College of Paediatrics and Child Health, Royal College of Physicians of Edinburgh, Royal College of Physicians of Ireland, Royal College of Physicians of London, Royal College of Physicians and Surgeons of Glasgow, Royal College of Surgeons in Ireland, Royal College of Surgeons of Edinburgh, Royal College of Surgeons of England.

For peer review only

Appendix 3 Included articles with author, year, country, aims, participants, methodology and key findings (presented in alphabetical order)

Author	Year	Country	Aims	Participants	Methodology	Key findings
Bartz-Kurycki et al[59]	2018	USA	Investigate whether DS is a risk factor for postoperative complications in paediatric patients undergoing gastrointestinal and non-cardiac thoracic surgery, and determine factors associated with complications	Total: 91,478 patients <18y old who underwent gastrointestinal or non-cardiac thoracic surgery. With DS: 1,476 (1.6%)	Quantitative: Retrospective cohort study using univariate analysis and multivariate logistic regression	DS patients had significantly higher postoperative complication rates than controls. However, comorbidities rather than DS were a greater risk factor for complications
Best, Asaro and Curley[33]	2019	USA	To compare current analgesia and sedation management practices between critically ill children with pre-existing cognitive impairment and critically ill neurotypical children, including possible indicators of therapeutic efficacy	Total: 2,449 patients 2 weeks – 17y old were included who underwent invasive mechanical ventilation for acute airway and/or parenchymal lung disease.<br>With CI: 412	Quantitative: Secondary analysis of prospective data using linear, cumulative logit, logistic, multinomial logistic, proportional hazards, and Poisson regression	CI patients received significantly lower doses of analgesia and sedation medication than those without CI. However, it was unclear if this was due to lower requirements or vulnerabilities to inadequate assessment
Blair[94]	2013	UK	To explore key issues in working with people with IDs and how to minimise clinical risk and ensure care is provided in an appropriate, timely and lawful manner	A guide about how to get things right for people with IDs with examples from practice	Research /discussion article	Discussion and practice examples around the following areas: core reasonable adjustments; hospital passport; assessing a person's capacity to consent to treatment; involving people with IDs in improving services and safety; how to improve care for people with an ID and reduce clinical risks; and reducing clinical risk improving care

For peer review only - http://bmjopen.bmj.com/site/about/guidelines.xhtml

Author	Year	Country	Aims	Participants	Methodology	Key findings
Boylan et al[60]	2016	USA	Assess and compare short-term outcomes of total hip arthroplasty in patients with and without DS	Total: 543,085 patients who underwent total hip arthroplasty in 1998 – 2010. With DS: 241 (0.04%)	Quantitative: Retrospective cohort study using logistic regression modelling, independent sample t-tests and linear regression	DS patients had significantly increased rates of medical, surgical and any complication compared to matched controls. This included pneumonia, urinary tract infection and wound haemorrhage. Patients with DS also had a longer mean length of stay
Bradbury-Jones et al[77]	2013	UK	Review evidence regarding the promotion of health, safety and welfare of adults with LD in acute care	Studies focussed on adults with LDs	Literature review	Six areas of influence on the health, safety and welfare of adults with LDs in acute hospitals were identified: care provision; communication; staff attitudes; staff knowledge; supporters and carers; and physical environment
Brittle[78]	2004	UK	Consider how nurses may deal with increasing numbers of LD patients accessing generic health services including hospitals	Studies including people with LDs accessing generic health services	Discussion article	To ensure that nurses do as much as possible to recognise risk when caring for people with LDs, they must; recognise any prejudices and overcome them, acknowledge that people with LDs have the same rights to healthcare as others, develop further understanding of LDs and collaborate with carers and professionals
Brown et al[53]	2016	Canada	Compare the occurrence of labour induction, c-section and operative vaginal delivery in women with and without IDD and determine whether pre-pregnancy health conditions or pregnancy complications explain any differences	Total: 386,706 deliveries to 263,284 women. With IDD: 3,932 deliveries to 2,584 women Without IDD: 382,774 deliveries to 260,700 women	Quantitative: Retrospective cohort study using Poisson regression and mediation analysis	Women with IDD were younger, lived in lower income areas and had higher rates of pre-pregnancy health conditions including: pre-existing diabetes mellitus, herpes or HIV, epilepsy, and psychiatric disorders. They had higher rates of complications such as pre-eclampsia and fetal death, and were more likely to have labour interventions such as induction and caesarean. Psychiatric disorders were the most important variable for labour induction and caesarean

Author	Year	Country	Aims	Participants	Methodology	Key findings
Cairo et al[68]	2019	USA	To measure medical and surgical complications, as well as in-patient mortality, in pediatric patients with DS undergoing intestinal operations and to compare these to patients without DS	Total: 17,026 pediatric patients undergoing intestinal operations. With DS: 444 (2.6%)	Quantitative: Cohort study using multivariable regression model	Mortality and medical complications were significantly lower for patients with DS. Surgical complications were not significantly different between patients with and without DS
Evans et al[61]	2014	USA	Identify differences in in-hospital mortality after cardiac surgery in paediatric patients with and without DS	Total: 51,309 patients <18y old who underwent surgery to correct congenital heart disease With DS: 4231 (8.2%)	Quantitative: Retrospective cohort study using multivariable logistic regression analysis	When controlling for risk category, premature birth, presence of ≥1 major non-cardiac structural anomalies, and age, a diagnosis of DS was associated with a lower odds of in-hospital death
Flood[85]	2017	UK	Raise awareness of how hospital pharmacists can contribute to safety when supporting people with ID in hospital	Discussing studies and policies focussing on people with ID in hospital	Commentary	To help pharmacists ensure people with IDs receive reasonably adjusted quality care it is important that; pharmacists know that a patient has IDs, pharmacy staff are aware of general healthcare and specific medication-related issues, transitions of care are considered as they are particularly vulnerable for people with IDs and people with IDs require equitable care that is appropriate for their needs

For peer review only

Author	Year	Country	Aims	Participants	Methodology	Key findings
Friese and Ailey[86]	2015	USA	Develop care plans and an educational module for nurses caring for patients with LDs	Nurses completed a pre-evaluation (n = 75) and post-evaluation (n = 99) questionnaire. Over 300 nurses completed the educational module	Mixed methods comprising: Literature review and PDSA cycles to develop care plans and educational module. Quantitative assessment of nurses' confidence in caring for patients with LD after completing the module using chi squared	Key components of care plans were communication, a safe environment, enhancing patients' behaviour and cooperation with care, and carer involvement. Nurse educational module aimed to increase understanding of needs of LD patients, improve communication and prevent adverse events. After completing the education module analysis showed significant improvement in nurses' confidence when caring for patients with LDs
Glasby[87]	2002	UK	Explore how a specialist LD team aimed to improve patient care for those with LDs	A specialist learning disability team of nursing and support staff set up to improve care for people with LDs	Improvement project: Observational	Core tasks of LD team included: accompanying individuals to appointments, ensuring individuals understand what is going to happen in hospital, considering consent issues, liaising with wards to help them understand the person's needs, providing practical support and advocating for the person's needs in hospital, enabling carers to have a break, facilitating community support before discharge, following up after discharge to ensure that all needs are being met, educating acute staff and developing training materials for staff and trainees

Author	Year	Country	Aims	Participants	Methodology	Key findings
Graber et al[69]	2021	USA	To determine the incidence of difficult intubation and perioperative respiratory adverse events in pediatric patients with trisomy 21 receiving general anesthesia compared to a matched control population	Total: 2649 patients. Trisomy 21 group: 1213 (47.8%)	Quantitative: Retrospective cohort study, chart review using logistic regression analysis	Patients with trisomy 21 experienced more perioperative respiratory adverse events compared to well-matched controls, largely attributable to the increased frequency of obstructed ventilation in trisomy 21 patients
Hemsley and Balandin[79]	2014	Australia	To synthesise the findings of research into communication in hospital for people with severe communication disabilities arising from lifelong and acquired stable conditions including cerebral palsy, autism, intellectual disability, aphasia following stroke, but excluding progressive conditions and those solely related to sensory impairments of hearing or vision	Studies including people with severe communication disabilities	Literature review – metasynthesis	The reviewed highlighted strategies to improve communication. 1) Develop services, systems and policies that support improved communication; 2) Devote enough time to communication; 3) Ensure adequate access to communication; 4) Access personally held written information; 5) Collaborate effectively with carers, spouses, and parents; and 6) Increase communicative competence of staff

For peer review only

Author	Year	Country	Aims	Participants	Methodology	Key findings
Hemsley et al[34]	2016	Australia	Identify research reports regarding investigating the care or safety of adults with communication disabilities in hospital, and to analyse findings according to the generic model of patient safety	Studies including hospital patients with communication disabilities	Literature review	Patient safety incident and adverse event reporting lacked detail e.g. little demographic, descriptive, temporal and categorical information about the patient and staff and how events were detected. Successful advocacy affected outcomes, although where advocacy was ignored outcomes were worse. Stories of adverse events themes included; suffering, isolation due to not having a method to communicate with nurses, a perilous care situation culminating in an adverse event and protective carers discovering or forestalling an adverse event
Huang et al[70]	2020	Taiwan	Evaluate outcomes after major surgery in children and adolescents with ID	Total: 21,730 patients.<br>With ID: 2173 (10%)	Quantitative: Nested cohort study using multiple logistic regression models	Children with ID had a higher risk of postoperative pneumonia, sepsis, and 30-day mortality compared with children without ID. Children with ID had longer lengths of hospital stay when compared with children with no ID
Iacono et al[22]	2014	Australia	Evaluate the evidence regarding how hospital systems respond to adults with ID, their families and carers	Studies focussed on people with ID or carers or staff perceptions and experiences of quality of care of people with ID	Systematic review and meta-synthesis	Themes included: individual fear of hospital encounters; failure of staff to provide care; staff knowledge and skills; staff attitudes; staff or system failures to adjust to needs; over reliance on paid and family carers for basic needs and advocacy; and enhancers to appropriate hospital care
Jolly[80]	2015	USA	Familiarise the paediatric nurse with autism and create a resource for successful inpatient treatment of a child with the disorder	Discussing knowledge of working with children with autism	Discussion article	Themes included; understanding autism, encouraging family involvement, best way of communicating, change is a challenge for children with autism, consistent caregivers, safe environment, emotional triggers and reward systems, and MDT from admission

Author	Year	Country	Aims	Participants	Methodology	Key findings
Kelly et al[36]	2015	UK	Compare 30-day hospital readmission rates of people with and without LDs	Total: 66,870 patients. With LDs: 256 (0.38%)	Quantitative: Retrospective audit using chi-squared	No significant difference in 30-day readmission rates for patients with and without LDs. However, 69% of readmissions of those with LDs were potentially preventable. Those with more profound LDs were at greater risk of experiencing poor quality care and experiencing readmission within 30 days, and this group comprised over half of the PPRs
Kopecky et al[81]	2013	USA	Characterise the needs of hospital patients with autism in various categories via a survey to aid facilitating the inpatient experience and improve quality of care	80 parents and guardians of patients with autism	Mixed methods: Survey comprising qualitative and quantitative items	Those with autism have a wide range of challenges and needs including communication, social/pragmatic concerns, and sensory processing whilst in hospital. An individualised approach must be used to assess and accommodate needs. 50% of parents had safety concerns for their child's hospital stay including; wandering or elopement, self-injury or aggression, pica, and pulling out IV tubing
Lewanda et al[88]	2016	USA	Optimise patient safety for children with DS by choosing the most appropriate setting and perioperative personnel, and to mitigate those risk factors amenable to intervention	Specialist team presenting their knowledge on working with children with DS	Special interest article/review	Pre-surgical evaluations for children with DS should identify appropriate personal and equipment and focus on; combining 2+ compatible surgical procedures under one anaesthesia event, assessing for undiagnosed or residual heart disease and the presence of pulmonary hypertension, considering potential cervical spine instability, assess if patient is taking dietary supplements and having various options available for anaesthesia during surgery

For peer review only

Author	Year	Country	Aims	Participants	Methodology	Key findings
Lin et al[62]	2011	Taiwan	Clarify whether ID is an independent risk factor for in-hospital major surgeries, and to validate the postoperative adverse outcomes in patients with ID	Total: 3,983 patients with preoperative diagnosis of ID who underwent inpatient major surgeries in 2004 - 2007	Quantitative: Retrospective, (matched controls) study using descriptive statistics and multivariate logistic regression	Surgical patients with ID had significantly higher incidence of preoperative comorbidities and postoperative complications while consuming more medical resources than controls. They also had higher average length of hospital stay and use of intensive care, as well as higher rates of acute renal failure, pneumonia, postoperative bleeding septicaemia, stroke and any complications. Postoperative 30-day mortality showed no significant difference between patients with or without ID after adjusting for teaching hospital, low income, urbanization and coexisting disease
McConnell, Mayes and Llewellyn[56]	2008	Australia	Explore the prevalence of poor pregnancy and birth outcomes in women with ID and/or self-reported learning difficulties in an antenatal population	Total: 834 women who gave birth to 839 children. With ID and/or self-reported learning difficulties: 54 Without ID and/or self-reported learning difficulties: 780	Quantitative: Cohort study using descriptive statistics, odds ratios with confidence intervals	Women with ID and/or self-reported learning difficulties had significantly higher prevalence of pre-eclampsia and a higher rate of low birthweight. They also had higher rates of pre-term delivery and high birth weight, but these differences were not statistically significant
McIntosh et al[92]	2020	USA	Address unintentional injuries (e.g. medication, sharps, physical injury, diet, and overstimulation) that an individual with ASD may experience while in a health care environment	Presenting simulation ideas/activities	Featured article	Simulations can educate nurses to maintain safety when caring for a patient with ASD in the professional environment. This article presents simulation ideas/activities around: medication, diet, environment, sharps, hypersensitivity, ASD routines, treatment, stimming behaviours, and crisis management

For peer review only

Author	Year	Country	Aims	Participants	Methodology	Key findings
Mimmo et al[93]	2020	Australia	Highlight areas that must be addressed to provide the foundation for measuring, understanding and enhancing equity in the quality of care for children with ID	Presents areas to be addressed based on a larger programme of work	Short report	The report highlights the importance of: 1) reliable identification of children with ID; 2) exploring indirect indicators of poor quality care; and 3) consumer engagement and the voice of the child with ID
Mimmo et al[73]	2019	Australia	Identify evidence regarding the parental experience of hospitalisation with a child with ID and care quality and safety	Studies focussed on parents or carers of children who are inpatients with ID	Literature review: scoping review and meta-synthesis	Findings from 11 studies were consolidated into five themes; being more than a parent, importance of role negotiation to reduce ambiguity about the role of the parent, building trust and relationships through effective communication, the cumulative effect of previous experiences of hospitalisation and healthcare staff taking time to know the child as an individual. Partnerships in care are vital to deliver safe care for children with ID
Mimmo, Harrison and Hinchcliff[15]	2018	Australia	Narratively synthesise evidence concerning the experience of iatrogenic harm during hospitalisation for children with ID	Studies focussed on child inpatients with ID	Systematic review and narrative synthesis	16 papers provided evidence around: the assumptions of HCWs; reliance on parental presence; and the need for HCWs to understand the IDs experienced by children in their care. There are specific aspects of hospitalisation that expose children with ID to harms that are preventable, avoidable and not experienced to the same extent by children without ID

For peer review only

Author	Year	Country	Aims	Participants	Methodology	Key findings
Mitra et al[54]	2018	USA	Compare the risk of postpartum hospital admission and emergency department visits during the first postpartum year among women with and without IDD	Total: 779,513 deliveries by women who gave birth in 2002 - 2012. Mothers with IDD: 1,104 Mothers without IDD: 778,409	Quantitative: Retrospective cohort study using chi-square, t-tests and Wilcoxon rank-sum tests	Women with IDD had higher prevalence rates for hospital admission and emergency department visits during all critical postpartum periods than those without IDD, with at least 2x higher rates for any hospitalisations within 1–42, 43–90, and 91–365 days after childbirth. Women with IDD also had a higher risk for repeated hospitalisations
Northway et al[89]	2017	UK	Map the content of existing hospital passports for people with ID to inform nursing practice and future research	Review of 60 hospital passports	Qualitative: content analysis.	60 documents developed by provider organisations in the UK and Northern Ireland were reviewed and varied considerably in terms of length, title and content. Most frequent content included; Name, Level of communication (expression and understanding), Level of support required with nutrition, Mobility, Sleeping, Communication of pain and distress, Behaviour, Personal care, Allergies, Contact person. Patient and primary care information absent in some documents. Concerns it may give relatives or carers a false sense of security
Parish et al[55]	2015	USA	Explore and compare both the pregnancy outcomes of women with and without IDD, and health outcomes of children born to mothers with and without IDD	Total: 3,859,539 pregnant women. Women with IDD: 1,706 Women without IDD: 3,857,833	Quantitative: Retrospective study using chi-square and logistic regressions	Women with IDD were more likely to have a c-section and a longer hospital stay. They were also more likely to experience adverse pregnancy outcomes such as preeclampsia, preterm birth, early labour and their infants were more likely to have a low birth weight

For peer review only

Author	Year	Country	Aims	Participants	Methodology	Key findings
Poulton, Armstrong and Nanan[57]	2018	Australia	Investigate the impact of ADHD and the effect of stimulant medication on women's perinatal outcomes	5,056 women treated with stimulants for ADHD<br>25,249 untreated women	Quantitative: Cohort study, multiple logistic regression	Women treated for ADHD with stimulants at any time (before, before and during, or only after the index pregnancy) had lower rates of spontaneous labour, and higher rates of caesarean delivery, active new-born resuscitation, and neonatal admission. 4 h. stimulant treatment for ADHD before or before and during pregnancy was also associated with higher rates of preeclampsia, preterm birth, and low 1-min Apgar score
Printz et al[71]	2019	USA	Examine whether outcomes differ between pediatric patients with and without ASD in a national cohort of children undergoing tonsillectomy	Total: 27,040 patients.<br>With ASD: 322 (1.2%)	Quantitative Retrospective cohort study using chi-square, t-tests, logistic regression, and generalized linear regression	After controlling for potential confounders, multivariable modeling suggested patients with ASD had a shorter length of stay and were less likely to experience complications
Purifoy et al[63]	2019	USA	Determine whether DS is associated with higher mortality, longer length of stay and greater incidence of gastrostomy and/or tracheostomy after complete repair of tetralogy of Fallot	Total: 4790 patients aged 1 day - 19y old<br>With DS 430 (8.9%)	Quantitative: Retrospective cohort study using chi-square	Patients with DS had longer mean postoperative length of stay after complete repair and greater incidence of postoperative gastrostomy than those without. However, no significant difference in mortality prior to discharge or rates of postoperative tracheostomy

For peer review only

Author	Year	Country	Aims	Participants	Methodology	Key findings
Read, Johnson and Tristan[90]	2012	UK	Identify areas of risk for patients with ID whilst in hospital to develop a rapid risk assessment tool for use in an acute hospital to assess immediate and potential risk, identify risk reduction actions and develop appropriate care bundles	Total: 54 rapid risk assessments with people with IDs admitted to the pilot wards	Improvement project: PDSA cycles	Implementation of the care bundles gave structure and clear evidence-based guidance to deliver the best care for those with IDs. There was a reduction in bed days, lowering the risk of adverse events occurring, saving money in bed days and readmission penalties
Redley et al[74]	2019	UK	Understand the views of qualified medical practitioners regarding reasonable adjustments and the quality of the care and treatment provided to adult inpatients with ID	Total: 14 medical practitioners	Qualitative: Interview study using thematic analysis	Medical practitioners focused on two accounts: the patients' communication difficulties and vulnerability to behaviours that did not conform to a hospital's expectations, and their biomedical complexities. They reported making limited use of “reasonable adjustments" and turned to caregivers to facilitate communication and manage behaviours likely to upset hospital routines
Shah et al[37]	2009	Canada	Review outcomes and toxicity of chemotherapy for acute lymphoblastic leukaemia in children with DS	30 patients with DS<br><br>60 patients without DS	Quantitative: Cohort study using Cox proportional hazards and a matched generalized linear model	Patients with DS spent more days in hospital, particularly during the induction phase of treatment

For peer review only

Author	Year	Country	Aims	Participants	Methodology	Key findings
Sheehan et al[6]	2016	UK	Examine steps that hospitals are taking to deliver high-quality care to people with a LD, and examine any impact these have on care quality	Total: 176 patients case note audit from adults with LDs who received inpatient hospital care in acute general and mental health services in May 2013 - April 2014	Quantitative: Clinical case note audit study using multivariable logistic regression	The strongest performance was in ensuring that family or carers were involved in discharge planning (84% evidencing this was enacted). Weight measurement or BMI was recorded in 58% notes. Compliance with all other audit criteria was <50%. Records of swallowing assessments, epilepsy risk assessment (for those with epilepsy) and that a health passport was used fared particularly badly, with evidence of these interventions in only 19%, 21% and 24% cases, respectively. For most quality indicators, there was a non-statistically significant trend for improved performance in services with a LD liaison nurse. The presence of an electronic flagging system showed less evidence of benefit
St Louis et al[65]	2014	USA	Descriptively analyse surgical outcomes from repair of complete atrioventricular septal defect in infants	Total: 2,399 patients who underwent surgery in 2008 - 2011.<br>With DS: 78.4%	Quantitative: Cohort study using Wilcoxon rank sum tests	Mortality and major complication rates were lower for patients with DS than for those without. Length of stay was similar
Toth et al[64]	2013	Hungary	Compare postoperative morbidity and mortality of paediatric patients with and without DS who underwent heart surgery	Patients <18y old who underwent heart surgery and were admitted to the cardiac ICU in 2003 - Dec 2008.<br>With DS: 129<br>Without DS: 1667<br><br>After propensity matching<br>With DS: 111<br>Without DS: 111	Quantitative: Retrospective cohort study using $\chi 2$-test, Fisher's exact test and t-tests using a non-parsimonious multivariable logistic regression model	After propensity matching, there was no significant variation between the groups regarding rates of postoperative complications or mortality

For peer review only

Author	Year	Country	Aims	Participants	Methodology	Key findings
Tuffrey-Wijne et al[75]	2016	UK	Identify factors that affect carer involvement for people with ID in acute hospitals and develop guidance to promote effective carer involvement	Survey response from 990 staff and 88 carers. Interviews with 68 hospital staff and 37 carers	Mixed methods	High staff awareness of good practice. Carers generally satisfied with how they were treated. A significant minority were dissatisfied on factors including expectations to provide basic nursing care, their expertise not being acted upon and discrepancies in perspective on the role of carers. A new model for clarifying carer involvement includes; the degree carers are 'workers' contributing to basic nursing care the degree carers are experts or non-experts
Vlassakova and Emmanouil[91]	2016	USA	Summarise experiences and recommendations for the perioperative management of children with autism	Studies focussed on children with autism	Opinion piece/review	Children with autism each display a unique behavioural profile. Collecting information about the patient in advance, establishing good rapport with the family, clear communication with all members of the perioperative team are key to success. Minimising perioperative stress, providing quiet environment, avoiding use of potential harmful medications assure smooth perioperative care and minimise adverse events
Wilkinson[82]	2018	UK	Produce a case study detailing the plan of care for an 18 year old male patient with a moderate level of LD who was scheduled for a tonsillectomy in hospital	Focussing on an 18 year old male patient with LD	Case study	Through collaboration and effective communication between practitioners, anaesthetists, surgeon and recovery care staff, the patient was admitted for his procedure with full knowledge of his individual needs and concerns, despite his limited communication skills. The NHS passport was a valuable document in the practitioners' toolbox, offering great merit in its holistic approach to patient care

For peer review only

Author	Year	Country	Aims	Participants	Methodology	Key findings
Oulton et al[38]	2018	UK	Compare and identify factors that facilitate and prevent children and young people with and without LDs and long term conditions from receiving equal access to high quality hospital care and services	Interviews: 65 staff in senior clinical or managerial roles or those employed specifically to work with children and young people with LDs. Survey: 2,261 clinical and non-clinical staff with contact with children and young people and their families	Mixed methods, framework analysis	Two key themes; national variation and staff uncertainty. Lack of knowledge about policies systems at an organisational level to support care of children and young people with LDs. Considerable variation between hospitals ranging from those appearing to have few or no systems, policies or practices in place specifically for this group, with partial systems, policies or practices in place and those with a cohesive and comprehensive level of provision. There was a lack of standardised systems in place for communicating that an individual has a LD. Also a distinct lack of systems in place for recording that an individual involved in a complaint or the subject of clinical incident has a LD
Pugely et al[66]	2014	USA	Analyse the incidence of, and risk factors for, short-term complications after paediatric deformity spinal surgery	Total: 2,005 elective cases for deformity spinal surgery in patients <19y old	Quantitative: Retrospective review of a prospective cohort using univariate and multivariate analyses	In univariate analyses developmental delay was identified as a risk factor for complications after surgery, but was not associated with mortality

For peer review only

Author	Year	Country	Aims	Participants	Methodology	Key findings
Vervloessem et al[67]	2009	Netherlands	Compare complication rates with other centres and identify risk factors for major complications related to percutaneous endoscopic gastrostomy placement in children	Total: 467 paediatric patients undergoing percutaneous endoscopic gastrostomy placement at the hospital	Quantitative: Retrospective study using univariate and multivariate analyses	When adjusted for year and ventriculoperitoneal shunt ID was not a significant risk factor for complications

Notes. ADHD = Attention Deficit Hyperactivity Disorder, ASD = Autism Spectrum Disorder, BMI = Body Mass Index, CI = Cognitive Impairment, c-section = caesarean section, DS = Downs Syndrome, HCWs = Healthcare Workers, ICU = Intensive Care Unit, ID = Intellectual Disability, IDs = Intellectual Disabilities, IDD = Intellectual and Developmental Disabilities, IV = Intravenous Tubing, LD = Learning Disability, LDs = Learning Disabilities, MDT = multidisciplinary team, PDSA = Plan Do Study Act, PPRs = Potentially Preventable Readmissions.

For peer review only

For peer review only

Appendix 4 Included grey literature material with author, webpage, year, title, aims and key information (presented in ascending date order)

Author	Webpage	Year	Title	Aims	Key information
Mencap[39]	https://www.mencap.org.uk/sites/default/files/2016-08/treat_me_right.pdf	2004	Treat me right! Better healthcare for people with a learning disability	To highlight that despite many policy reports (starting in 1992) there have been few changes in health care delivery for people with learning disabilities	Use of case studies to illustrate need for change and recommendations for change:<br>- Training for health professionals that should involve people with a learning disability.<br>- All NHS organisations must fully comply with the DDA to provide equal access to healthcare<br>- Healthcare services must address the problem of health inequalities<br>- Hospitals must fulfil their legal duty of care and provide appropriate levels of support to patients who have a learning disability<br>- There must be an inquiry into the premature deaths of people with a learning disability
Mencap[40]	https://www.mencap.org.uk/sites/default/files/2016-06/DBIreport.pdf	2007	Death by indifference	To understand the causes of deaths for people with learning disabilities in hospital	The report presents six case studies. The report highlights that the underlying cause of death for many people with a learning disability who die in hospital is the widespread ignorance and indifference throughout our healthcare services towards people with a learning disability, and their families and carers
Allyson Kent[95]	https://www.nursingtimes.net/roles/learning-disability-nurses/improving-acute-care-of-people-with-learning-disabilities-05-02-2008/	2008	Improving acute care of people with learning disabilities	To report on the development of a patient passport system for use by people with learning disabilities entering acute care settings	The patient passport is a simple tool that articulates people's individual needs and seeks to bridge the communication gap in acute care. The patient passport has evolved as a result of listening to the needs of people with learning disabilities, parents and carers and acute staff

Author	Webpage	Year	Title	Aims	Key information
Elizabeth Piper[76]	https://hydra.hull.ac.uk/assets/hull:5754a/content	2008	Investigating the experiences of people with learning disabilities in Accident & Emergency from a carer perspective	To investigate emergency healthcare - as delivered via Accident & Emergency - from the perspective of the carers of people with learning disabilities	Interview findings include the relationship staff had with both service users and carers was considered to be fundamental to a high quality service. Themes identified included Interactions that are valuing, emotional responsiveness, support, compliance and responsibilities
Sir Jonathan Michael and the Independent Inquiry into Access to Healthcare for People with Learning Disabilities[41]	https://webarchive.nationalarchives.gov.uk/20130105064250/http://www.dh.gov.uk/en/Publicationsandstatistics/Publications/PublicationsPolicyAndGuidance/DH_099255	2008	Healthcare for all: report of the independent inquiry into access to healthcare for people with learning disabilities	To review research and evidence, and the views of witnesses and stakeholders to understand the safety of care for people with learning disabilities	The report highlighted that there are risks within the care system for people with learning disabilities and these risks are exacerbated by that lack of 'reasonable adjustments' to services. A large number of avoidable deaths were also found. The report makes recommendations for changes within the healthcare system and gives examples of how to implement these
The Parliamentary and Health Service Ombudsman[42]	https://assets.publishing.service.gov.uk/government/uploads/system/uploads/attachment_data/file/250750/0203.pdf	2009	Six lives: the provision of public services to people with learning disabilities	To illustrate some significant failures in service across both health and social care for people with learning disabilities	Six investigation reports into deaths of people with learning disabilities following referral to the Ombudsman when complaints had not been satisfactorily answered by care providers;<br>The areas of concern included:<br>- Communication<br>- Partnership working and co-ordination<br>- Relationships with families and carers<br>- Failure to follow routine procedures<br>- Quality of management<br>- Advocacy<br>The case studies are powerful reminders of how things can go wrong, some with examples of unsafe care and adverse events in care delivered by NHS acute Trusts

For peer review only

Author	Webpage	Year	Title	Aims	Key information
Royal College of Nursing[96]	http://oxleas.nhs.uk/site-media/cms-downloads/RCN_Dignity_in_healthcare.pdf	2009	Dignity in health care for people with learning disabilities	To provide a resource to support nursing staff to improve dignity in health care for people with learning disabilities	Examples of good practice that makes care safer for people with learning disabilities including staff training and accessibility of care
Guidelines and Audit Implementation Network[97]	https://rqia.org.uk/RQIA/files/81/81662c46-b7bb-43a5-9496-a7f2d919c2a3.pdf	2010	Guidelines on caring for people with a learning disability in general hospital settings	To develop guidelines for care delivery to enhance safe and effective care throughout the journey within the general hospital setting for people with a learning disability	The guidelines, developed by a range of health professionals, support staff to provide safe and effective care for people with a learning disability. Guidance is given for each stage of hospital journey and include communication, attitudes, values and training for staff
The Hillingdon Hospitals NHS Foundation Trust[98]	https://www.thh.nhs.uk/about/safety/learning_dis.php<br><br>https://www.thh.nhs.uk/documents/_Patients/Guidelines_patients_learning_disabilities_Dec2011.pdf	Good Practice Guidelines - 2012	Responding to the needs of people with learning disabilities	To provide an equal service for people with learning disabilities	Examples of good practice from one Trust to improve safety and experience during hospital admission for patients with learning disabilities

For peer review only

Author	Webpage	Year	Title	Aims	Key information
The Confidential Inquiry into premature deaths of people with learning disabilities team[9]	https://www.hqip.org.uk/resource/confidential-enquiry-into-deaths-of-people-with-learning-disabilities-cipold-2013/#.XkP6jy7Fly4	2013	Confidential enquiry into deaths of people with Learning Disabilities	To investigate and compare the sequence of events leading to known deaths of 247 people, comprising people with learning disabilities and comparator cases from five Primary Care Trust areas of South West England, to assess avoidable or premature deaths	Most (96%) were of white UK ethnicity and 22% were under the age of 50 when they died. The median age of death for people with learning disabilities (65y for men; 63y for women) was significantly less than for the UK population (78y for men and 83y for women). Men with learning disabilities died, on average, 13 years sooner than men in the general population, and women with learning disabilities died 20 years sooner than women in the general population. The study revealed that the quality and effectiveness of health and social care given to people with learning disabilities was deficient in a number of ways
Agency for Healthcare Research and Quality[99]	https://www.innovations.ahrq.gov/profiles/comprehensive-program-support-patients-and-staff-improves-hospital-experience-adult	2013	Service Delivery Innovation Profile Comprehensive program to support patients and staff improves hospital experience for adult patients with intellectual and developmental disabilities	To develop a comprehensive set of strategies for patients and staff designed to improve the hospital experience for adult patients with intellectual and developmental disabilities	The program provides pre-hospitalisation tours, a telephone “helpline” to request special accommodations, procedure-specific informational booklets, participation in a buddy program, and a collection of multisensory materials for relaxation and distraction. It has been reported to improve patient experience

Author	Webpage	Year	Title	Aims	Key information
Department of Health[43]	https://assets.publishing.service.gov.uk/government/uploads/system/uploads/attachment_data/file/212292/Six_lives_2nd_Progress_Report_on_Healthcare_for_People_with_Learning_Disabilities_-_full_report.pdf	2013	Six Lives: Progress Report on Healthcare for People with Learning Disabilities	To report progress in responding to the Ombudsmen's recommendations in 2010 following the 'Six Lives' report which investigated the deaths of six people with learning disabilities, first highlighted by Mencap	Progress on issues which were of particular concern to people with learning disabilities and family carers in 2010 are reported. Issues included capacity and consent; staff understanding (including communications, information and reasonable adjustments); and complaints and advocacy
Mencap[44]	https://www.mencap.org.uk/get-involved/campaign-mencap/hear-my-voice/hear-my-voice-healthcare<br><br>https://www.youtube.com/watch?v=JZA9cClHqWA&feature=emb_logo	2014	Hear my voice: healthcare<br><br>Jayne and Jonathan's story about their brother Paul	NHS to take action to stop 1,200 preventable annual deaths of people with a learning disability in hospital by; everyone with a learning disability getting a quality annual health check, a health plan, and offered a hospital passport. GPs, doctors and nurses to also have training on reasonable adjustments they need to make to give quality care to people with a learning disability	Jayne and Jonathan shared the story of their brother Paul, who spent three weeks in intensive care before being prematurely transferred to a general ward where he experienced poor quality care, and later died. This involved; family members concerns of deterioration being disregarded, missing notes, prescribing drugs which worsened his condition and misinterpreting symptoms to be due to the learning disabilities. As a result the family initiated the campaign

Author	Webpage	Year	Title	Aims	Key information
Department of Health[100]	https://assets.publishing.service.gov.uk/government/uploads/system/uploads/attachment_data/file/309153/Strengthening_the_commitment_one_year_on_published.pdf	2014	Strengthening the Commitment: One year on: Progress report on the UK Modernising Learning Disabilities Nursing Review	To report the progress made in ensuring people with learning disabilities of all ages have access to expert learning disabilities nursing	Progress made in strengthening capacity, capability, quality and the profession are reported using recommendations and positive practice
1000 Lives Improvement, which is part of Public Health Wales[101]	http://www.1000livesplus.wales.nhs.uk/sitesplus/documents/1011/How%20to%20%2822%29%20Learning%20Disabilites%20Care%20Bundle%20web.pdf	2014	Improving general hospital care of patients who have a learning disability	To enable healthcare organisations and their teams to successfully implement a series of interventions to improve the safety and quality of care that patients with learning disabilities receive	An improvement guide that describes a care bundle of interventions and driver diagram. The guide also includes details of specific interventions in the appendices

Author	Webpage	Year	Title	Aims	Key information
Public Health England[102]	http://cdn.basw.co.uk/upload/basw_14709-8.pdf	2015	Working together 2: Easy steps to improve support for people with learning disabilities in hospital	An update of the Working together guide published in 2008 to help hospital staff, family members and paid support staff work jointly before, during and towards the end of any hospital admission (unplanned or planned) so an individual with learning disabilities could get good support and treatment	People with learning disabilities should get the help they need from health services, applying any reasonable adjustments required. Health professionals should listen more to the families and support staff of people with learning disabilities because they usually know most about the people they support and what help they need. Health staff should not assume that relatives or paid support staff of a person with learning disabilities will provide care while the person is in hospital; any such support must be discussed and agreed, taking account of their needs and supporting them appropriately. Case studies highlighted good positive practice in planning and delivering care
Patient Experience Network (not for profit organisation)<br><br>CHANGE (national human rights organisation led by disabled people)[58]	https://patientexperiencenetwork.org/wp-content/uploads/2019/10/Hidden-Voices-of-Maternity-Executive-Summary-FINAL-260815-2.pdf	2015	Hidden Voices of Maternity: Parents with Learning Disabilities Speak Out	To capture the experience of parents with learning disabilities and offer recommendations for service improvements to support care to become more person- and family-centred	Provide training for health professionals to better support parents with learning disability, improve accessibility to services. Establish a visible lead in a provider organisation whose role is to support learning disabilities as opposed to mental health or other area

For peer review only

Author	Webpage	Year	Title	Aims	Key information
Programme led by the University of Bristol and commissioned by the Healthcare Quality Improvement Partnership on behalf of NHS England[45]	https://www.bristol.ac.uk/media-library/sites/sps/leder/LeDeR annual report October 2016_FINAL v8.pdf	2016	The Learning Disabilities Mortality Review Annual Report	To evaluate the LeDeR programme	The 2015-2016 report found that there was a significantly higher mortality rate for people with learning disabilities compared to people without. Common underlying causes of mortality were circulatory and respiratory diseases and cancers. A high proportion of deaths were from causes classified as amenable to good medical care
The Westminster Commission on Autism[107]	https://westminsterautismcommission.files.wordpress.com/2016/03/ar1011_ncg-autism-report-july-2016.pdf	2016	A Spectrum of Obstacles<br><br>An Inquiry into Access to Healthcare for Autistic People	To highlight what good quality, person centred healthcare, tailored to the needs of those on the autistic spectrum, can achieve. The report is a call for ensuring equal access to quality healthcare for all on the autistic spectrum and to make this widespread and institutionalised	The report follows a seven-month inquiry chaired by Barry Sheerman MP. Consultation with over 900 autistic people, families and professionals to investigate issues highlighted in the inquiry. The report revealed obstacles that autistic people encounter when accessing healthcare, and presents six recommendations around: Training; Inspection; Data; Annual Health Checks; Leadership; and Resources.

For peer review only

Author	Webpage	Year	Title	Aims	Key information
National Quality Board[103]	https://www.england.nhs.uk/wp-content/uploads/2017/03/nqb-national-guidance-learning-from-deaths.pdf	2017	National Guidance on Learning from Deaths	To provide a framework for NHS Trusts and NHS Foundation Trusts on identifying, reporting, investigating and learning from deaths in care	Guidance to providers:<br>- All deaths of people with learning disabilities aged four years and older are subject to review using LeDeR methodology<br>- The LeDeR programme is currently being rolled out across England. Full coverage is anticipated in all Regions by the end of 2017. If there is a death of a person with learning disabilities in an acute setting in an area that is not yet covered by the LeDeR programme, Trusts are recommended to use the SJR process or a methodology of equivalent quality that meets the requirements for the data that must be collected as an interim measure
Programme led by the University of Bristol and commissioned by the Healthcare Quality Improvement Partnership on behalf of NHS England[46]	https://www.hqip.org.uk/wp-content/uploads/2018/05/LeDeR-annual-report-2016-2017-Final-6.pdf	2018	The Learning Disabilities Mortality Review Annual Report	To evaluate the LeDeR programme	The 2017 annual report suggested that 1,311 deaths were notified to the programme. Most people male (57%), single (96%) and of white ethnic background (93%). Just over a quarter had mild learning disabilities (27%), 33% had moderate learning disabilities, 29% severe learning disabilities and 11% profound or multiple learning disabilities. Approximately one in ten usually lived alone and had been in an out-of-area placement (9%)
NHS England[104]	https://www.england.nhs.uk/wp-content/uploads/2018/10/ask-listen-do-for-organisations-and-practitioners-v1.pdf	2018	Ask Listen Do Resource for Organisations and Practitioners	Tips for health, social care and education organisations and practitioners. Making feedback, concerns and complaints easier for people with a learning disability, autistic people, families and carers	This Ask, Listen, Do resource is for social care, health and education organisations, large or small, and their practitioners who support people with a learning disability, and autistic people. Four key themes within the resource; partnership, communication, processes and leadership

Author	Webpage	Year	Title	Aims	Key information
Mencap and The National Autistic Society: Treat me well campaign[105]	https://www.mencap.org.uk/get-involved/campaign-mencap/treat-me-well/announcement-learning-disability-training-health-and	2018	Oliver McGowan mandatory training in learning disability and autism	Train health and social care staff to provide better health and social care outcomes for people with a learning disability and autism, focused on raising awareness and understanding. The training is co-designed and co-delivered by people with a learning disability, autism, family carers and experts in the subject matter	A wider roll-out of the training is underway, with plans to evaluate. Mencap has already delivered the training to around 1,800 healthcare staff. 98% of participants said they wanted to change the way they deliver healthcare for people with a learning disability after taking part
Paula McGowan[48]	https://www.england.nhs.uk/blog/ask-listen-do-olivers-story/	2018	Blog<br><br>Ask Listen Do: Oliver's Story	Demonstrating the importance of the principles of Ask, Listen Do for healthcare providers of those with a learning disability to better understand and manage situations safely	Oliver had mild cerebral palsy, focal epilepsy and mild autism, and was admitted to hospital due to seizure activity. Oliver's health deteriorated and he passed away a few weeks later due to neuroleptic malignant syndrome, a rare but serious side effect of antipsychotic medications given to control his agitation in hospital, caused by his epilepsy and autism

For peer review only

Author	Webpage	Year	Title	Aims	Key information
NHS Improvement[106]	https://www.england.nhs.uk/wp-content/uploads/2020/08/v1.17_Improvement_Standards_added_note.pdf	2018	The learning disability improvement standards for NHS Trusts	Develop new standards to help NHS Trusts measure the quality of care they provide to people with learning disabilities, autism or both	The report comprises four key standards; respecting and protecting rights, inclusion and engagement, workforce and specialist learning disability services
Programme led by the University of Bristol and commissioned by the Healthcare Quality Improvement Partnership on behalf of NHS England[47]	https://www.hqip.org.uk/resource/the-learning-disabilities-mortality-review-annual-report-2018/ -.XkP6Wi7FIy4	2019	The Learning Disabilities Mortality Review Annual Report	To evaluate LeDeR programme	The 2018 annual report suggested that 4,302 deaths were notified to the programme, approximately 86% of the estimated number of deaths of people with learning disabilities in England each year. The proportion of people with learning disabilities dying in hospital is higher (62%) than in the general population (46%). Almost a half (48%) of deaths received care that the reviewer felt met or exceeded good practice. The proportion of deaths from people from Black, Asian and Minority Ethnic groups was lower (10%), than that from the population in England as a whole (14%). However, children and young people from BAME groups were overrepresented in deaths of people with learning disabilities
NHS England[83]	https://www.longtermplan.nhs.uk/publication/nhs-long-term-plan/	2019	The NHS long term plan	To set out a long term plan to make the NHS fit for the future	Providing the right care and support for children with a learning disability is part of long term plan. The plan specifies that the whole NHS will improve its understanding of the needs of people with learning disabilities and autism, and work together to improve their health and wellbeing. NHS staff will receive information and training on supporting people with a learning disability and/ or autism. National learning disability improvement standards will be implemented and will apply to all services funded by the NHS

For peer review only

Author	Webpage	Year	Title	Aims	Key information
NHS England and NHS improvement[49]	https://improvement.nhs.uk/documents/5472/190708_Patient_Safety_Strategy_for_website_v4.pdf	2019	The NHS patient safety strategy	To develop a patient safety culture and a patient safety system	Must ensure people with a learning disability are more visible; that they are listened to; and that reasonable adjustments are made to ensure they have better access to healthcare. Mandatory training on learning disability and autism to give health and care staff the knowledge and skills to accomplish this. Need understanding of safety issues: reduce harm from the effects of inappropriate psychotropic medicine use, care and treatment reviews. By 2023/24 all NHS-commissioned care will meet the learning disability improvement standards
Department of Health and Social Care[84]	https://www.gov.uk/government/consultations/learning-disability-and-autism-training-for-health-and-care-staff	2019	Consultation outcome. Learning disability and autism training for health and care staff	To gain a better understanding of how to ensure that patients and service users receive safe, effective and dignified care and that those who provide care have the knowledge, skills and behaviours to support people with learning disabilities and autistic people	Mandatory learning disability and autism training was one of the commitments made in in the Government's response to the second annual report of the LeDeR Programme. Training should focus on understanding learning disability and autism, the legislative context and making reasonable adjustments
Healthcare Safety Investigation Branch[50]	https://www.hsib.org.uk/documents/139/hsib_interim_bulletin_undiagnosed_cardiomyopathy_autism.pdf	2019	Undiagnosed cardoimyopathy of a young person with Autism	To investigate the death of an autistic patient with an undiagnosed heart problem	Identified safety issues included: The patient was not seen by a learning disability specialist and their altered physiological markers were attributed to emotional distress. Lack of national guidelines on aesthetic assessment and threshold for patients with learning disabilities and autism

For peer review only

Author	Webpage	Year	Title	Aims	Key information
Care Quality Commission[51]	https://www.cqc.org.uk/help-advice/your-stories/declare-your-care-people-learning-disabilities	2020	Declare Your Care: People with learning disabilities	A year-long, campaign focusing on four key population groups which have lower awareness of CQC	Survey findings revealed that people with a learning disability are more likely to regret not complaining about poor care than those without. The main reasons they or their carers want to raise a concern were: lack of information about a health condition and treatment options are not well explained. Stories presented
NIHR dissemination centre[52]	https://content.nihr.ac.uk/nihrdc/themedreview-04326-BCAHFA/Better-Health_Care-For-FINALWEB.pdf	2020	NIHR themed review: health and care services for people with learning disabilities	To review NIHR research on health and care services for people with learning disabilities	There is consistent evidence from confidential enquiries into unexpected deaths that general hospitals vary greatly in how well they make adjustments for patients who have learning disabilities. Research indicates problems in how hospitals implement these recommendations including confidence of general hospital staff in addressing the needs of patients. Learning disability nurses were found to make valued contributions to care. Little is known about what difference patient passports make to how well staff are able to adjust the care they provide

For peer review only

Author	Webpage	Year	Title	Aims	Key information
Programme led by the University of Bristol and commissioned by the Healthcare Quality Improvement Partnership on behalf of NHS England[11]	https://www.hqip.org.uk/wp-content/uploads/2020/07/LeDeR_2019_annual_report_FINAL.pdf	2020[a]	The Learning Disabilities Mortality Review Annual Report	To evaluate the LeDeR programme	The 2019 annual report suggested that 7,145 deaths were notified to the programme, 6,629 were adults and 516 were children (4-17y). The review process had been completed for 45% of these deaths. 58% were males; 90% were white British; 30% had mild learning disabilities, 33% had moderate learning disabilities, 27% severe learning disabilities and 10% profound and multiple learning disabilities. People with profound and multiple learning disabilities, and people from Black, Asian and Minority Ethnic groups died disproportionately at younger ages

Notes. CQC = Care Quality Commission, DDA = Disability Discrimination Act, GP = General Practitioner, LeDeR = Learning Disabilities Mortality Review, MCA = Mental Capacity Act, NHS = National Health Service, NICE = National Institute for Health and Care Excellence, NIHR = National Institute for Health Research, SJR = Structured Judgement Review.

[a]This report was published in July 2020 after our initial grey literature search.

For peer review only

Preferred Reporting Items for Systematic reviews and Meta-Analyses extension for Scoping Reviews (PRISMA-ScR) Checklist

SECTION	ITEM	PRISMA-ScR CHECKLIST ITEM	REPORTED ON PAGE #
**TITLE**			
Title	1	Identify the report as a scoping review.	1
**ABSTRACT**			
Structured summary	2	Provide a structured summary that includes (as applicable): background, objectives, eligibility criteria, sources of evidence, charting methods, results, and conclusions that relate to the review questions and objectives.	2
**INTRODUCTION**			
Rationale	3	Describe the rationale for the review in the context of what is already known. Explain why the review questions/objectives lend themselves to a scoping review approach.	4
Objectives	4	Provide an explicit statement of the questions and objectives being addressed with reference to their key elements (e.g., population or participants, concepts, and context) or other relevant key elements used to conceptualize the review questions and/or objectives.	4
**METHODS**			
Protocol and registration	5	Indicate whether a review protocol exists; state if and where it can be accessed (e.g., a Web address); and if available, provide registration information, including the registration number.	n/a
Eligibility criteria	6	Specify characteristics of the sources of evidence used as eligibility criteria (e.g., years considered, language, and publication status), and provide a rationale.	4-5
Information sources*	7	Describe all information sources in the search (e.g., databases with dates of coverage and contact with authors to identify additional sources), as well as the date the most recent search was executed.	5-6
Search	8	Present the full electronic search strategy for at least 1 database, including any limits used, such that it could be repeated.	Appendix 1
Selection of sources of evidence†	9	State the process for selecting sources of evidence (i.e., screening and eligibility) included in the scoping review.	6
Data charting process‡	10	Describe the methods of charting data from the included sources of evidence (e.g., calibrated forms or forms that have been tested by the team before their use, and whether data charting was done independently or in duplicate) and any processes for obtaining and confirming data from investigators.	6
Data items	11	List and define all variables for which data were sought and any assumptions and simplifications made.	n/a
Critical appraisal of individual sources of evidence§	12	If done, provide a rationale for conducting a critical appraisal of included sources of evidence; describe the methods used and how this information was used in any data synthesis (if appropriate).	n/a

For peer review only - http://bmjopen.bmj.com/site/about/guidelines.xhtml

SECTION	ITEM	PRISMA-ScR CHECKLIST ITEM	REPORTED ON PAGE #
Synthesis of results	13	Describe the methods of handling and summarizing the data that were charted.	6-7
**RESULTS**			
Selection of sources of evidence	14	Give numbers of sources of evidence screened, assessed for eligibility, and included in the review, with reasons for exclusions at each stage, ideally using a flow diagram.	Figure 1
Characteristics of sources of evidence	15	For each source of evidence, present characteristics for which data were charted and provide the citations.	Online supplementary Tables 1 and 2
Critical appraisal within sources of evidence	16	If done, present data on critical appraisal of included sources of evidence (see item 12).	n/a
Results of individual sources of evidence	17	For each included source of evidence, present the relevant data that were charted that relate to the review questions and objectives.	Online supplementary Tables 1 and 2
Synthesis of results	18	Summarize and/or present the charting results as they relate to the review questions and objectives.	7-17
**DISCUSSION**			
Summary of evidence	19	Summarize the main results (including an overview of concepts, themes, and types of evidence available), link to the review questions and objectives, and consider the relevance to key groups.	18
Limitations	20	Discuss the limitations of the scoping review process.	20
Conclusions	21	Provide a general interpretation of the results with respect to the review questions and objectives, as well as potential implications and/or next steps.	18-20
**FUNDING**			
Funding	22	Describe sources of funding for the included sources of evidence, as well as sources of funding for the scoping review. Describe the role of the funders of the scoping review.	1

JBI = Joanna Briggs Institute; PRISMA-ScR = Preferred Reporting Items for Systematic reviews and Meta-Analyses extension for Scoping Reviews.

* Where *sources of evidence* (see second footnote) are compiled from, such as bibliographic databases, social media platforms, and Web sites.

† A more inclusive/heterogeneous term used to account for the different types of evidence or data sources (e.g., quantitative and/or qualitative research, expert opinion, and policy documents) that may be eligible in a scoping review as opposed to only studies. This is not to be confused with *information sources* (see first footnote).

‡ The frameworks by Arksey and O'Malley (6) and Levac and colleagues (7) and the JBI guidance (4, 5) refer to the process of data extraction in a scoping review as data charting.

§ The process of systematically examining research evidence to assess its validity, results, and relevance before using it to inform a decision. This term is used for items 12 and 19 instead of "risk of bias" (which is more applicable to systematic reviews of interventions) to include and acknowledge the various sources of evidence that may be used in a scoping review (e.g., quantitative and/or qualitative research, expert opinion, and policy document).

From: Tricco AC, Lillie E, Zarin W, O'Brien KK, Colquhoun H, Levac D, et al. PRISMA Extension for Scoping Reviews (PRISMAScR): Checklist and Explanation. Ann Intern Med. 2018;169:467–473. doi: 10.7326/M18-0850.

For peer review only - http://bmjopen.bmj.com/site/about/guidelines.xhtml
