# Supplementary material for: Exploring patient safety outcomes for people with learning disabilities in acute hospital settings: a scoping review
Source: BMJ Open. 2021 May 19;11(5):e047102. doi: 10.1136/bmjopen-2020-047102 (PMC8137174; doi:10.1136/bmjopen-2020-047102)
Supplement: Reviewer comments [file bmjopen-2020-047102.reviewer_comments.pdf]

PEER REVIEW HISTORY

BMJ Open publishes all reviews undertaken for accepted manuscripts. Reviewers are asked to complete a checklist review form (**http://bmjopen.bmj.com/site/about/resources/checklist.pdf**) and are provided with free text boxes to elaborate on their assessment. These free text comments are reproduced below.

ARTICLE DETAILS

**TITLE (PROVISIONAL)**	Exploring patient safety outcomes for people with learning disabilities in acute hospital settings: A scoping review
**AUTHORS**	Louch, Gemma; Albutt, Abigail; Joanna Harlow-Trigg, Joanna; Moore, Sally; Smyth, Kate; Ramsey, Lauren; O'Hara, Jane

VERSION 1 – REVIEW

**REVIEWER**	Vincent, Charles<br>University of Oxford, Experimental Psychology
**REVIEW RETURNED**	20-Dec-2020

**GENERAL COMMENTS**	This narrative review addresses the important topic of the safety of people with learning disabilities while in hospital. The review is comprehensive, conducted with admirable rigour and encompasses both the main and grey literature. The appendices provide a clear account of the search methods and the characteristics of the papers. The authors did not attempt a formal synthesis or carry out quality checks on the papers but their approach seems appropriate given the diverse and variable literature. The scoping review achieves its objectives in assembling the relevant literature and identifying the main themes and findings.<br><br>This is a valuable paper on an important, in fact neglected, topic. However, I think the authors could be considerably more robust in their conclusions. Admittedly, this is a scoping review but my reading of the material presented is that there is ample evidence that people with learning disabilities are at high risk in hospital and that very little has been done to protect them, other than rely on their families. The authors could strengthen both the results and the discussion to set out their conclusions more clearly and more forcefully.<br><br>Specific suggestions<br><br>• The authors set out three aims, which are clear and to the point. It would be helpful if the results section followed the aims. It would be much clearer, for instance, if all the material showing harms of various kinds were brought together in a single section, with sub-headings. Then a section on explanations for harms, and finally one in interventions, again with sub-headings.<br><br>• I think the paper would have more impact and be easier to follow if the authors added some boxes and tables within the text. For instance, a box setting out the main types of harms, another on potential interventions and so on. The material is all there, but they

	could help the reader by summarising and presenting it in a simple format.<br><br>• The material in the discussion is perfectly reasonable but rather tentative in tone. I suggest that the Discussion might follow the aims. First set out the harms and risks clearly, and then consider why people with learning disabilities are at risk. I appreciate the framework of gaps is relevant here, but presumably that is only one reason they are vulnerable. Setting out a research agenda is certainly important but more could be said on the immediate implications. What interventions have shown promise? What do the authors think hospitals could do to protect these vulnerable people? I realise these are big questions, but I suspect the authors, having been through all this material, have some pretty good ideas.

**REVIEWER**	Carson-Stevens, A<br>Cardiff University
**REVIEW RETURNED**	04-Mar-2021

**GENERAL COMMENTS**	Thank you for the opportunity to review this clear and eloquently written scoping review and narrative synthesis exploring safety outcomes for people with learning disabilities in acute hospital settings.<br><br>I am fully aware of the excellent work led by Professor O'Hara's group but have no conflicts of interest to report.<br><br>Overall I found the review relatively faultless but would encourage the team to review the PRISMA diagram and briefly expand on their policy and practice recommendations in the discussion.<br><br>Introduction:<br>There is a clear rationale with relevant evidence cited to justify the need for a review of this nature. The aims and objectives are succinctly stated.<br><br>Method: The review has followed the appropriate standards and a checklist is included as a supplementary file. A detailed and structured approach has been adopted.<br><br>Results: Engaging description with relevant key details (study type, population specifics and what was identified) from included studies.<br><br>In the PRISMA, include a box indicating the number of the 'full-text articles' assessed for eligibility that made it through. Given articles were included for more than one reason it makes it hard to track whether the database searches yielded any relevant articles included in the review.<br><br>Discussion: Appropriate conclusions drawn. Findings sufficiently contextualised to existing literature. The research implications are stated.<br><br>I would welcome any immediate policy or practice improvement recommendations to be flagged here too. Could they expand, for example, on how 'attention [can] be given...to existing recommendations" and what do they mean by 'policy level action' - call it out.

VERSION 1 – AUTHOR RESPONSE

Response to Reviewers

Thank you for the feedback from the Editor and Reviewers. We are extremely grateful for the positive comments and the extremely useful feedback which we feel has strengthened the manuscript.

**Comment**	**Response**
**Reviewer 1**	
The authors set out three aims, which are clear and to the point. It would be helpful if the results section followed the aims. It would be much clearer, for instance, if all the material showing harms of various kinds were brought together in a single section, with sub-headings. Then a section on explanations for harms, and finally one in interventions, again with sub-headings.	Thank you for this feedback. We have amended the manuscript as suggested, and mapped on how the key concepts relate to the three review objectives.
I think the paper would have more impact and be easier to follow if the authors added some boxes and tables within the text. For instance, a box setting out the main types of harms, another on potential interventions and so on. The material is all there, but they could help the reader by summarising and presenting it in a simple format.	Thank you for this feedback. We have amended the manuscript as suggested.
The material in the discussion is perfectly reasonable but rather tentative in tone. I suggest that the Discussion might follow the aims. First set out the harms and risks clearly, and then consider why people with learning disabilities are at risk. I appreciate the framework of gaps is relevant here, but presumably that is only one reason they are vulnerable. Setting out a research agenda is certainly important but more could be said on the immediate implications. What interventions have shown promise? What do the authors think hospitals could do to protect these vulnerable people? I realise these are big questions, but I suspect the authors, having been through all this material, have some pretty good ideas.	Thank you for this feedback. We have amended the discussion to present findings around the three aims. In addition, we have expanded on the immediate implications and promising interventions, and tweaked the tone to be more forthcoming in our observations and conclusions.
**Reviewer 2**	
In the PRISMA, include a box indicating the number of the 'full-text articles' assessed for eligibility that made it through. Given articles were included for more than one reason it	Thank you for this observation, we have amended this as suggested.

makes it hard to track whether the database searches yielded any relevant articles included in the review.	
Discussion:<br><br>I would welcome any immediate policy or practice improvement recommendations to be flagged here too. Could they expand, for example, on how 'attention [can] be given...to existing recommendations" and what do they mean by 'policy level action' - call it out.	Thank you for this feedback, which chimes with feedback from Reviewer 1. We have added more information about immediate implications and have expanded on the points highlighted.

VERSION 2 – REVIEW

**REVIEWER**	Vincent, Charles<br>University of Oxford, Experimental Psychology
**REVIEW RETURNED**	21-Apr-2021

**GENERAL COMMENTS**	I thank the authors for their thoughtful response to the reviews and the strengthened version of this valuable paper.
